# Characterisation of in-hospital complications associated with COVID-19 using the ISARIC WHO Clinical Characterisation Protocol UK: a prospective, multicentre cohort study

**DOI:** 10.1016/S0140-6736(21)00799-6

**Published:** 2021-07-17

**Authors:** Thomas M Drake, Aya M Riad, Cameron J Fairfield, Conor Egan, Stephen R Knight, Riinu Pius, Hayley E Hardwick, Lisa Norman, Catherine A Shaw, Kenneth A McLean, A A Roger Thompson, Antonia Ho, Olivia V Swann, Michael Sullivan, Felipe Soares, Karl A Holden, Laura Merson, Daniel Plotkin, Louise Sigfrid, Thushan I de Silva, Michelle Girvan, Clare Jackson, Clark D Russell, Jake Dunning, Tom Solomon, Gail Carson, Piero Olliaro, Jonathan S Nguyen-Van-Tam, Lance Turtle, Annemarie B Docherty, Peter JM Openshaw, J Kenneth Baillie, Ewen M Harrison, Malcolm G Semple, ISARIC4C investigators, J Kenneth Baillie, Malcolm G Semple, Peter JM Openshaw, Gail Carson, Beatrice Alex, Benjamin Bach, Wendy S Barclay, Debby Bogaert, Meera Chand, Graham S Cooke, Annemarie B Docherty, Jake Dunning, Ana da Silva Filipe, Tom Fletcher, Christopher A Green, Ewen M Harrison, Julian A Hiscox, Antonia YW Ho, Peter W Horby, Samreen Ijaz, Say Khoo, Paul Klenerman, Andrew Law, Wei Shen Lim, Alexander J Mentzer, Laura Merson, Alison M Meynert, Mahdad Noursadeghi, Shona C Moore, Massimo Palmarini, William A Paxton, Georgios Pollakis, Nicholas Price, Andrew Rambaut, David L Robertson, Clark D Russell, Vanessa Sancho-Shimizu, Janet T Scott, Thushan de Silva, Louise Sigfrid, Tom Solomon, Shiranee Sriskandan, David Stuart, Charlotte Summers, Richard S Tedder, Emma C Thomson, AA Roger Thompson, Ryan S Thwaites, Lance CW Turtle, Rishi K Gupta, Carlo Palmieri, Olivia V Swann, Maria Zambon, Marc-Emmanuel Dumas, Julian Griffin, Zoltan Takats, Kanta Chechi, Petros Andrikopoulos, Anthonia Osagie, Michael Olanipekun, Sonia Liggi, Matthew Lewis, Gonçalo dos Santos Correia, Caroline Sands, Panteleimon Takis, Lynn Maslen, Hayley Hardwick, Chloe Donohue, Fiona Griffiths, Wilna Oosthuyzen, Lisa Norman, Riinu Pius, Thomas M Drake, Cameron J Fairfield, Stephen R Knight, Kenneth A Mclean, Derek Murphy, Catherine A Shaw, Jo Dalton, Michelle Girvan, Egle Saviciute, Stephanie Roberts, Janet Harrison, Laura Marsh, Marie Connor, Sophie Halpin, Clare Jackson, Carrol Gamble, Daniel Plotkin, James Lee, Gary Leeming, Andrew Law, Murray Wham, Sara Clohisey, Ross Hendry, James Scott-Brown, William Greenhalf, Victoria Shaw, Sarah E McDonald, Seán Keating, Katie A. Ahmed, Jane A Armstrong, Milton Ashworth, Innocent G Asiimwe, Siddharth Bakshi, Samantha L Barlow, Laura Booth, Benjamin Brennan, Katie Bullock, Benjamin WA Catterall, Jordan J Clark, Emily A Clarke, Sarah Cole, Louise Cooper, Helen Cox, Christopher Davis, Oslem Dincarslan, Chris Dunn, Philip Dyer, Angela Elliott, Anthony Evans, Lorna Finch, Lewis WS Fisher, Terry Foster, Isabel Garcia-Dorival, William Greenhalf, Philip Gunning, Catherine Hartley, Rebecca L Jensen, Christopher B Jones, Trevor R Jones, Shadia Khandaker, Katharine King, Robyn T. Kiy, Chrysa Koukorava, Annette Lake, Suzannah Lant, Diane Latawiec, Lara Lavelle-Langham, Daniella Lefteri, Lauren Lett, Lucia A Livoti, Maria Mancini, Sarah McDonald, Laurence McEvoy, John McLauchlan, Soeren Metelmann, Nahida S Miah, Joanna Middleton, Joyce Mitchell, Shona C Moore, Ellen G Murphy, Rebekah Penrice-Randal, Jack Pilgrim, Tessa Prince, Will Reynolds, P. Matthew Ridley, Debby Sales, Victoria E Shaw, Rebecca K Shears, Benjamin Small, Krishanthi S Subramaniam, Agnieska Szemiel, Aislynn Taggart, Jolanta Tanianis-Hughes, Jordan Thomas, Erwan Trochu, Libby van Tonder, Eve Wilcock, J. Eunice Zhang, Lisa Flaherty, Nicole Maziere, Emily Cass, Alejandra Doce Carracedo, Nicola Carlucci, Anthony Holmes, Hannah Massey, Lee Murphy, Nicola Wrobel, Sarah McCafferty, Kirstie Morrice, Alan MacLean, Kayode Adeniji, Daniel Agranoff, Ken Agwuh, Dhiraj Ail, Erin L. Aldera, Ana Alegria, Brian Angus, Abdul Ashish, Dougal Atkinson, Shahedal Bari, Gavin Barlow, Stella Barnass, Nicholas Barrett, Christopher Bassford, Sneha Basude, David Baxter, Michael Beadsworth, Jolanta Bernatoniene, John Berridge, Nicola Best, Pieter Bothma, David Chadwick, Robin Brittain-Long, Naomi Bulteel, Tom Burden, Andrew Burtenshaw, Vikki Caruth, David Chadwick, Duncan Chambler, Nigel Chee, Jenny Child, Srikanth Chukkambotla, Tom Clark, Paul Collini, Catherine Cosgrove, Jason Cupitt, Maria-Teresa Cutino-Moguel, Paul Dark, Chris Dawson, Samir Dervisevic, Phil Donnison, Sam Douthwaite, Andrew Drummond, Ingrid DuRand, Ahilanadan Dushianthan, Tristan Dyer, Cariad Evans, Chi Eziefula, Chrisopher Fegan, Adam Finn, Duncan Fullerton, Sanjeev Garg, Sanjeev Garg, Atul Garg, Effrossyni Gkrania-Klotsas, Jo Godden, Arthur Goldsmith, Clive Graham, Elaine Hardy, Stuart Hartshorn, Daniel Harvey, Peter Havalda, Daniel B Hawcutt, Maria Hobrok, Luke Hodgson, Anil Hormis, Michael Jacobs, Susan Jain, Paul Jennings, Agilan Kaliappan, Vidya Kasipandian, Stephen Kegg, Michael Kelsey, Jason Kendall, Caroline Kerrison, Ian Kerslake, Oliver Koch, Gouri Koduri, George Koshy, Shondipon Laha, Steven Laird, Susan Larkin, Tamas Leiner, Patrick Lillie, James Limb, Vanessa Linnett, Jeff Little, Mark Lyttle, Michael MacMahon, Emily MacNaughton, Ravish Mankregod, Huw Masson, Elijah Matovu, Katherine McCullough, Ruth McEwen, Manjula Meda, Gary Mills, Jane Minton, Mariyam Mirfenderesky, Kavya Mohandas, Quen Mok, James Moon, Elinoor Moore, Patrick Morgan, Craig Morris, Katherine Mortimore, Samuel Moses, Mbiye Mpenge, Rohinton Mulla, Michael Murphy, Megan Nagel, Thapas Nagarajan, Mark Nelson, Lillian Norris, Matthew K. O'Shea, Igor Otahal, Marlies Ostermann, Mark Pais, Carlo Palmieri, Selva Panchatsharam, Danai Papakonstantinou, Hassan Paraiso, Brij Patel, Natalie Pattison, Justin Pepperell, Mark Peters, Mandeep Phull, Stefania Pintus, Jagtur Singh Pooni, Frank Post, David Price, Rachel Prout, Nikolas Rae, Henrik Reschreiter, Tim Reynolds, Neil Richardson, Mark Roberts, Devender Roberts, Alistair Rose, Guy Rousseau, Brendan Ryan, Taranprit Saluja, Aarti Shah, Prad Shanmuga, Anil Sharma, Anna Shawcross, Jeremy Sizer, Manu Shankar-Hari, Richard Smith, Catherine Snelson, Nick Spittle, Nikki Staines, Tom Stambach, Richard Stewart, Pradeep Subudhi, Tamas Szakmany, Kate Tatham, Jo Thomas, Chris Thompson, Robert Thompson, Ascanio Tridente, Darell Tupper-Carey, Mary Twagira, Nick Vallotton, Rama Vancheeswaran, Lisa Vincent-Smith, Shico Visuvanathan, Alan Vuylsteke, Sam Waddy, Rachel Wake, Andrew Walden, Ingeborg Welters, Tony Whitehouse, Paul Whittaker, Ashley Whittington, Padmasayee Papineni, Meme Wijesinghe, Martin Williams, Lawrence Wilson, Sarah Sarah, Stephen Winchester, Martin Wiselka, Adam Wolverson, Daniel G Wootton, Andrew Workman, Bryan Yates, Peter Young

**Affiliations:** a Centre for Medical Informatics, Usher Institute, University of Edinburgh, Edinburgh, UK; b Department of Child Life and Health, University of Edinburgh, Edinburgh, UK; c Roslin Institute, University of Edinburgh, Edinburgh, UK; d Centre for Inflammation Research, The Queen's Medical Research Institute, University of Edinburgh, Edinburgh, UK; e Health Protection Research Unit in Emerging and Zoonotic Infections, Institute of Infection, Veterinary and Ecological Sciences, Faculty of Health and Life Sciences, University of Liverpool, Liverpool, UK; f Liverpool Clinical Trials Centre, University of Liverpool, Liverpool, UK; g Clinical Infection Microbiology and Immunology, Institute of Infection, Veterinary, and Zoological Science, University of Liverpool, Liverpool, UK; h Department of Infection, Immunity and Cardiovascular Disease, University of Sheffield, Sheffield, UK; i Medical Research Council-University of Glasgow Centre for Virus Research, University of Glasgow, Glasgow, UK; j Institute of Cardiovascular and Medical Sciences, University of Glasgow, Glasgow, UK; k Paediatric Infectious Diseases, Royal Hospital for Sick Children, Edinburgh, UK; l Department of Respiratory Medicine, Alder Hey Children's Hospital, Liverpool, UK; m Centre for Tropical Medicine and Global Health, Nuffield Department of Medicine, University of Oxford, Oxford, UK; n Emerging Infections and Zoonoses Unit, National Infection Service, Public Health England, London, UK; o Department of Neurology, The Walton Centre NHS Foundation Trust, Liverpool, UK; p Division of Epidemiology and Public Health, School of Medicine, University of Nottingham, Nottingham, UK; q United Kingdom Department of Health and Social Care, London, UK; r National Heart and Lung Institute, Imperial College London, London, UK

## Abstract

**Background:**

COVID-19 is a multisystem disease and patients who survive might have in-hospital complications. These complications are likely to have important short-term and long-term consequences for patients, health-care utilisation, health-care system preparedness, and society amidst the ongoing COVID-19 pandemic. Our aim was to characterise the extent and effect of COVID-19 complications, particularly in those who survive, using the International Severe Acute Respiratory and Emerging Infections Consortium WHO Clinical Characterisation Protocol UK.

**Methods:**

We did a prospective, multicentre cohort study in 302 UK health-care facilities. Adult patients aged 19 years or older, with confirmed or highly suspected SARS-CoV-2 infection leading to COVID-19 were included in the study. The primary outcome of this study was the incidence of in-hospital complications, defined as organ-specific diagnoses occurring alone or in addition to any hallmarks of COVID-19 illness. We used multilevel logistic regression and survival models to explore associations between these outcomes and in-hospital complications, age, and pre-existing comorbidities.

**Findings:**

Between Jan 17 and Aug 4, 2020, 80 388 patients were included in the study. Of the patients admitted to hospital for management of COVID-19, 49·7% (36 367 of 73 197) had at least one complication. The mean age of our cohort was 71·1 years (SD 18·7), with 56·0% (41 025 of 73 197) being male and 81·0% (59 289 of 73 197) having at least one comorbidity. Males and those aged older than 60 years were most likely to have a complication (aged ≥60 years: 54·5% [16 579 of 30 416] in males and 48·2% [11 707 of 24 288] in females; aged <60 years: 48·8% [5179 of 10 609] in males and 36·6% [2814 of 7689] in females). Renal (24·3%, 17 752 of 73 197), complex respiratory (18·4%, 13 486 of 73 197), and systemic (16·3%, 11 895 of 73 197) complications were the most frequent. Cardiovascular (12·3%, 8973 of 73 197), neurological (4·3%, 3115 of 73 197), and gastrointestinal or liver (0·8%, 7901 of 73 197) complications were also reported.

**Interpretation:**

Complications and worse functional outcomes in patients admitted to hospital with COVID-19 are high, even in young, previously healthy individuals. Acute complications are associated with reduced ability to self-care at discharge, with neurological complications being associated with the worst functional outcomes. COVID-19 complications are likely to cause a substantial strain on health and social care in the coming years. These data will help in the design and provision of services aimed at the post-hospitalisation care of patients with COVID-19.

**Funding:**

National Institute for Health Research and the UK Medical Research Council.

## Introduction

Many people across the world have been hospitalised with COVID-19 following SARS-CoV-2 infection. Evidence has established that these patients have high mortality rates (26%), and up to 17% of patients admitted to hospital will require ventilatory support and critical care.^1^ Several case reports, cross-sectional studies, and case-control studies have described the presence of non-respiratory complications in those with COVID-19 and suggest that these are likely to be associated with poor outcomes.^2^, ^3^, ^4^

Understanding the possible complications of COVID-19 is important for patient management and provision in health-care systems. For patients, information around in-hospital complication rates are important for decision making about treatment, long-term planning, possible resumption of normal activity and, more recently, vaccination. For health-care systems, these data are vital to inform immediate preparedness measures (ie, allocation of resources, equipment, and staffing) and also for long-term planning of health-care delivery to a population that might have incurred additional morbidity due to COVID-19.

**Research in context**

**Evidence before this study**

We did a systematic search of the MEDLINE and PubMed databases on Dec 5, 2020, using the search terms (“in-hospital” OR “hospital”) AND (“SARS-CoV-2” OR “COVID” OR “COVID-19”) AND “complications”. We limited dates of searches from Jan 1, 2020, to the date the search was conducted. No language restrictions applied. Data from other areas of health care, such as surgery, suggest that patients with COVID-19 are at greater risk of subsequent complications, but systematic characterisation of complications in these patients has not yet been undertaken in large multicentre studies of patients admitted to hospital. Most COVID-19 studies have focused on mortality and respiratory support outcomes. Characterising the burden of complications is important for health-care system preparedness for further waves of infection, determining future population morbidity, understanding the full repercussions of COVID-19 for society, and for informing future research and clinical guidelines. The current literature is comprised of several small cohort or case-control studies that focus on specific organ systems or conditions. There are few prospective systematically collected data describing the in-hospital complications of COVID-19.

**Added value of this study**

Hospitalised adult patients aged 19 and over with COVID-19 frequently had complications, even in younger age groups and in those with few pre-existing comorbidities. Occurrence of complications was associated with a significantly reduced ability to self-care at discharge, which was seen in all age and comorbidity groups. Although patients aged younger than 50 years are at low risk of dying from COVID-19, we found high rates of complications across all age groups.

**Implications of all the available evidence**

In patients admitted to hospital with COVID-19, there is a burden of immediate complications affecting all age groups. Many of the complications identified are likely to have important long-term effects. Health-care systems and policy makers should prepare for increases in population morbidity arising from COVID-19 and its subsequent complications. As complications following COVID-19 are common across all age groups and comorbidities, public health messaging around the risk COVID-19 poses to younger otherwise healthy people should be considered alongside vaccine prioritisation. Further studies are required to understand the medium-term to long-term effects of COVID-19 and how immediate complications may lead to lasting morbidity.

A substantial proportion of patients with COVID-19 go on to develop critical illness and require organ support. It is widely recognised that survival following critical illness is accompanied by a substantial burden of additional physical and mental health morbidity that cannot be measured by mortality outcomes.^5^, ^6^ Mortality has been widely used as an outcome in epidemiological studies and randomised controlled trials for patients with COVID-19 but fails to capture the immediate short-term health issues faced by survivors, including in-hospital complications and functional outcomes. In patients with COVID-19 undergoing surgery, high rates of post-procedural mortality and complications have been noted, but systematic characterisation of hospitalised patients with COVID-19 is lacking.^7^ In other non-SARS-CoV-2 viral illnesses, for example influenza, short-term complications such as myocardial infarction, acute kidney injury, and stroke are common and can cause greater morbidity than the initial infection itself.^6^, ^8^, ^9^, ^10^, ^11^ Understanding which patients develop short-term complications might also allow clinicians and researchers to develop care pathways and interventions to mitigate the impact of complications. As many patients with COVID-19 are critically unwell, identifying the burden of short-term morbidity could be useful to understand the long-term burden on health-care systems and society for those who survive COVID-19.

We have previously characterised the clinical features of patients admitted to hospital with COVID-19 using the International Severe Acute Respiratory and Emerging Infections Consortium (ISARIC) WHO Clinical Characterisation Protocol UK (CCP-UK) for severe emerging infections.^1^ The aim of this study was to describe the short-term complications, beyond those associated with the presenting features of COVID-19 and severe acute respiratory infection.

## Methods

### Study design and participants

The ISARIC WHO CCP-UK protocol was developed by an international consensus in 2012–14 and reactivated in response to the COVID-19 pandemic on Jan 17, 2020.^12^ Our study is an actively recruiting prospective cohort study across 302 health-care facilities in the UK. Adult patients aged 19 years and older, who were admitted to hospital between Jan 17 and Aug 4, 2020, with confirmed or highly suspected SARS-CoV-2 infection leading to COVID-19 were included in this analysis; overall study recruitment is ongoing. We used this WHO age cutoff^13^ as children exhibit other patterns of complications including multisystem inflammatory syndrome. Confirmation of SARS-CoV-2 was done using RT-PCR. Highly suspected cases were eligible for inclusion, given that SARS-CoV-2 was an emergent pathogen at the time of protocol activation and laboratory confirmation was dependent on local availability of testing.

Study materials including protocol, revision history, case report forms, study information, and consent forms are available online.^14^ All patients who provided biological samples were required to provide informed, written consent. If patients only provided routinely collected clinical data, written consent was not required. Ethical approval was given by the South Central–Oxford C Research Ethics Committee in England (reference 13/SC/0149) and the Scotland A Research Ethics Committee (reference 20/SS/0028). The study is reported in line with the Strengthening the Reporting of Observational Studies in Epidemiology guidelines.^15^

### Procedures

Data collected by research nurses and volunteer medical students were entered into a standardised electronic case report form within a secure research electronic data capture database.^16^ Multiple timepoints were captured, including admission, hospital stay at days 1, 3, and 9, and discharge or status at 28 days if not discharged. Data were collected according to a detailed protocol, which was updated to reflect developments over the course of the pandemic. Participant characteristics including age, sex at birth, physiological parameters at presentation, and comorbidities were also recorded. Comorbidities included asthma, chronic cardiac disease, chronic haematological disease, chronic kidney disease, chronic neurological disease, chronic pulmonary disease, HIV/AIDS, history of malignancy, liver disease, clinician-defined obesity, rheumatological disorders, and smoking. Deprivation was calculated by mapping individual postcodes to their corresponding Index of Multiple Deprivation (IMD) using the Office for National Statistics postcode data. Using national data, we calculated deprivation quintiles, with the first quintile being the least deprived and the fifth quintile the most deprived. For patients where postcodes were missing, the average IMD rank, weighted by population in each lower super output area for a given hospital catchment area, was used.

### Outcomes

The primary outcome of this study was the incidence of in-hospital complications, defined as organ-specific diagnoses occurring alone or in addition to any hallmarks of COVID-19 illness (appendix p 1–2). All complications were recorded so that total morbidity could be described, not just those directly attributable to COVID-19. Although COVID-19 is a multisystem disease, severe respiratory infection was considered characteristic of COVID-19 and was not regarded as a complication. Data were collected on organ-specific complications including complex respiratory (bacterial pneumonia, acute respiratory distress syndrome [ARDS], empyema, pneumothorax, and pleural effusion), neurological (meningitis, encephalitis, seizure, and stroke), cardiovascular (thromboembolism, heart failure, myocarditis, endocarditis, arrhythmia, cardiomyopathy, myocardial ischaemia, and cardiac arrest), acute kidney injury, gastrointestinal (acute liver injury, pancreatitis, and gastrointestinal haemorrhage), and other systemic complications (coagulopathy, disseminated intravascular coagulation, anaemia, and bloodstream infection). The occurrence of complications was determined from routine clinical records by local investigators with the exceptions of bloodstream infection and microbiologically confirmed bacterial pneumonia. These were defined based on recorded results from sputum, deep respiratory, or blood cultures and restricted to instances where clinically significant organisms were detected in the sample. Blood stream infection was defined as growth of clinically significant bacteria (excluding coagulase-negative *Staphylococci*) or fungus recorded from blood culture or PCR of the blood. Results considered to represent contamination or colonisation were excluded. Owing to the difficulties of obtaining lower respiratory tract samples to confirm bacterial pneumonia and the low positivity rates, we present both highly likely and suspected bacterial pneumonia in the appendix (pp 1–2).

The existence of likely ARDS was described clinically or defined as one of the following combinations: receiving extracorporeal membrane oxygenation; being nursed in a prone position and receiving invasive mechanical ventilation; or receiving mechanical ventilation with a ratio of partial pressure of arterial oxygen to fraction of inspired air of 300 mm Hg or less. For acute kidney injury and acute liver injury, we used laboratory measurements with internationally recognised grading systems to detect complications that could have been missed. Acute kidney injury was defined as a creatinine rise which corresponded to the Kidney Disease Improving Global Outcomes stage 1 or above definition^17^ (creatinine rise ≥1·5 × baseline value or by ≥26·5 μmol/L). We did not incorporate urine output into this definition as this parameter is not universally recorded for all patients, particularly outwith critical care. Acute liver injury was defined as one of the following: an international normalised ratio rise of 2·5 times or greater than the lowest entered value; an international normalised ratio of more than 4·5 (in the absence of warfarin therapy); an alanine aminotransferase rise of more than 10 times the lowest value; an alanine aminotransferase of more than 150 IU/L; a bilirubin rise of more than 15 μmol/L; or a bilirubin greater than 55 μmol/L (in the absence of any pre-existing liver disease). In those who survived, we also captured information on whether self-care ability was the same or worse than before hospital admission at time of discharge, defined clinically as the change in support required before and after hospital admission. For this outcome, if patients required ongoing hospital care, we defined this outcome as worse than before onset of COVID-19 illness due to these ongoing care requirements.

### Statistical analysis

Continuous data are presented as a mean with SD where data are normally distributed and as a median with the 25th and 75th centiles for non-parametric data. Categorical data are summarised as frequencies and percentages. Differences between groups for continuous normally distributed data were tested using Welch's *t* test for two groups or ANOVA when there were more than two groups. Non-parametric continuous data were tested using a Mann-Whitney *U* test for two groups or Kruskall-Wallis test for three or more groups. Differences across categorical data were tested using the χ^2^ test or Fisher's exact test when expected cell counts were less than five. Analysis of complication co-occurrence was done using the Jaccard similarity index and represented visually as heatmaps with dendrograms constructed from complete hierarchical clustering results. We only included patients who had completed outcomes, with at least 2 months of follow-up. There were low rates of missing data and therefore multiple imputation was not used.

To explore if the number of complications and which specific complications were associated with mortality (dependent variable), complication variables were entered independently into Cox proportional hazards models and adjusted for other potentially confounding factors. These data were described using Kaplan-Meier plots and modelled using Cox proportional hazards regression. Reported date of symptom onset was taken as day 0. Discharge from hospital was considered an absorbing state (once discharged, patients were considered no longer at risk of death); thus discharge did not compete with death. The proportional hazards assumption was checked.

To observe whether complications were associated with increased severity of initial disease, we used the ISARIC 4C Mortality Score, quick sequential organ failure assessment (qSOFA), and National Early Warning Score 2 (NEWS2) on admission or time of symptom start to examine the relationship between severity and presence of any in-hospital complications.^18^ These scores are commonly used in clinical practice to identify patients with deteriorating or critical illness and risk of subsequent death in general adult hospital populations (NEWS2 and qSOFA) or in COVID-19 patients (4C Mortality Score). We calculated the score for each adult patient in the dataset and plotted each score against the observed incidence of complications in each score group.

Multilevel logistic regression models were constructed to identify associations between patient characteristics (potential confounders, including patient demographics and existing comorbidities) and the development of specific complications, worse self-care ability on discharge, and the requirement for ongoing hospital care. For all models, variable selection was done based on clinical plausibility, and final models were selected based on clinical relevance guided by minimisation of the Akaike information criterion. Centre-level variation was accounted for using mixed-effects models that included hospital as a random effect and patient-level variables as fixed effects. We did stratified analyses to focus on survivors and on those admitted to critical care.

To identify which patient groups are at the highest risk of complications and mortality, we used generalised additive models and generated risk estimates by age, sex, and comorbidity status. Generalised additive models accommodated potential non-linear relationships between variables with the inclusion of penalised thin-plate regression splines on continuous variables. We did this for each organ-specific complication outcome, as well as testing the associations between organ-specific complications and death. Models were adjusted for age, sex, comorbidity status and deprivation (IMD quintile). First and second order interactions were explored and included where they significantly contributed to model fitting. We ran 100 bootstrap replicates for each model to provide a visual representation of the distribution.

All statistical analyses were done with R (version 3.6.3) using the tidyverse, finalfit, mcgv, survival, stringdist, janitor, and Hmisc packages.

### Role of the funding source

The funders of the study had no role in the study design, data collection, data analysis, data interpretation, or writing of the report.

## Results

**Figure 1:**
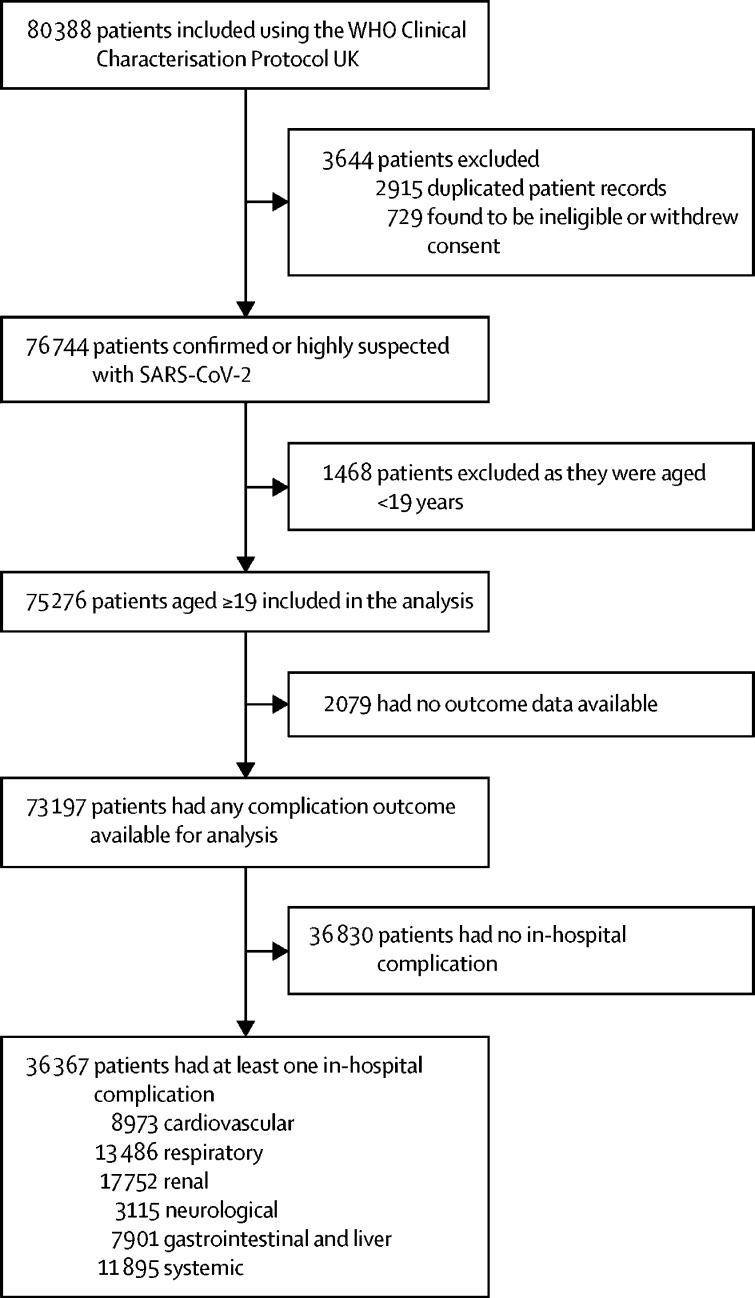
Study profile

Between Jan 17 and Aug 4, 2020, 80 388 patients were included in the CCP-UK study (figure 1). Of these, 75 276 were adults aged 19 years or older, of which 97·2% (73 197 of 75 276) had any complication outcome available for analysis. The overall mortality rate was 31·5% (23 092 of 73 197), and the overall complication rate was 49·7% (36 367 of 73 197 had at least one complication). In surviving patients, 43·5% (21 784 of 50 105) had at least one complication. Proportions of patients having at least one complication were highest in age groups of over 60 years (table 1). Missing data for each variable were under 10% for nearly all patient characteristics included in the study (appendix pp 3–4). Of all patients included, 85·9% (62 894 of 73 197) had a positive SARS-CoV-2 RT-PCR test. Patients who did not have a positive swab had the same or slightly lower rates of complications overall and organ-specific complications (appendix p 3).

**Table 1:** Patient characteristics by organ-specific complications

		**Patients having complications**		**Organ-specific complications**					
		Total number of patients	Any complication	Systemic	Renal	Gastrointestinal (including liver)	Cardiovascular	Neurological	Respiratory*
Total number of patients		73 197	36 367 (49·7%)	11 895 (16·3%)	17 752 (24·3%)	7901 (10·8%)	8973 (12·3%)	3115 (4·3%)	13 486 (18·4%)
Age on admission, years									
	19–29	1500 (2·0%)	411 (1·1%)	147 (1·2%)	126 (0·7%)	139 (1·8%)	47 (0·5%)	38 (1·2%)	145 (1·1%)
	30–39	2753 (3·8%)	1015 (2·8%)	376 (3·2%)	353 (2·0%)	365 (4·6%)	134 (1·5%)	91 (2·9%)	457 (3·4%)
	40–49	4996 (6·8%)	2170 (6·0%)	731 (6·1%)	874 (4·9%)	740 (9·4%)	370 (4·1%)	162 (5·2%)	1169 (8·7%)
	50–59	9101 (12·4%)	4418 (12·1%)	1504 (12·6%)	2078 (11·7%)	1468 (18·6%)	847 (9·4%)	352 (11·3%)	2263 (16·8%)
	60–69	11 139 (15·2%)	5954 (16·4%)	2008 (16·9%)	3055 (17·2%)	1578 (20·0%)	1389 (15·5%)	500 (16·1%)	2767 (20·5%)
	70–79	16 563 (22·6%)	8549 (23·5%)	2727 (22·9%)	4318 (24·3%)	1644 (20·8%)	2220 (24·7%)	725 (23·3%)	2978 (22·1%)
	80–89	19 900 (27·2%)	10 207 (28·1%)	3241 (27·2%)	5161 (29·1%)	1478 (18·7%)	2888 (32·2%)	941 (30·2%)	2761 (20·5%)
	≥90	7245 (9·9%)	3643 (10·0%)	1161 (9·8%)	1787 (10·1%)	489 (6·2%)	1078 (12·0%)	306 (9·8%)	946 (7·0%)
Sex at birth									
	Female	31 977 (43·7%)	14 521 (39·9%)	4872 (41·0%)	6612 (37·2%)	2690 (34·0%)	3539 (39·4%)	1289 (41·4%)	4951 (36·7%)
	Male	41 025 (56·0%)	21 758 (59·8%)	7001 (58·9%)	11 097 (62·5%)	5199 (65·8%)	5415 (60·3%)	1822 (58·5%)	8504 (63·1%)
	Data missing	195 (0·3%)	88 (0·2%)	22 (0·2%)	43 (0·2%)	12 (0·2%)	19 (0·2%)	4 (0·1%)	31 (0·2%)
Deprivation, IMD quintile†									
	1	10 408 (14·2%)	5201 (14·3%)	1773 (14·9%)	2437 (13·7%)	1152 (14·6%)	1384 (15·4%)	466 (15·0%)	1885 (14·0%)
	2	12 853 (17·6%)	6439 (17·7%)	2147 (18·0%)	2996 (16·9%)	1431 (18·1%)	1634 (18·2%)	552 (17·7%)	2305 (17·1%)
	3	15 822 (21·6%)	7855 (21·6%)	2595 (21·8%)	3793 (21·4%)	1631 (20·6%)	1986 (22·1%)	633 (20·3%)	3035 (22·5%)
	4	16 104 (22·0%)	8069 (22·2%)	2621 (22·0%)	4101 (23·1%)	1748 (22·1%)	2012 (22·4%)	718 (23·0%)	3083 (22·9%)
	5	17 997 (24·6%)	8801 (24·2%)	2759 (23·2%)	4424 (24·9%)	1939 (24·5%)	1956 (21·8%)	745 (23·9%)	3177 (23·6%)
	Data missing	13 (<0·1%)	2 (<0·1%)	0	1 (<0·1%)	0	1 (<0·1%)	1 (<0·1%)	1 (<0·1%)
Race or ethnicity									
	White	53 780 (73·5%)	26 431 (72·7%)	8678 (73·0%)	12 896 (72·6%)	5438 (68·8%)	6624 (73·8%)	2282 (73·3%)	9173 (68·0%)
	South Asian	3318 (4·5%)	1630 (4·5%)	593 (5·0%)	799 (4·5%)	441 (5·6%)	369 (4·1%)	102 (3·3%)	777 (5·8%)
	East Asian	484 (0·7%)	249 (0·7%)	96 (0·8%)	113 (0·6%)	82 (1·0%)	55 (0·6%)	15 (0·5%)	142 (1·1%)
	Black	2480 (3·4%)	1433 (3·9%)	508 (4·3%)	822 (4·6%)	346 (4·4%)	306 (3·4%)	114 (3·7%)	627 (4·6%)
	Other ethnic minority‡	4646 (6·3%)	2435 (6·7%)	751 (6·3%)	1145 (6·4%)	641 (8·1%)	491 (5·5%)	203 (6·5%)	1171 (8·7%)
	Data missing	8489 (11·6%)	4189 (11·5%)	1269 (10·7%)	1977 (11·1%)	953 (12·1%)	1128 (12·6%)	399 (12·8%)	1596 (11·8%)
Diabetes									
	No	49 765 (75·8%)	24 481 (73·6%)	7878 (71·9%)	11 265 (69·7%)	5694 (77·9%)	5948 (72·4%)	2173 (77·7%)	9194 (74·3%)
	Yes	15 855 (24·2%)	8792 (26·4%)	3081 (28·1%)	4891 (30·3%)	1615 (22·1%)	2266 (27·6%)	625 (22·3%)	3173 (25·7%)
Obesity									
	No	53 415 (73·0%)	26 397 (72·6%)	8476 (71·3%)	12 656 (71·3%)	5784 (73·2%)	6331 (70·6%)	2304 (74·0%)	9498 (70·4%)
	Yes	7329 (10·0%)	4230 (11·6%)	1583 (13·3%)	2208 (12·4%)	985 (12·5%)	1226 (13·7%)	296 (9·5%)	2059 (15·3%)
	Data missing	12 453 (17·0%)	5740 (15·8%)	1836 (15·4%)	2888 (16·3%)	1132 (14·3%)	1416 (15·8%)	515 (16·5%)	1929 (14·3%)
Chronic cardiac disease									
	No	45 563 (62·2%)	21 808 (60·0%)	7117 (59·8%)	10 400 (58·6%)	5332 (67·5%)	4077 (45·4%)	1923 (61·7%)	8787 (65·2%)
	Yes	22 563 (30·8%)	12 758 (35·1%)	4235 (35·6%)	6436 (36·3%)	2201 (27·9%)	4496 (50·1%)	995 (31·9%)	4025 (29·8%)
	Data missing	5071 (6·9%)	1801 (5·0%)	543 (4·6%)	916 (5·2%)	368 (4·7%)	400 (4·5%)	197 (6·3%)	674 (5·0%)
Chronic pulmonary disease									
	No	55 604 (76·0%)	27 916 (76·8%)	9261 (77·9%)	13 619 (76·7%)	6404 (81·1%)	6665 (74·3%)	2461 (79·0%)	10 468 (77·6%)
	Yes	12 235 (16·7%)	6472 (17·8%)	2002 (16·8%)	3143 (17·7%)	1100 (13·9%)	1791 (20·0%)	444 (14·3%)	2289 (17·0%)
	Data missing	5358 (7·3%)	1979 (5·4%)	632 (5·3%)	990 (5·6%)	397 (5·0%)	517 (5·8%)	210 (6·7%)	729 (5·4%)
Asthma									
	No	58 352 (79·7%)	29 806 (82·0%)	9782 (82·2%)	14 657 (82·6%)	6525 (82·6%)	7286 (81·2%)	2572 (82·6%)	10 852 (80·5%)
	Yes	9298 (12·7%)	4447 (12·2%)	1482 (12·5%)	2039 (11·5%)	977 (12·4%)	1141 (12·7%)	320 (10·3%)	1849 (13·7%)
	Data missing	5547 (7·6%)	2114 (5·8%)	631 (5·3%)	1056 (5·9%)	399 (5·0%)	546 (6·1%)	223 (7·2%)	785 (5·8%)
Chronic kidney disease									
	No	55 458 (75·8%)	26 793 (73·7%)	8582 (72·1%)	11 962 (67·4%)	6284 (79·5%)	6434 (71·7%)	2368 (76·0%)	10 654 (79·0%)
	Yes	12 182 (16·6%)	7503 (20·6%)	2661 (22·4%)	4785 (27·0%)	1166 (14·8%)	2008 (22·4%)	525 (16·9%)	2070 (15·3%)
	Data missing	5557 (7·6%)	2071 (5·7%)	652 (5·5%)	1005 (5·7%)	451 (5·7%)	531 (5·9%)	222 (7·1%)	762 (5·7%)
Moderate or severe liver disease									
	No	65 646 (89·7%)	33 005 (90·8%)	10 769 (90·5%)	16 111 (90·8%)	6879 (87·1%)	8162 (91·0%)	2764 (88·7%)	12 314 (91·3%)
	Yes	1340 (1·8%)	916 (2·5%)	358 (3·0%)	413 (2·3%)	528 (6·7%)	179 (2·0%)	96 (3·1%)	281 (2·1%)
	Data missing	6211 (8·5%)	2446 (6·7%)	768 (6·5%)	1228 (6·9%)	494 (6·3%)	632 (7·0%)	255 (8·2%)	891 (6·6%)
Mild liver disease									
	No	65 784 (89·9%)	33 164 (91·2%)	10 837 (91·1%)	16 169 (91·1%)	7096 (89·8%)	8178 (91·1%)	2792 (89·6%)	12 338 (91·5%)
	Yes	1035 (1·4%)	635 (1·7%)	240 (2·0%)	294 (1·7%)	269 (3·4%)	132 (1·5%)	60 (1·9%)	222 (1·6%)
	Data missing	6378 (8·7%)	2568 (7·1%)	818 (6·9%)	1289 (7·3%)	536 (6·8%)	663 (7·4%)	263 (8·4%)	926 (6·9%)
Chronic neurological disorder									
	No	58 511 (79·9%)	29 546 (81·2%)	9725 (81·8%)	14 440 (81·3%)	6700 (84·8%)	7357 (82·0%)	2048 (65·7%)	11 352 (84·2%)
	Yes	8802 (12·0%)	4559 (12·5%)	1467 (12·3%)	2167 (12·2%)	729 (9·2%)	1024 (11·4%)	845 (27·1%)	1309 (9·7%)
	Data missing	5884 (8·0%)	2262 (6·2%)	703 (5·9%)	1145 (6·4%)	472 (6·0%)	592 (6·6%)	222 (7·1%)	825 (6·1%)
Malignant neoplasm									
	No	60 050 (82·0%)	29 952 (82·4%)	9485 (79·7%)	14 643 (82·5%)	6620 (83·8%)	7378 (82·2%)	2564 (82·3%)	11 283 (83·7%)
	Yes	7072 (9·7%)	4075 (11·2%)	1675 (14·1%)	1932 (10·9%)	819 (10·4%)	994 (11·1%)	307 (9·9%)	1341 (9·9%)
	Data missing	6075 (8·3%)	2340 (6·4%)	735 (6·2%)	1177 (6·6%)	462 (5·8%)	601 (6·7%)	244 (7·8%)	862 (6·4%)
Chronic haematological disease									
	No	64 082 (87·5%)	32 079 (88·2%)	10 150 (85·3%)	15 622 (88·0%)	6958 (88·1%)	7906 (88·1%)	2737 (87·9%)	12 003 (89·0%)
	Yes	2982 (4·1%)	1907 (5·2%)	1017 (8·5%)	942 (5·3%)	447 (5·7%)	461 (5·15)	122 (3·9%)	600 (4·4%)
	Data missing	6133 (8·4%)	2381 (6·5%)	728 (6·1%)	1188 (6·7%)	496 (6·3%)	606 (6·8%)	256 (8·2%)	883 (6·5%)
HIV/AIDs									
	No	65 920 (90·1%)	33 268 (91·5%)	10 828 (91·0%)	16 190 (91·2%)	7256 (91·8%)	8195 (91·3%)	2809 (90·2%)	12 360 (91·7%)
	Yes	256 (0·3%)	149 (0·4%)	57 (0·5%)	82 (0·5%)	42 (0·5%)	28 (0·3%)	13 (0·4%)	57 (0·4%)
	Data missing	7021 (9·6%)	2950 (8·1%)	1010 (8·5%)	1480 (8·3%)	603 (7·6%)	750 (8·4%)	293 (9·4%)	1069 (7·9%)
Rheumatological disorder									
	No	59 168 (80·8%)	29 823 (82·0%)	9663 (81·2%)	14 540 (81·9%)	6708 (84·9%)	7294 (81·3%)	2512 (80·6%)	11 245 (83·4%)
	Yes	7724 (10·6%)	4075 (11·2%)	1462 (12·3%)	1961 (11·0%)	701 (8·9%)	1061 (11·8%)	353 (11·3%)	1358 (10·1%)
	Data missing	6305 (8·6%)	2469 (6·8%)	770 (6·5%)	1251 (7·0%)	492 (6·2%)	618 (6·9%)	250 (8·0%)	883 (6·5%)
Dementia									
	No	55 758 (76·2%)	28 473 (78·3%)	9548 (80·3%)	13 583 (76·5%)	6708 (84·9%)	7079 (78·9%)	2237 (71·8%)	11 449 (84·9%)
	Yes	11 682 (16·0%)	5668 (15·6%)	1624 (13·7%)	3064 (17·3%)	750 (9·5%)	1306 (14·6%)	645 (20·7%)	1239 (9·2%)
	Data missing	5757 (7·9%)	2226 (6·1%)	723 (6·1%)	1105 (6·2%)	443 (5·6%)	588 (6·6%)	233 (7·5%)	798 (5·9%)
Smoking									
	Never smoked	23 944 (32·7%)	11 976 (32·9%)	4071 (34·2%)	5577 (31·4%)	2811 (35·6%)	2872 (32·0%)	889 (28·5%)	4894 (36·3%)
	Current smoker	3895 (5·3%)	1927 (5·3%)	677 (5·7%)	875 (4·9%)	508 (6·4%)	459 (5·1%)	188 (6·0%)	694 (5·1%)
	Former smoker	15 834 (21·6%)	8533 (23·5%)	2914 (24·5%)	4179 (23·5%)	1740 (22·0%)	2317 (25·8%)	630 (20·2%)	3304 (24·5%)
	Data missing	29 524 (40·3%)	13 931 (38·3%)	4233 (35·6%)	7121 (40·1%)	2842 (36·0%)	3325 (37·1%)	1408 (45·2%)	4594 (34·1%)

Data are n or n (%). No means patients didn't have the comorbidity or characteristic, yes means they did. IMD=Index of Multiple Deprivation.

* Severe acute respiratory infection was contained within case definition so was not counted as a complication.

† 1=least deprived, 5=most deprived.

‡ Includes West Asian, Latinx, Aboriginal, and First Nations People.

The mean age of patients included in our study was 71·1 years old (SD 18·7), with the majority of those included being male (table 1). One or more comorbidities were present in 81·0% (59 289 of 73 197) of the cohort. Chronic cardiac disease was the most common comorbidity, followed by chronic pulmonary disease and chronic kidney disease. Most of the study cohort consisted of White people.

In adult patients with COVID-19, renal, complex respiratory, cardiovascular, neurological, gastrointestinal, and systemic complications were reported (table 1). Specific complications within each organ system were also reported, with acute kidney injury, probable ARDS, liver injury, anaemia, and cardiac arrhythmia being the most common (appendix pp 4–5). The incidence of acute kidney injury increased with age and was most common in patients aged between 60 and 90 years, with males at greater risk. Patients with chronic kidney disease were at the highest risk of acute kidney injury, with 39·8% (4785 of 12 182) developing acute kidney injury versus 21·6% (11 962 of 55 458) in patients without chronic kidney disease. Cardiac complications were more frequently observed with increasing age and in patients with existing cardiac disease. In those with existing cardiac disease, 19·9% (4496 of 22 563) developed a cardiac complication compared with 8·9% (4077 of 45 563) in those without previous cardiac disease. In contrast, liver injury was most frequently seen in younger age groups (aged <60 years), with the highest rates occurring in males. Liver injury was more common in patients with pre-existing moderate or severe liver disease (300 [22·4%] of 1340) compared with those without liver injury (4097 [6·2%] of 65 646). Complication rates were comparable across White, South Asian, and East Asian ethnic and racial groups, but were highest in Black people (57·8% [1433 of 2480] in Black patients *vs* 49·1% [26 431 of 53 780] in White patients; table 1). Rates of acute kidney injury were highest in Black patients (822 [33·1%] of 2480) compared with White patients (12 896 [24·0%] of 53 780). Patients with obesity were 1·6 times more likely to have respiratory complications (2059 [28·1%] of 7329) compared with those who did not have obesity (9498 [17·8%] of 53 415; table 1). Patients who had obesity were also 1·3 times more likely to have renal complications (2208 [30·1%] of 7329) compared with those who did not have obesity (12 656 [23·7%] of 53 415; table 1).

Suspected bacterial pneumonia was the most common respiratory complication (appendix pp 6–7), but when the definition incorporated positive microbiological testing (highly likely bacterial pneumonia), the incidence of highly likely bacterial pneumonia was lower. Acute kidney injury (Jaccard index 0·23), likely ARDS (Jaccard index 0·17), anaemia (Jaccard index 0·13), and liver injury (Jaccard index 0·10) were most likely to co-occur with death (appendix p 35).

**Figure 2:**
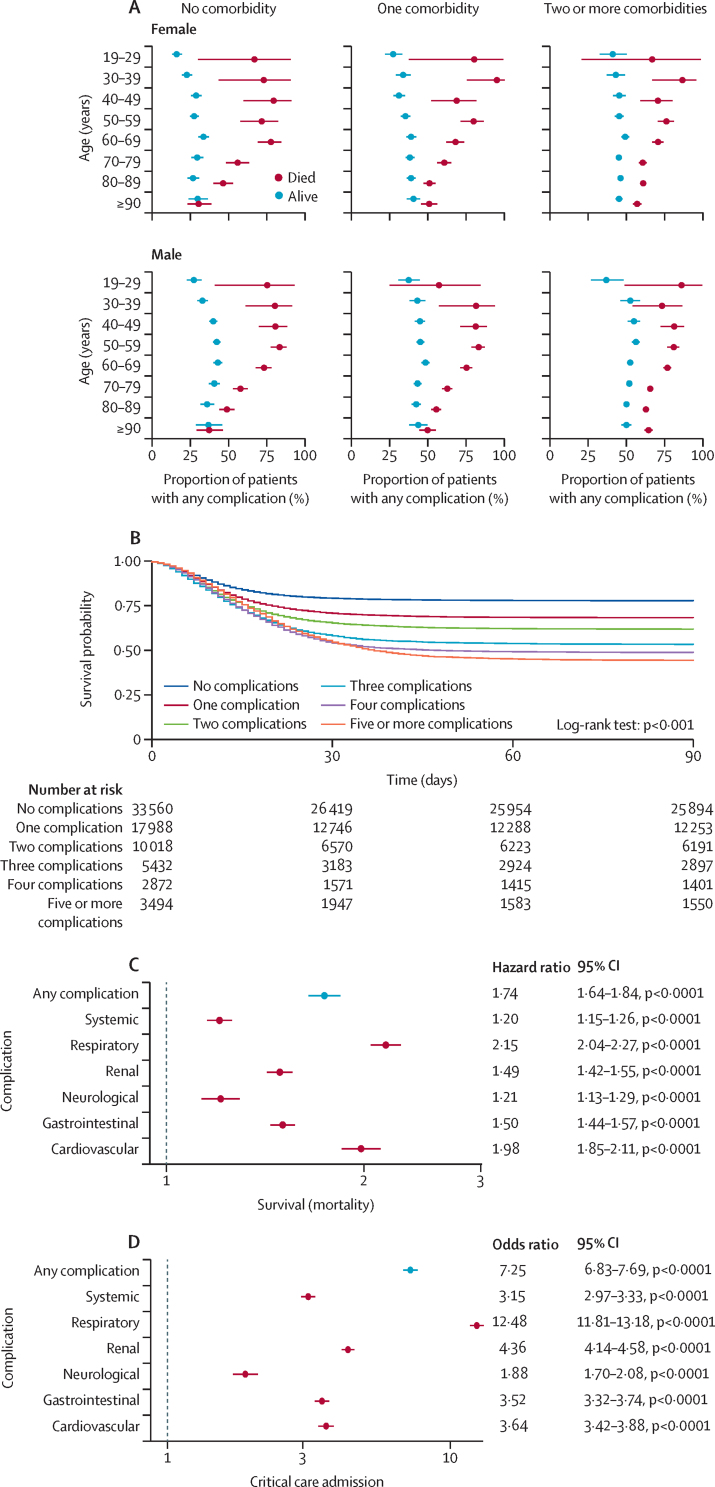
Outcomes and mortality after complications (A) Differences in complication rates, age, sex, and comorbidity. (B) Kaplan-Meier survival curve stratified by number of complications had. The hazard ratios are: no complications 1 (reference level); one complication 1·50 (95% CI 1·45–1·55, p<0·0001); two complications 1·87 (1·80–1·94, p<0·0001); three complications 2·39 (2·29–2·50, p<0·0001); four complications 2·64 (2·50–2·79, p<0·0001); and five complications 2·81 (2·67–2·95, p< 0·0001). (C) Hazard ratios for effect of organ-specific complications on overall survival, adjusted for age, sex, indices of multiple deprivation quintile, and study centre (appendix pp 14–20). (D) Effect of organ-specific complications on odds of being admitted to critical care (appendix pp 21–27). Error bars represent 95% CIs.

Having at least one complication was common across all demographic groups, with the lowest rates in patients aged 19–29 years with no comorbidity (178 [21·2%] of 839) and the highest rates in patients aged 60–69 years who had two or more comorbidities (3340 [57·9%] of 5767; appendix pp 8–11). The incidence of complications rose with increasing age occurring in 38·9% (3596 of 9249) in those aged 19–49 years and 51·3% (32 771 of 63 948) in those aged 50 years and older (figure 2A). The number of complications increased with the number of pre-existing comorbidities, particularly in individuals aged 40 years and older (figure 2A and appendix pp 8–11). Complications were higher in males compared with females, and males were more likely to have complications than females, with males aged older than 60 years the most likely group to have at least one complication (aged <60 years: 36·6% [2814 of 7689] in females and 48·8% [5179 of 10 609] in males; aged ≥60 years: 48·2% [11 707 of 24 288] in females and 54·5% [16 579 of 30 416] in males; figure 2A and appendix pp 4–5). Young males (aged 19–29 years) without comorbidities were significantly more likely to have complications than young females (aged 19–29 years) without comorbidities (28·4% [94 of 331] in males and 16·6% [84 of 505] in females; figure 2A). When we stratified by mortality, complications occurred more frequently in patients who died (14 583 [63·2%] of 23 092), but were still common in survivors (21 784 [43·5%] of 50 105; appendix pp 12–13) and there were direct relationships between worse survival and increasing numbers of complications (figure 2B).

**Figure 3:**
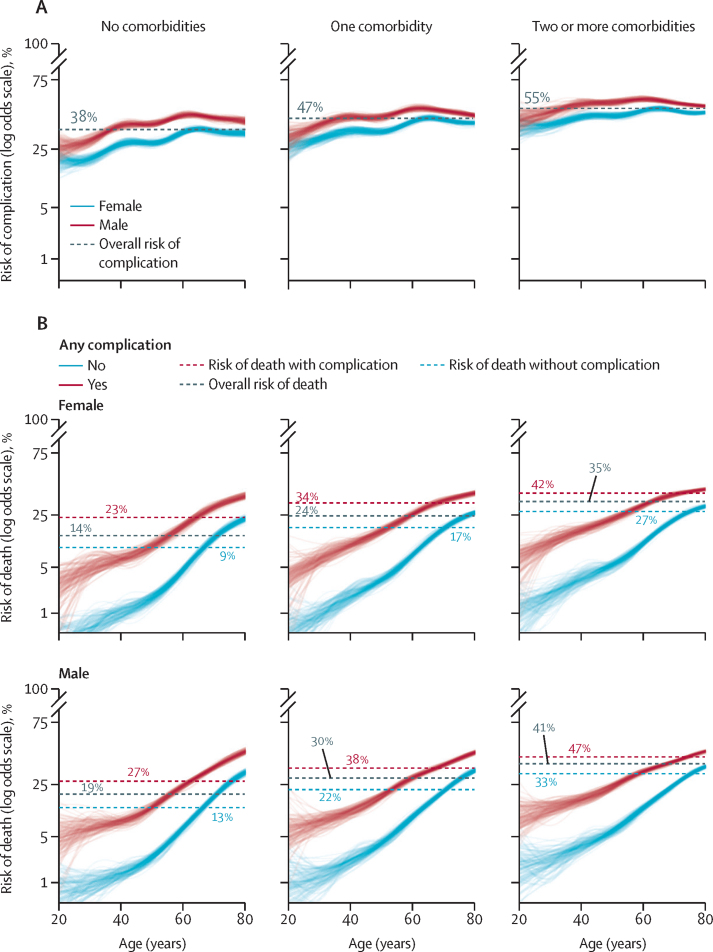
Relationship between age, sex, comorbidities, and adjusted outcomes using generalised additive models (A) Relationships for the outcome of adjusted risk of any complication. (B) Relationships for the outcome of adjusted mortality risk, stratified by presence of complications. Each line represents one bootstrap replicate (ie, one simulated patient). The appendix (pp 39–44) shows models for other organ-specific complications.

After adjusting for age, sex, deprivation, comorbidities, and study centre, increasing age and male sex were significant independent predictors for developing any complication and for all organ-specific complications except for gastrointestinal and liver complications, which younger patients were more likely to experience (figure 3A and appendix pp 36–44). Those with pre-existing comorbidities that affected a specific organ system were at higher risk of developing a complication affecting the same organ (appendix pp 45–46). The relationship between increasing age, male sex, and the risk of complications persisted independent of the number of comorbidities (figure 3A and appendix pp 39–44). The risk of complications and relationship between age and risk of complications were comparable across all comorbidity groups.

In patients who survived to 28 days from first symptoms to discharge, 44·9% (23 619 of 52 582) suffered complications, compared with 61·9% (12 624 of 20 384) in those who died within 28 days. Complications were more common in those requiring respiratory support and were highest in patients who received critical care (8267 [82·4%] of 10 034) or invasive mechanical ventilation (5619 [91·7%] of 6122; table 2). The presence and number of complications was significantly associated with worse in-hospital survival (figure 2B). Following adjustment for age, sex, deprivation, and hospital, the occurrence of any complication was significantly associated with poorer overall survival (figure 2C). Cardiovascular (hazard ratio 1·98, 95% CI 1·85–2·11) and complex respiratory complications (2·15, 2·04–2·27) were most strongly associated with worse outcomes. After adjusting for age, sex, and deprivation, patients having an acute kidney injury were 4 times more likely to be admitted to critical care, and those with respiratory complications were 13 times more likely to be admitted to critical care (figure 2D).

**Table 2:** Outcomes by organ-specific complications

		**Patients having complications**		**Organ-specific complications**					
		Total number of patients	Any complication	Systemic	Renal	Gastrointestinal (including liver)	Cardiovascular	Neurological	Respiratory*
Total number of patients		73 197	36 367 (49·7%)	11 895 (16·3%)	17 752 (24·3%)	7901 (10·8%)	8973 (12·3%)	3115 (4·3%)	13 486 (18·4%)
Death									
	No	50 105 (68·5%)	21 784 (59·9%)	7423 (62·4%)	10 059 (56·7%)	4837 (61·2%)	4035 (45·0%)	1880 (60·4%)	7028 (52·1%)
	Yes	23 092 (31·5%)	14 583 (40·1%)	4472 (37·6%)	7693 (43·3%)	3064 (38·8%)	4938 (55·0%)	1235 (39·6%)	6458 (47·9%)
Critical care admission									
	No	62 125 (84·9%)	28 092 (77·2%)	8804 (74·0%)	12 992 (73·2%)	5139 (65·0%)	6640 (74·0%)	2446 (78·5%)	7472 (55·4%)
	Yes	10 034 (13·7%)	8267 (22·7%)	3090 (26·0%)	4755 (26·8%)	2760 (34·9%)	2333 (26·0%)	668 (21·4%)	6012 (44·6%)
	Data missing	1038 (1·4%)	8 (<0·1%)	1 (<0·1%)	5 (<0·1%)	2 (<0·1%)	0	1 (<0·1%)	2 (<0·1%)
Any invasive ventilation									
	No	65 888 (90·0%)	30 710 (84·4%)	9556 (80·3%)	14 262 (80·3%)	5815 (73·6%)	7186 (80·1%)	2573 (82·6%)	8809 (65·3%)
	Yes	6122 (8·4%)	5619 (15·5%)	2330 (19·6%)	3471 (19·6%)	2077 (26·3%)	1784 (19·9%)	542 (17·4%)	4670 (34·6%)
	Data missing	1187 (1·6%)	38 (0·1%)	9 (0·1%)	19 (0·1%)	9 (0·1%)	3 (<0·1%)	0	7 (0·1%)
Any non-invasive ventilation									
	No	60 035 (84·7%)	28 202 (78·5%)	9228 (78·2%)	13 361 (76·1%)	5685 (72·8%)	6862 (77·1%)	2566 (83·3%)	8332 (62·3%)
	Yes	10 827 (15·3%)	7741 (21·5%)	2567 (21·8%)	4194 (23·9%)	2124 (27·2%)	2034 (22·9%)	513 (16·7%)	5038 (37·7%)
Any oxygen									
	No	17 652 (24·7%)	5971 (16·5%)	2079 (17·6%)	2470 (14·0%)	1153 (14·7%)	1190 (13·3%)	737 (23·8%)	838 (6·2%)
	Yes	53 695 (75·3%)	30 181 (83·5%)	9762 (82·4%)	15 189 (86·0%)	6705 (85·3%)	7744 (86·7%)	2358 (76·2%)	12 598 (93·8%)

Data are n or n (%). No means patients did not have the clinical outcome specified in the table rows, yes means they did.

* Severe acute respiratory infection was contained within case definition so was not counted as a complication.

When the relationships between complications and mortality were modelled using generalised additive models and plotted (figure 3B and appendix pp 39–44), the presence of any complication, in addition to increasing age and male sex, was associated with death. In younger people, the presence of a complication was associated with a large increase in the risk of mortality, compared with older people, in which the presence of a complication was associated with a much smaller increase in mortality. Associations between complications and mortality were similar across comorbidity groups overall, but we identified that in younger people with comorbidities, mortality was much higher in those who had complications compared with people of the same age without complications. Respiratory and cardiovascular complications were associated with the largest increases in death across all ages, whereas those with neurological or systemic complications were most likely to survive (appendix pp 39–44).

Physiology-based early warning scores and the 4C Mortality Score, calculated using parameters at hospital admission, were associated with the occurrence of complications in survivors. Higher 4C Mortality Score on admission corresponded with an increased probability of at least one complication (appendix p 47). Similarly, higher NEWS2 and qSOFA scores on admission were associated with an increased probability of one or more complications (appendix p 47). The number of symptoms on admission did not appear to be related to the incidence of complications (appendix p 47).

**Figure 4:**
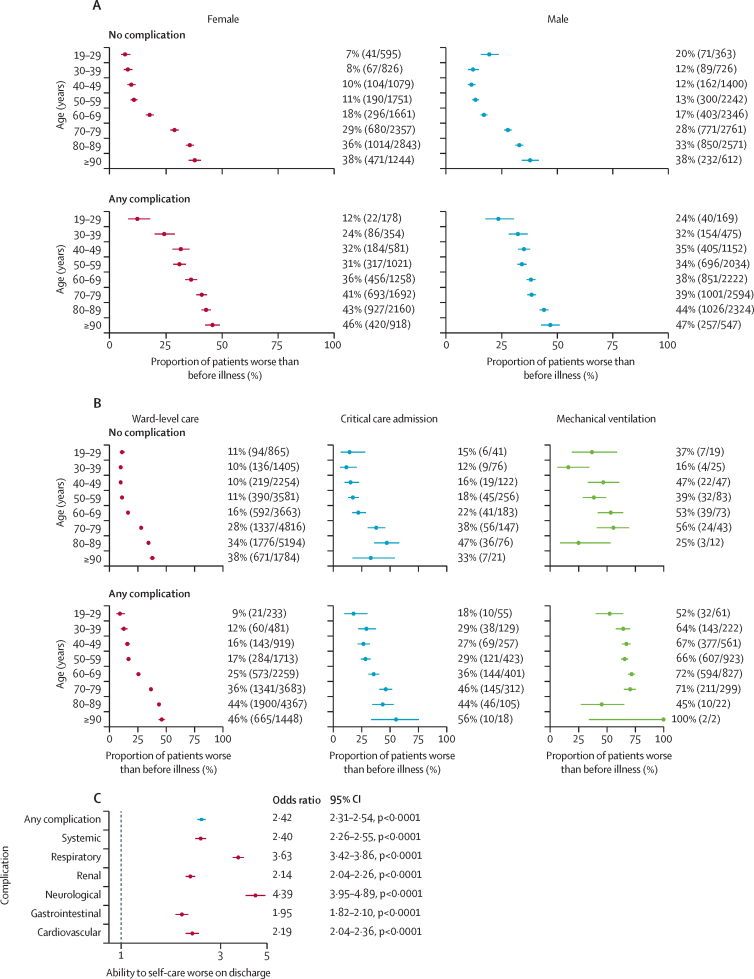
Relationship between in-hospital complications and ability to self-care at time of discharge or transfer to other health-care facility (A) Ability to self-care at discharge in patients who had complications by age group and sex. (B) Ability to self-care at discharge by disease severity. (C) Adjusted odds of worse ability to self-care at discharge by organ-specific complications in adults admitted to hospital with severe COVID-19 (appendix pp 27–34). Error bars represent 95% CIs.

In those who survived, 26·6% (13 309 of 50 105) of patients had worse ability to self-care than they did before their illness (figure 4A). This worsening of ability increased with age, male sex, and in those who received critical care support (figures 4A, B). Having a complication was independently associated with an increased risk of worse ability to self-care after discharge after adjusting for age, sex, deprivation, and hospital (adjusted odds ratio 2·42, 95% CI 2·31–2·54; figure 4C). Neurological complications had the strongest associations with worse functional outcome (4·39, 3·95–4·63; figure 4C).

## Discussion

Hospitalisation with COVID-19 is associated with high rates of morbidity in adults. Almost half of the survivors had one or more complications, which were more likely in patients who required critical care. Survivors of COVID-19 who had suffered at least one complication had a lower ability to self-care on discharge from hospital. The effect of complications on the ability to self-care was most profound in younger patients (aged <50 years). We found that complication rates were high in every age group and increased with age. Unlike mortality, there were only small differences in complication rates in groups stratified by pre-existing comorbidity. Males were significantly more likely to develop complications than females.

The most common complications in our data were acute kidney injury, and complex respiratory and systemic complications. Although our study only looked at complications during the first admission for COVID-19, many of the common complications identified are associated with substantial long-term morbidity. Acute kidney injury is known to be associated with increased long-term hazards of mortality, requirement for dialysis, and an increase in cardiovascular events.^19^, ^20^, ^21^ In addition to the more common complications identified, rarer complications including stroke, congestive heart failure, and cardiac arrest were present in 1–5% of patients.^22^, ^23^, ^24^ Patients who received critical care had the highest complication rates, compatible with previous observations describing high levels of morbidity in those who require critical care.^6^, ^8^, ^25^, ^26^ The least commonly observed were neurological complications, although these were the most strongly associated with reduced ability to self-care. Suspected bacterial pneumonia and likely ARDS were the most common respiratory complications. When compared with the published literature on influenza, complications rates in patients with COVID-19 were the same or higher.^27^, ^28^, ^29^ Notably, this higher rate of complications appears to be primarily driven by non-infectious complications, as the rates of secondary bacterial infection in patients with COVID-19 were lower than described in influenza.^30^ In particular, COVID-19 patients had up to 19 times the risk of developing likely ARDS when compared with patients admitted with influenza.^31^

Most clinical studies of COVID-19 have focused on associated mortality.^1^ Mortality is a hard endpoint, easily measured, and of utmost importance. However, its use as a sole outcome in COVID-19 studies might underestimate the detrimental impact of COVID-19, particularly in those who are younger or otherwise healthy. Our analysis suggests that the odds of some complications change little with increasing age in those older than 50 years. Therefore, when compared with mortality, complications will affect many more people across a range of different age groups. Notably, our data show only small increases in the risk of complications by pre-existing comorbidities. The effect of comorbidities on the risk of complications and death was substantially higher in younger people compared with people without comorbidities of the same age. We also observed the differences in number of complications decrease between those who died and those who survived as age increased, suggesting that although young people are less likely to die, they might be proportionally more likely to survive and live with complications. Patients with complications are also likely to have impaired ability to self-care following discharge from hospital. This finding contradicts current narratives that COVID-19 is only dangerous in people with existing comorbidities and the elderly. Dispelling and contributing to the scientific debate around such narratives has become increasingly important. Many countries including the UK are experiencing further waves of infection.^32^ Suggestions have been made around using younger, healthy demographic groups who are less likely to die, to help support economic output, and to propagate herd immunity within a population.^33^ Policy makers need to consider not just mortality when making decisions around easing population-level interventions designed to limit spread, but also the risk of both short-term and long-term complications for those who survive COVID-19.

Our data provide the most comprehensive, multicentre, systematic analysis of the effect of COVID-19 on short-term clinical outcomes in a hospitalised population, including patient groups from both ward level and critical care. Data were collected prospectively and capture most people hospitalised with COVID-19 in the UK. Recruitment to our study continues, enabling us to capture trends and incidence of complications in near real time. Other smaller, or single centre studies, have typically focused either exclusively on patients who received critical care, or on one type of complication and lack systematic approaches to data collection.^4^, ^34^, ^35^, ^36^, ^37^, ^38^ Our study identifies high rates of complications and the risk factors for developing these, and describes severity, which previous studies have been unable to do at scale. In particular, we find that in the short term, respiratory and cardiovascular complications have the strongest association with mortality. A further strength is that our study includes patients in both critical care and in ward-level areas, whereas other groups have just studied intensive care populations.^39^ In addition, the multicentre nature of our study across 302 facilities in four countries increases the generalisability of our findings, which is particularly important to provide robust estimates of short-term morbidity for health-care planners and policy makers. The large sample size of our study allowed us to do meaningful subgroup analyses and integrate blood test and microbiology results to increase robustness. This size also meant we could detect rare events in important patient groups such as those receiving critical care, younger patients, and survivors where complications might have the biggest effect and be with patients for a long period of time after the initial event.

This study has important implications for clinicians. It was not possible for us to causally link complications and consequent poor outcomes. However, it is plausible that interventions targeted at preventing in-hospital complications or reducing their impact could plausibly improve outcomes. We found respiratory and cardiovascular complications were associated with greatest severity and acute kidney injury was one of the most common. Treatments such as enhanced monitoring and early treatment for patients for cardiac arrhythmias that might lead to further problems such as stroke or cardiac arrest might, therefore, be useful. Similarly, for acute kidney injury, optimising fluid balance to ensure adequate renal perfusion in patients with less severe respiratory disease might lessen the impact of acute kidney injury. Our data also present research opportunities for preventing complications that contribute to substantial disability. For example, further characterisation of thromboembolic complications and stroke can help to identify optimal anticoagulation strategies in patients with COVID-19.^40^ We found initial disease severity, measured using the 4C Mortality Score, qSOFA, and NEWS, were associated with the presence of complications, and could therefore be useful tools to stratify those at the highest risk of developing complications in clinical practice and interventional trials.

There are several limitations to our study, which relate to the design and current unknowns in COVID-19 research. First, this dataset focuses on in-hospital complications during the index admission for COVID-19 and does not provide longer-term outcome data or data on quality of life. Nevertheless, our results suggest that complications of COVID-19 might affect all survivor groups, rather than just those who are older and have comorbidities. Second, the complications that were captured were predefined by a pragmatic outbreak preparedness study protocol, and case report forms developed for disease X, long before the emergence of SARS-CoV-2. The outcomes we chose are both clinically important and associated with complications observed in other infectious viral diseases. Local investigators could enter other complications as free text, but this approach might have missed some important outcomes that were otherwise unexpected (ie, venous thromboembolism); however, as these emerged we amended the case report form to include these. This suggests that our estimates are likely to be conservative, when compared with the incidence of some complications (including pulmonary embolism or deep vein thrombosis) found in other smaller studies. Similarly, these studies are more likely to focus on populations with higher COVID-19 severity, where our study captured all hospital admissions.^41^ This protocol did not include a non-SARS-CoV-2 comparator group, which could provide useful data to compare complication burdens to other causes of critical illness or viral infection. Third, owing to logistical constraints, we did not capture data on the timings of each complication. As our study was an urgent response to the emerging pandemic, it would not have been possible to identify exactly when each complication started for such a large number of patients. Data around timings could in the future help to identify sequences of events that lead to further deterioration. Fourth, our data can only provide estimates of who gets complications in a hospitalised population. We found that even in previously healthy adults with no recorded comorbidity, complications affected more than four in ten hospitalised patients; the effect and burden in the community remains undescribed. For infection-related outcomes, we systematically classified microbiological culture results to identify whether infections were caused by pathogenic organisms. However, individuals might have acquired these in the community, so our estimates encompass both hospital and community acquired infection. In addition to this, the UK health service was under considerable pressure, which could have resulted in preferential admission to hospital of patients with the most severe disease. This might lead to an increase in the observed complication rate, as individuals with milder disease were managed at home. However, the risk of this is reduced by the multicentre design of our study, as peaks in hospital admissions varied in the UK over time. Compared with other international cohorts, our study had a higher observed hospital case fatality rate.^42^, ^43^, ^44^, ^45^ The reasons for this are multifactorial, and could relate to differences in testing strategy, thresholds for hospital admission, pre-existing population morbidity, and health-care system preparedness. Finally, our data were collected from real-world observed clinical practice and patients did not undergo any additional tests to detect the presence of complications. Therefore, the true burden of complications is likely to be higher. However, doing large numbers of invasive tests might not be acceptable for patients, particularly in patients who are unlikely to survive or cannot tolerate investigations, and would be logistically challenging in a study of this size.

Policy makers and health-care planners should anticipate that large amounts of health and social care resources will be required to support those who survive COVID-19. This includes adequate provision of staffing and equipment; for example, provision of follow-up clinics for those who have sustained in-hospital complications such as acute kidney injury or respiratory tract infection. Beyond the short term, further work is underway to establish the consequences of these complications and whether these are transient or linked to worse long-term outcomes. Data on long-term health difficulties posed by COVID-19 will be of great importance, particularly as a large proportion of COVID-19 survivors come from economically active age groups. This should be considered on a policy level in terms of return to work and education; but importantly, it could have effects on individual behaviour around perceived benefits of engaging with preventive measures including vaccination.

In summary, high rates of complications and poor functional outcomes were present in survivors of COVID-19, including in young and previously healthy individuals. Those aged older than 50 years and admitted to critical care were at the highest risk. Common COVID-19 complications identified in this Article are known to be associated with long-term morbidity and an increased risk of death.

## Data sharing

Data, protocols, and all documentation around this analysis will be made available to academic researchers after authorisation from the independent data access and sharing committee. Data and analysis scripts are available on request to the Independent Data Management and Access Committee at https://isaric4c.net/ sample_access.

## Declaration of interests

ABD reports grants from the Department of Health and Social Care (DHSC), during the conduct of the study; and grants from Wellcome Trust, outside the submitted work. PJMO reports institutional fees from consultancies from Janssen, Oxford Immunotech, Nestle, Pfizer, and the European Respiratory Society; grants from the MRC, MRC Global Challenge Research Fund, EU, NIHR Biomedical Research Centre, MRC, GlaxoSmithKline, Wellcome Trust, and NIHR Health Protection Research Unit (HPRU) in Respiratory Infection; and is NIHR senior investigator outside the submitted work. PJMO's role as president of the British Society for Immunology was unpaid but travel and accommodation at some meetings was provided by the Society.JKB reports grants from MRC UK. MGS reports grants from DHSC, NIHR UK, MRC UK, HPRU in Emerging and Zoonotic Infections, and University of Liverpool, during the conduct of the study; and is chair of the Infectious Diseases Science Advisory Board and minority shareholder of Integrum Scientific, Greensboro NC, outside the submitted work. All other authors declare no competing interests.

## References

[1] Docherty AB, Harrison EM, Green CA (2020). Features of 20 133 UK patients in hospital with COVID-19 using the ISARIC WHO Clinical Characterisation Protocol: prospective observational cohort study. BMJ.

[2] Carfì A, Bernabei R, Landi F (2020). Persistent symptoms in patients after acute COVID-19. JAMA.

[3] Paterson RW, Brown RL, Benjamin L (2020). The emerging spectrum of COVID-19 neurology: clinical, radiological and laboratory findings. Brain.

[4] Puntmann VO, Carerj ML, Wieters I (2020). Outcomes of cardiovascular magnetic resonance imaging in patients recently recovered from coronavirus disease 2019 (COVID-19). JAMA Cardiol.

[5] Needham DM, Feldman DR, Kho ME (2011). The functional costs of ICU survivorship. Collaborating to improve post-ICU disability. Am J Respir Crit Care Med.

[6] Lone NI, Gillies MA, Haddow C (2016). Five-year mortality and hospital costs associated with surviving intensive care. Am J Respir Crit Care Med.

[7] Nepogodiev D, Bhangu A, Glasbey JC (2020). Mortality and pulmonary complications in patients undergoing surgery with perioperative SARS-CoV-2 infection: an international cohort study. Lancet.

[8] Docherty AB, Sim M, Oliveira J (2017). Early troponin I in critical illness and its association with hospital mortality: a cohort study. Crit Care.

[9] Chapman AR, Shah ASV, Lee KK (2018). Long-term outcomes in patients with type 2 myocardial infarction and myocardial injury. Circulation.

[10] Heyland DK, Groll D, Caeser M (2005). Survivors of acute respiratory distress syndrome: relationship between pulmonary dysfunction and long-term health-related quality of life. Crit Care Med.

[11] Warren-Gash C, Blackburn R, Whitaker H, McMenamin J, Hayward AC (2018). Laboratory-confirmed respiratory infections as triggers for acute myocardial infarction and stroke: a self-controlled case series analysis of national linked datasets from Scotland. Eur Respir J.

[12] Dunning JW, Merson L, Rohde GGU (2014). Open source clinical science for emerging infections. Lancet Infect Dis.

[13] WHO (2020). Multisystem inflammatory syndrome in children and adolescents temporally related to COVID-19. https://www.who.int/news-room/commentaries/detail/multisystem-inflammatory-syndrome-in-children-and-adolescents-with-covid-19.

[14] ISARIC 4C (Coronavirus Clinical Characterisation Consortium) Site set-up. https://isaric4c.net/protocols/.

[15] Von Elm E, Altman DG, Egger M, Pocock SJ, Gøtzsche PC, Vandenbroucke JP (2007). The Strengthening the Reporting of Observational Studies in Epidemiology (STROBE) statement: guidelines for reporting observational studies. PLoS Med.

[16] Harris PA, Taylor R, Thielke R, Payne J, Gonzalez N, Conde JG (2009). Research electronic data capture (REDCap)–a metadata-driven methodology and workflow process for providing translational research informatics support. J Biomed Inform.

[17] Kellum JA, Lameire N, Aspelin N (2012). Kidney Disease: Improving Global Outcomes (KDIGO) acute kidney injury work group. KDIGO clinical practice guideline for acute kidney injury. Kidney International Supplements.

[18] Knight SR, Ho A, Pius R (2020). Risk stratification of patients admitted to hospital with covid-19 using the ISARIC WHO Clinical Characterisation Protocol: development and validation of the 4C Mortality Score. BMJ.

[19] Doyle JF, Forni LG (2016). Acute kidney injury: short-term and long-term effects. Crit Care.

[20] Odutayo A, Wong CX, Farkouh M (2017). AKI and long-term risk for cardiovascular events and mortality. J Am Soc Nephrol.

[21] Parikh CR, Coca SG, Wang Y, Masoudi FA, Krumholz HM (2008). Long-term prognosis of acute kidney injury after acute myocardial infarction. Arch Intern Med.

[22] James SL, Abate D, Abate KH (2018). Global, regional, and national incidence, prevalence, and years lived with disability for 354 diseases and injuries for 195 countries and territories, 1990–2017: a systematic analysis for the Global Burden of Disease Study 2017. Lancet.

[23] Feigin VL, Nichols E, Alam T (2019). Global, regional, and national burden of neurological disorders, 1990–2016: a systematic analysis for the Global Burden of Disease Study 2016. Lancet Neurol.

[24] Phelps R, Dumas F, Maynard C, Silver J, Rea T (2013). Cerebral performance category and long-term prognosis following out-of-hospital cardiac arrest. Crit Care Med.

[25] Adhikari NKJ, Fowler RA, Bhagwanjee S, Rubenfeld GD (2010). Critical care and the global burden of critical illness in adults. Lancet.

[26] Girling BJ, Channon SW, Haines RW, Prowle JR (2019). Acute kidney injury and adverse outcomes of critical illness: correlation or causation?. Clin Kidney J.

[27] Martin-Loeches I, Papiol E, Rodríguez A (2011). Acute kidney injury in critical ill patients affected by influenza A (H1N1) virus infection. Crit Care.

[28] Papic N, Pangercic A, Vargovic M, Barsic B, Vince A, Kuzman I (2012). Liver involvement during influenza infection: perspective on the 2009 influenza pandemic. Influenza Other Respir Viruses.

[29] Kalil AC, Thomas PG (2019). Influenza virus-related critical illness: pathophysiology and epidemiology. Crit Care.

[30] Klein EY, Monteforte B, Gupta A (2016). The frequency of influenza and bacterial coinfection: a systematic review and meta-analysis. Influenza Other Respir Viruses.

[31] Cates J, Lucero-Obusan C, Dahl RM (2020). Risk for in-hospital complications associated with covid-19 and influenza - veterans health administration, United States, October 1, 2018–May 31, 2020. MMWR Morb Mortal Wkly Rep.

[32] Public Health England (2020). Weekly coronavirus disease 2019 (COVID-19) surveillance report—summary of COVID-19 surveillance systems. https://www.gov.uk/government/publications/national-covid-19-surveillance-reports.

[33] Sky News (2020). Coronavirus: top scientists call for herd immunity approach—as government's ‘soft touch’ criticised. https://news.sky.com/story/scientists-and-politicians-split-over-how-to-tackle-rising-covid-infections-as-northern-leaders-say-restrictions-are-not-working-12096597.

[34] Hendren NS, Drazner MH, Bozkurt B, Cooper LT (2020). Description and proposed management of the acute COVID-19 cardiovascular syndrome. Circulation.

[35] Alqahtani SA, Schattenberg JM (2020). Liver injury in COVID-19: the current evidence. United European Gastroenterol J.

[36] El Moheb M, Naar L, Christensen MA (2020). Gastrointestinal complications in critically ill patients with and without COVID-19. JAMA.

[37] Zhou F, Yu T, Du R (2020). Clinical course and risk factors for mortality of adult inpatients with COVID-19 in Wuhan, China: a retrospective cohort study. Lancet.

[38] Casas-Aparicio GA, León-Rodríguez I, Alvarado-de CLB (2021). Acute kidney injury in patients with severe COVID-19 in Mexico. PLoS One.

[39] Intensive Care National Audit & Research Centre (2021). COVID-19 reports. https://www.icnarc.org/Our-Audit/Audits/Cmp/Reports.

[40] Llitjos JF, Leclerc M, Chochois C (2020). High incidence of venous thromboembolic events in anticoagulated severe COVID-19 patients. J Thromb Haemost.

[41] Khan MS, Shahid I, Anker SD (2020). Cardiovascular implications of COVID-19 versus influenza infection: a review. BMC Med.

[42] Stafford N (2020). Covid-19: why Germany's case fatality rate seems so low. BMJ.

[43] Nachtigall I, Lenga P, Jóźwiak K (2020). Clinical course and factors associated with outcomes among 1904 patients hospitalized with COVID-19 in Germany: an observational study. Clin Microbiol Infect.

[44] Bellan M, Patti G, Hayden E (2020). Fatality rate and predictors of mortality in an Italian cohort of hospitalized COVID-19 patients. Sci Rep.

[45] Piroth L, Cottenet J, Mariet AS (2021). Comparison of the characteristics, morbidity, and mortality of COVID-19 and seasonal influenza: a nationwide, population-based retrospective cohort study. Lancet Respir Med.

