
JOURNAL INFORMATION
==============================
NLM Title Abbreviation: Lancet
Journal ID: Lancet
NLM Journal ID: 2985213R

Lancet (London, England)

ISSN: 0140-6736
EISSN: 1474-547X

ARTICLE INFORMATION
==============================
PMCID: PMC8321427
PMID: 34332681
DOI: 10.1016/S0140-6736(21)01690-1
Article ID: S0140-6736(21)01690-1
Article version: 2
Subjects: Department of Error

Department of Error

Print publication date: 2021 Jul 31
Volume: 398
Issue: 10298
First page: 390
Last page: 390
Copyright: © 2021 The Author(s). Published by Elsevier Ltd.
Copyright year: 2021
Copyright holder: Elsevier Ltd
License: This is an open access article under the CC BY license (http://creativecommons.org/licenses/by/4.0/).
License URL: https://creativecommons.org/licenses/by/4.0/
Erratum for: Characterisation of in-hospital complications associated with COVID-19 using the ISARIC WHO Clinical Characterisation Protocol UK: a prospective, multicentre cohort study 398 10296 17 7 2021 223 237 Lancet (London, England) 10.1016/S0140-6736(21)00799-6 PMC8285118 34274064

==============================
Drake TM, Riad AM, Fairfield CJ, et al. Characterisation of in-hospital complications associated with COVID-19 using the ISARIC WHO Clinical Characterisation Protocol UK: a prospective, multicentre, cohort study. Lancet 2021; 398: 223–37—In the Summary of this Article, the percentage of gastrointestinal or liver complications reported should have been 10·8%. This correction has been made to the online version as of July 29, 2021.

