# Supplementary material for: Characterisation of in-hospital complications associated with COVID-19 using the ISARIC WHO Clinical Characterisation Protocol UK: a prospective, multicentre cohort study
Source: Lancet. 2021 Jul 17;398(10296):223–37. doi: 10.1016/S0140-6736(21)00799-6 (PMC8285118; doi:10.1016/S0140-6736(21)00799-6)

THE LANCET

Supplementary appendix

This appendix formed part of the original submission and has been peer reviewed. We post it as supplied by the authors.

Supplement to: Drake TM, Riad AM, Fairfield CJ, et al. Characterisation of in-hospital complications associated with COVID-19 using the ISARIC WHO Clinical Characterisation Protocol UK: a prospective, multicentre cohort study. *Lancet* 2021; **398:** 223–37.

Contents

Supplementary table 1 – Definition of in-hospital complications 1
Supplementary table 2 – Complication rates by SARS-CoV-2 RT-PCR positivity status 3
Supplementary table 3A - Specific complications stratified by age and sex in adults admitted to hospital with severe COVID-19. 4
Supplementary table 3B – Comparison between suspected and highly likely bacterial pneumonia by age and sex in adults admitted to hospital with severe COVID-19. 6
Supplementary table 3C- Comparison between suspected and highly likely bacterial pneumonia by number of existing comorbidities in adults admitted to hospital with severe COVID-19. 7
Supplementary table 4 - Organ specific complications stratified by age and presence of comorbidity in adults admitted to hospital with severe COVID-19 in adults admitted to hospital with severe COVID-19. 8
Supplementary table 5 - Specific complications stratified by age and presence of comorbidity in adults admitted to hospital with severe COVID-19. 9
Supplementary table 6 - Outcomes by organ specific complications in adults admitted to hospital with severe COVID-19 who survived. 12
Supplementary table 7 - Outcomes by organ specific complications in adults admitted to hospital with severe COVID-19 who died. 13
Supplementary table 8 - Effect of complications on survival (adjusted Cox proportional hazards models) for figure 2C..14
Supplementary table 8A - Any complication 14
Supplementary table 8B - Systemic complications 15
Supplementary table 8C - Renal complications/ acute kidney injury 16
Supplementary table 8D - Gastrointestinal and liver complications..17
Supplementary table 8E - Cardiovascular complications 18
Supplementary table 8F - Neurological complications 19
Supplementary table 8G - Respiratory complications 20
Supplementary table 9 - Effect of complications on odds of critical care admission (adjusted logistic regression models) for figure 2D 21
Supplementary table 9A - Any complication 21
Supplementary table 9B - Systemic complications 22
Supplementary table 9C - Renal complications/ acute kidney injury 23
Supplementary table 9D - Gastrointestinal and liver complications..24
Supplementary table 9E - Cardiovascular complications 25

Supplementary table 9F - Neurological complications 26
Supplementary table 9G - Respiratory complications 27
Supplementary table 10 - Effect of complications on odds of worse ability to self-care at discharge (adjusted logistic regression models) for figure 4C..28
Supplementary table 10A - Any complication..28
Supplementary table 10B - Systemic complications...29
Supplementary table 10C - Renal complications/ acute kidney injury 30
Supplementary table 10D - Gastrointestinal and liver complications....................................31
Supplementary table 10E - Cardiovascular complications 32
Supplementary table 10F - Neurological complications 33
Supplementary table 10G - Respiratory complications 34
Supplementary figure 1 – Heatmap of co-occurence of complications. Measured using the Jaccard similarity index, where 1 is perfect co-occurrence and 0 is no co-occurrence. 35
Supplementary figure 2 - Adjusted effect of age and sex on organ specific complications in adults with severe COVID-19 (also adjusted for centre as a random effect). 36
Supplementary figure 3 - Effect of age, sex and deprivation on specific complications in adults with COVID-19 (also adjusted for centre as a random effect). 37
Supplementary figure 3 (continued) - Effect of age, sex and deprivation on specific complications in adults with COVID-19 (also adjusted for centre as a random effect). 38
Supplementary figure 4 39
Supplementary figure 5 40
Supplementary figure 6 41
Supplementary figure 7 42
Supplementary figure 8 43
Supplementary figure 9 44
Supplementary figure 11 45
Supplementary figure 12 46
Supplementary figure 13 47

Supplementary tables

Supplementary table 1 – Definition of in-hospital complications

Organ specific complication	Complication within this group	Definition
Respiratory	Bacterial pneumonia (highly likely)	Clinically significant organism detected in sputum or deep respiratory culture
	Bacterial pneumonia (suspected)	Defined clinically in patient healthcare record
	Likely Acute Respiratory Distress Syndrome (ARDS)	Defined clinically in patient record or defined as one of the following combinations:<br>- receiving extracorporeal membrane oxygenation<br>- being nursed in a prone position and receiving invasive mechanical ventilation<br>- receiving mechanical ventilation and having a PF (*PaO2/FiO2*) ratio of 300 or less
	Empyema	Defined clinically in patient healthcare record
	Pneumothorax	Defined clinically in patient healthcare record
	Pleural effusion	Defined clinically in patient healthcare record
Neurological	Meningitis	Defined clinically in patient healthcare record
	Encephalitis	Defined clinically in patient healthcare record
	Seizure	Defined clinically in patient healthcare record
	Stroke	Defined clinically in patient healthcare record
Cardiovascular	Thromboembolism	Defined clinically in patient healthcare record
	Heart Failure	Defined clinically in patient healthcare record
	Myocarditis	Defined clinically in patient healthcare record
	Endocarditis	Defined clinically in patient healthcare record
	Arrhythmia	Defined clinically in patient healthcare record
	Cardiomyopathy	Defined clinically in patient healthcare record
	Myocardial Ischaemia	Defined clinically in patient healthcare record
	Cardiac Arrest	Defined clinically in patient healthcare record
Renal	Acute Kidney Injury	Creatinine rise which corresponded to Kidney Disease Improving Global Outcomes (KDIGO) stage I or above (creatinine rise ≥1.5x baseline value or by ≥26.5 µmol/L)<br><br>Urine output not included within this definition
Gastrointestinal	Acute Liver Injury	Any of the following:<br>- International normalised ratio (INR) rise of 2.5 times or greater than the lowest entered value<br>- INR of over 4.5 (in the absence of warfarin therapy)

		- Alanine aminotransferase (ALT) rise of greater than 10 times the lowest value<br>- ALT over 150<br>- Bilirubin rise of greater than 15<br>- Bilirubin greater than 55 (in the absence of any pre-existing liver disease)
	Pancreatitis	Defined clinically in patient healthcare record
	Gastrointestinal haemorrhage	Defined clinically in patient healthcare record
Systemic	Coagulopathy	Defined clinically in patient healthcare record
	Disseminated Intravascular Coagulation	Defined clinically in patient healthcare record
	Anaemia	Defined clinically in patient healthcare record
	Bloodstream Infection	Clinically significant sample in blood culture

Supplementary table 2 – Complication rates by SARS-CoV-2 RT-PCR positivity status

		Total	Any complication	Systemic	Renal	Gastrointestinal	Cardiovascular	Neurological	Respiratory
Total N (%)		73197	36367 (49.7)	11895 (16.3)	17752 (24.3)	7901 (10.8)	8973 (12.3)	3115 (4.3)	13486 (18.4)
PCR	Confirmed	62894 (85.9)	32044 (50.9)	10617 (16.9)	15829 (25.2)	6916 (11.0)	7935 (12.6)	2753 (4.4)	11849 (18.8)
	Not confirmed	10303 (14.1)	4323 (42.0)	1278 (12.4)	1923 (18.7)	985 (9.6)	1038 (10.1)	362 (3.5)	1637 (15.9)

Percentage values are row percentages, except for totals which represent column percentages.

Supplementary table 3A - Specific complications stratified by age and sex in adults admitted to hospital with severe COVID-19.

			Age (years)							
		Total	19-29	30-39	40-49	50-59	60-69	70-79	80-89	90+
Female	Total N (%)	31977	882 (2.8)	1359 (4.2)	1960 (6.1)	3488 (10.9)	4231 (13.2)	6685 (20.9)	9178 (28.7)	4194 (13.1)
	Cryptogenic organizing pneumonia	42 (0.1)	0 (0.0)	2 (0.1)	2 (0.1)	4 (0.1)	8 (0.2)	6 (0.1)	10 (0.1)	10 (0.2)
	Likely ARDS	3251 (10.2)	53 (6.0)	145 (10.7)	313 (16.0)	550 (15.8)	703 (16.6)	662 (9.9)	602 (6.6)	223 (5.3)
	Pneumothorax	227 (0.7)	6 (0.7)	11 (0.8)	25 (1.3)	39 (1.1)	57 (1.3)	52 (0.8)	28 (0.3)	9 (0.2)
	Pleural effusion	1940 (6.1)	16 (1.8)	47 (3.5)	84 (4.3)	143 (4.1)	269 (6.4)	421 (6.3)	641 (7.0)	319 (7.6)
	Highly likely Bacterial Pneumonia	99 (0.3)	2 (0.2)	4 (0.3)	14 (0.7)	25 (0.7)	21 (0.5)	21 (0.3)	10 (0.1)	2 (0.0)
	Meningitis / Encephalitis	61 (0.2)	2 (0.2)	4 (0.3)	7 (0.4)	7 (0.2)	19 (0.4)	8 (0.1)	11 (0.1)	3 (0.1)
	Seizure	331 (1.0)	6 (0.7)	17 (1.3)	22 (1.1)	46 (1.3)	46 (1.1)	79 (1.2)	87 (0.9)	28 (0.7)
	Stroke / Cerebrovascular accident	483 (1.5)	2 (0.2)	5 (0.4)	14 (0.7)	32 (0.9)	67 (1.6)	106 (1.6)	175 (1.9)	82 (2.0)
	Other neurological complication	537 (1.7)	7 (0.8)	8 (0.6)	22 (1.1)	42 (1.2)	69 (1.6)	105 (1.6)	201 (2.2)	83 (2.0)
	Congestive heart failure	1133 (3.5)	0 (0.0)	5 (0.4)	16 (0.8)	34 (1.0)	93 (2.2)	243 (3.6)	486 (5.3)	256 (6.1)
	Endocarditis / Myocarditis Pericarditis	83 (0.3)	1 (0.1)	5 (0.4)	6 (0.3)	11 (0.3)	19 (0.4)	23 (0.3)	14 (0.2)	4 (0.1)
	Myocarditis / Pericarditis	70 (0.2)	3 (0.3)	4 (0.3)	5 (0.3)	13 (0.4)	16 (0.4)	13 (0.2)	10 (0.1)	6 (0.1)
	Cardiomyopathy	79 (0.2)	0 (0.0)	1 (0.1)	6 (0.3)	10 (0.3)	8 (0.2)	16 (0.2)	29 (0.3)	9 (0.2)
	Cardiac arrhythmia	1907 (6.0)	15 (1.7)	30 (2.2)	87 (4.4)	140 (4.0)	226 (5.3)	429 (6.4)	680 (7.4)	300 (7.2)
	Cardiac ischemia	381 (1.2)	0 (0.0)	3 (0.2)	6 (0.3)	29 (0.8)	42 (1.0)	90 (1.3)	138 (1.5)	73 (1.7)
	Cardiac arrest	653 (2.0)	5 (0.6)	13 (1.0)	25 (1.3)	65 (1.9)	93 (2.2)	161 (2.4)	200 (2.2)	91 (2.2)
	Bloodstream infection	404 (1.3)	6 (0.7)	22 (1.6)	34 (1.7)	48 (1.4)	71 (1.7)	76 (1.1)	105 (1.1)	42 (1.0)
	Coagulation disorder / DIC	724 (2.3)	14 (1.6)	24 (1.8)	46 (2.3)	101 (2.9)	135 (3.2)	146 (2.2)	190 (2.1)	68 (1.6)
	Anaemia	4126 (12.9)	81 (9.2)	160 (11.8)	250 (12.8)	432 (12.4)	628 (14.8)	856 (12.8)	1188 (12.9)	531 (12.7)
	Rhabdomyolysis / Myositis	99 (0.3)	3 (0.3)	1 (0.1)	1 (0.1)	9 (0.3)	11 (0.3)	15 (0.2)	36 (0.4)	23 (0.5)
	Acute Kidney Injury	6612 (20.7)	51 (5.8)	124 (9.1)	251 (12.8)	642 (18.4)	933 (22.1)	1533 (22.9)	2137 (23.3)	941 (22.4)
	Gastrointestinal haemorrhage	325 (1.0)	5 (0.6)	8 (0.6)	8 (0.4)	25 (0.7)	48 (1.1)	57 (0.9)	120 (1.3)	54 (1.3)
	Pancreatitis	108 (0.3)	5 (0.6)	9 (0.7)	9 (0.5)	17 (0.5)	24 (0.6)	16 (0.2)	22 (0.2)	6 (0.1)
	Deep Vein Thrombosis	167 (0.5)	2 (0.2)	10 (0.7)	9 (0.5)	31 (0.9)	33 (0.8)	30 (0.4)	41 (0.4)	11 (0.3)
	Pulmonary Embolism	382 (1.2)	3 (0.3)	15 (1.1)	27 (1.4)	65 (1.9)	83 (2.0)	78 (1.2)	90 (1.0)	21 (0.5)
	Liver Injury	2366 (7.4)	48 (5.4)	113 (8.3)	192 (9.8)	409 (11.7)	430 (10.2)	475 (7.1)	500 (5.4)	199 (4.7)
Male	Total N (%)	41025	612 (1.5)	1385 (3.4)	3021 (7.4)	5591 (13.6)	6882 (16.8)	9841 (24.0)	10662 (26.0)	3031 (7.4)
	Cryptogenic organizing pneumonia	50 (0.1)	0 (0.0)	2 (0.1)	4 (0.1)	9 (0.2)	12 (0.2)	10 (0.1)	12 (0.1)	1 (0.0)
	Likely ARDS	6417 (15.6)	62 (10.1)	247 (17.8)	697 (23.1)	1376 (24.6)	1540 (22.4)	1411 (14.3)	889 (8.3)	195 (6.4)
	Pneumothorax	466 (1.1)	6 (1.0)	20 (1.4)	47 (1.6)	113 (2.0)	121 (1.8)	87 (0.9)	55 (0.5)	17 (0.6)
	Pleural effusion	2583 (6.3)	23 (3.8)	53 (3.8)	145 (4.8)	322 (5.8)	452 (6.6)	631 (6.4)	723 (6.8)	234 (7.7)
	Highly likely Bacterial Pneumonia	260 (0.6)	1 (0.2)	10 (0.7)	38 (1.3)	78 (1.4)	60 (0.9)	54 (0.5)	17 (0.2)	2 (0.1)
	Meningitis / Encephalitis	95 (0.2)	5 (0.8)	5 (0.4)	14 (0.5)	20 (0.4)	23 (0.3)	12 (0.1)	16 (0.2)	0 (0.0)
	Seizure	497 (1.2)	13 (2.1)	33 (2.4)	45 (1.5)	89 (1.6)	87 (1.3)	105 (1.1)	105 (1.0)	20 (0.7)
	Stroke / Cerebrovascular accident	729 (1.8)	1 (0.2)	12 (0.9)	36 (1.2)	75 (1.3)	147 (2.1)	205 (2.1)	209 (2.0)	44 (1.5)
	Other neurological complication	696 (1.7)	7 (1.1)	18 (1.3)	31 (1.0)	90 (1.6)	98 (1.4)	175 (1.8)	211 (2.0)	66 (2.2)

	Total	Age (years)							
		19-29	30-39	40-49	50-59	60-69	70-79	80-89	90+
Congestive heart failure	1387 (3.4)	0 (0.0)	11 (0.8)	27 (0.9)	84 (1.5)	184 (2.7)	363 (3.7)	539 (5.1)	179 (5.9)
Endocarditis / Myocarditis Pericarditis	181 (0.4)	1 (0.2)	12 (0.9)	19 (0.6)	29 (0.5)	44 (0.6)	39 (0.4)	36 (0.3)	1 (0.0)
Myocarditis / Pericarditis	121 (0.3)	1 (0.2)	3 (0.2)	20 (0.7)	19 (0.3)	22 (0.3)	28 (0.3)	22 (0.2)	6 (0.2)
Cardiomyopathy	94 (0.2)	0 (0.0)	3 (0.2)	9 (0.3)	13 (0.2)	20 (0.3)	24 (0.2)	21 (0.2)	4 (0.1)
Cardiac arrhythmia	2933 (7.1)	20 (3.3)	48 (3.5)	143 (4.7)	367 (6.6)	597 (8.7)	790 (8.0)	759 (7.1)	209 (6.9)
Cardiac ischemia	615 (1.5)	0 (0.0)	11 (0.8)	19 (0.6)	56 (1.0)	106 (1.5)	175 (1.8)	186 (1.7)	62 (2.0)
Cardiac arrest	1249 (3.0)	4 (0.7)	19 (1.4)	68 (2.3)	175 (3.1)	238 (3.5)	327 (3.3)	315 (3.0)	103 (3.4)
Bloodstream infection	710 (1.7)	9 (1.5)	29 (2.1)	65 (2.2)	99 (1.8)	142 (2.1)	154 (1.6)	166 (1.6)	46 (1.5)
Coagulation disorder / DIC	1343 (3.3)	13 (2.1)	56 (4.0)	131 (4.3)	258 (4.6)	272 (4.0)	292 (3.0)	253 (2.4)	68 (2.2)
Anaemia	5762 (14.0)	39 (6.4)	143 (10.3)	327 (10.8)	791 (14.1)	1043 (15.2)	1432 (14.6)	1534 (14.4)	453 (14.9)
Rhabdomyolysis / Myositis	165 (0.4)	5 (0.8)	6 (0.4)	12 (0.4)	24 (0.4)	18 (0.3)	42 (0.4)	41 (0.4)	17 (0.6)
Acute Kidney Injury	11097 (27.0)	75 (12.3)	228 (16.5)	620 (20.5)	1434 (25.6)	2116 (30.7)	2775 (28.2)	3007 (28.2)	842 (27.8)
Gastrointestinal haemorrhage	529 (1.3)	5 (0.8)	9 (0.6)	32 (1.1)	65 (1.2)	91 (1.3)	120 (1.2)	159 (1.5)	48 (1.6)
Pancreatitis	139 (0.3)	6 (1.0)	18 (1.3)	18 (0.6)	36 (0.6)	21 (0.3)	23 (0.2)	14 (0.1)	3 (0.1)
Deep Vein Thrombosis	261 (0.6)	1 (0.2)	7 (0.5)	25 (0.8)	59 (1.1)	55 (0.8)	57 (0.6)	44 (0.4)	13 (0.4)
Pulmonary Embolism	622 (1.5)	11 (1.8)	14 (1.0)	78 (2.6)	139 (2.5)	136 (2.0)	135 (1.4)	96 (0.9)	13 (0.4)
Liver Injury	4732 (11.5)	80 (13.1)	223 (16.1)	515 (17.0)	988 (17.7)	1027 (14.9)	998 (10.1)	714 (6.7)	187 (6.2)

Percentage values are row percentages, except for totals which represent column percentages. ARDS – Acute Respiratory Distress Syndrome, DIC – Disseminated Intravascular Coagulation.

Supplementary table 3B – Comparison between suspected and highly likely bacterial pneumonia by age and sex in adults admitted to hospital with severe COVID-19.

			Age group (years)							
		Total N	19-29	30-39	40-49	50-59	60-69	70-79	80-89	90+
Female	Total N (%)	31977	882 (2.8)	1359 (4.2)	1960 (6.1)	3488 (10.9)	4231 (13.2)	6685 (20.9)	9178 (28.7)	4194 (13.1)
	Suspected Bacterial Pneumonia	3533 (100)	43 (1.2)	101 (2.9)	188 (5.3)	403 (11.4)	539 (15.3)	771 (21.8)	1028 (29.1)	460 (13.0)
	Highly likely Bacterial Pneumonia	99 (100)	2 (2.0)	4 (4.0)	14 (14.1)	25 (25.3)	21 (21.2)	21 (21.2)	10 (10.1)	2 (2.0)
Male	Total N (%)	41025	612 (1.5)	1385 (3.4)	3021 (7.4)	5591 (13.6)	6882 (16.8)	9841 (24.0)	10662 (26.0)	3031 (7.4)
	Suspected Bacterial Pneumonia	5321 (100)	52 (1.0)	148 (2.8)	407 (7.6)	765 (14.4)	927 (17.4)	1281 (24.1)	1353 (25.4)	388 (7.3)
	Highly likely Bacterial Pneumonia	260 (100)	1 (0.4)	10 (3.8)	38 (14.6)	78 (30.0)	60 (23.1)	54 (20.8)	17 (6.5)	2 (0.8)

Percentage values are row percentages

Supplementary table 3C- Comparison between suspected and highly likely bacterial pneumonia by number of existing comorbidities in adults admitted to hospital with severe COVID-19.

Number of existing comorbidities on admission		Total N	Age group (years)							
			19-29	30-39	40-49	50-59	60-69	70-79	80-89	90+
0	Total N (%)	13908	862 (6.2)	1430 (10.3)	2140 (15.4)	2907 (20.9)	2341 (16.8)	1925 (13.8)	1700 (12.2)	603 (4.3)
	Suspected Bacterial Pneumonia	1478	44 (3.0)	112 (7.6)	239 (16.2)	356 (24.1)	312 (21.1)	211 (14.3)	154 (10.4)	50 (3.4)
	Highly likely Bacterial Pneumonia	111	1 (0.9)	6 (5.4)	23 (20.7)	39 (35.1)	21 (18.9)	19 (17.1)	2 (1.8)	0 (0.0)
1	Total N (%)	17635	434 (2.5)	780 (4.4)	1550 (8.8)	2850 (16.2)	3031 (17.2)	3786 (21.5)	3768 (21.4)	1436 (8.1)
	Suspected Bacterial Pneumonia	2105	33 (1.6)	76 (3.6)	199 (9.5)	369 (17.5)	390 (18.5)	458 (21.8)	425 (20.2)	155 (7.4)
	Highly likely Bacterial Pneumonia	107	1 (0.9)	5 (4.7)	14 (13.1)	40 (37.4)	23 (21.5)	19 (17.8)	4 (3.7)	1 (0.9)
2+	Total N (%)	41654	204 (0.5)	543 (1.3)	1306 (3.1)	3344 (8.0)	5767 (13.8)	10852 (26.1)	14432 (34.6)	5206 (12.5)
	Suspected Bacterial Pneumonia	5288	18 (0.3)	62 (1.2)	160 (3.0)	445 (8.4)	766 (14.5)	1389 (26.3)	1805 (34.1)	643 (12.2)
	Highly likely Bacterial Pneumonia	141	1 (0.7)	3 (2.1)	15 (10.6)	24 (17.0)	37 (26.2)	37 (26.2)	21 (14.9)	3 (2.1)

Percentage values are row percentages

Supplementary table 4 - Organ specific complications stratified by age and presence of comorbidity in adults admitted to hospital with severe COVID-19 in adults admitted to hospital with severe COVID-19.

Number of existing comorbidities on admission		Total	Age group (years)							
			19-29	30-39	40-49	50-59	60-69	70-79	80-89	90+
0	Total N (%)	13908	862 (6.2)	1430 (10.3)	2140 (15.4)	2907 (20.9)	2341 (16.8)	1925 (13.8)	1700 (12.2)	603 (4.3)
	Systemic	1614 (11.6)	61 (7.1)	153 (10.7)	245 (11.4)	368 (12.7)	334 (14.3)	233 (12.1)	159 (9.4)	61 (10.1)
	Renal	2170 (15.6)	45 (5.2)	122 (8.5)	273 (12.8)	497 (17.1)	497 (21.2)	365 (19.0)	281 (16.5)	90 (14.9)
	Gastrointestinal	1657 (11.9)	64 (7.4)	162 (11.3)	299 (14.0)	459 (15.8)	320 (13.7)	210 (10.9)	111 (6.5)	32 (5.3)
	Cardiovascular	1046 (7.5)	21 (2.4)	46 (3.2)	134 (6.3)	212 (7.3)	219 (9.4)	199 (10.3)	162 (9.5)	53 (8.8)
	Neurological	409 (2.9)	12 (1.4)	35 (2.4)	52 (2.4)	75 (2.6)	78 (3.3)	65 (3.4)	79 (4.6)	13 (2.2)
	Respiratory	2562 (18.4)	61 (7.1)	185 (12.9)	462 (21.6)	676 (23.3)	596 (25.5)	372 (19.3)	164 (9.6)	46 (7.6)
	Any complication	5328 (38.3)	184 (21.3)	427 (29.9)	816 (38.1)	1184 (40.7)	1059 (45.2)	811 (42.1)	650 (38.2)	197 (32.7)
1	Total N (%)	17635	434 (2.5)	780 (4.4)	1550 (8.8)	2850 (16.2)	3031 (17.2)	3786 (21.5)	3768 (21.4)	1436 (8.1)
	Systemic	2495 (14.1)	49 (11.3)	113 (14.5)	218 (14.1)	439 (15.4)	516 (17.0)	508 (13.4)	468 (12.4)	184 (12.8)
	Renal	3747 (21.2)	42 (9.7)	105 (13.5)	261 (16.8)	603 (21.2)	796 (26.3)	867 (22.9)	795 (21.1)	278 (19.4)
	Gastrointestinal	2021 (11.5)	50 (11.5)	112 (14.4)	235 (15.2)	450 (15.8)	454 (15.0)	380 (10.0)	244 (6.5)	96 (6.7)
	Cardiovascular	1801 (10.2)	14 (3.2)	52 (6.7)	103 (6.6)	255 (8.9)	353 (11.6)	427 (11.3)	417 (11.1)	180 (12.5)
	Neurological	693 (3.9)	17 (3.9)	27 (3.5)	52 (3.4)	105 (3.7)	141 (4.7)	145 (3.8)	155 (4.1)	51 (3.6)
	Respiratory	3416 (19.4)	55 (12.7)	155 (19.9)	331 (21.4)	716 (25.1)	797 (26.3)	707 (18.7)	488 (13.0)	167 (11.6)
	Any complication	8198 (46.5)	142 (32.7)	316 (40.5)	653 (42.1)	1334 (46.8)	1555 (51.3)	1814 (47.9)	1730 (45.9)	654 (45.5)
2+	Total N (%)	41654	204 (0.5)	543 (1.3)	1306 (3.1)	3344 (8.0)	5767 (13.8)	10852 (26.1)	14432 (34.6)	5206 (12.5)
	Systemic	7786 (18.7)	37 (18.1)	110 (20.3)	268 (20.5)	697 (20.8)	1158 (20.1)	1986 (18.3)	2614 (18.1)	916 (17.6)
	Renal	11835 (28.4)	39 (19.1)	126 (23.2)	340 (26.0)	978 (29.2)	1762 (30.6)	3086 (28.4)	4085 (28.3)	1419 (27.3)
	Gastrointestinal	4223 (10.1)	25 (12.3)	91 (16.8)	206 (15.8)	559 (16.7)	804 (13.9)	1054 (9.7)	1123 (7.8)	361 (6.9)
	Cardiovascular	6126 (14.7)	12 (5.9)	36 (6.6)	133 (10.2)	380 (11.4)	817 (14.2)	1594 (14.7)	2309 (16.0)	845 (16.2)
	Neurological	2013 (4.8)	9 (4.4)	29 (5.3)	58 (4.4)	172 (5.1)	281 (4.9)	515 (4.7)	707 (4.9)	242 (4.6)
	Respiratory	7508 (18.0)	29 (14.2)	117 (21.5)	376 (28.8)	871 (26.0)	1374 (23.8)	1899 (17.5)	2109 (14.6)	733 (14.1)
	Any complication	22841 (54.8)	85 (41.7)	272 (50.1)	701 (53.7)	1900 (56.8)	3340 (57.9)	5924 (54.6)	7827 (54.2)	2792 (53.6)

Percentage values are row percentages, except for totals which represent column percentages

Supplementary table 5 - Specific complications stratified by age and presence of comorbidity in adults admitted to hospital with severe COVID-19.

			Age group (years)							
Number of existing comorbidities on admission		Total	19-29	30-39	40-49	50-59	60-69	70-79	80-89	90+
0	Total N (%)	13908	862 (6.2)	1430 (10.3)	2140 (15.4)	2907 (20.9)	2341 (16.8)	1925 (13.8)	1700 (12.2)	603 (4.3)
	Empyema	12 (0.1)	0 (0.0)	1 (0.1)	2 (0.1)	2 (0.1)	4 (0.2)	2 (0.1)	1 (0.1)	0 (0.0)
	Likely ARDS	2167 (15.6)	44 (5.1)	156 (10.9)	405 (18.9)	593 (20.4)	525 (22.4)	310 (16.1)	113 (6.6)	21 (3.5)
	Pneumothorax	173 (1.2)	9 (1.0)	14 (1.0)	30 (1.4)	51 (1.8)	44 (1.9)	21 (1.1)	2 (0.1)	2 (0.3)
	Pleural effusion	566 (4.1)	21 (2.4)	41 (2.9)	89 (4.2)	122 (4.2)	120 (5.1)	93 (4.8)	56 (3.3)	24 (4.0)
	Highly likely Bacterial Pneumonia	111 (0.8)	1 (0.1)	6 (0.4)	23 (1.1)	39 (1.3)	21 (0.9)	19 (1.0)	2 (0.1)	0 (0.0)
	Meningitis / Encephalitis	41 (0.3)	5 (0.6)	6 (0.4)	6 (0.3)	6 (0.2)	9 (0.4)	4 (0.2)	4 (0.2)	1 (0.2)
	Seizure	106 (0.8)	6 (0.7)	18 (1.3)	18 (0.8)	24 (0.8)	14 (0.6)	12 (0.6)	13 (0.8)	1 (0.2)
	Stroke / Cerebrovascular accident	189 (1.4)	0 (0.0)	7 (0.5)	18 (0.8)	28 (1.0)	42 (1.8)	43 (2.2)	45 (2.6)	6 (1.0)
	Other neurological complication	117 (0.8)	2 (0.2)	7 (0.5)	17 (0.8)	25 (0.9)	26 (1.1)	13 (0.7)	21 (1.2)	6 (1.0)
	Congestive heart failure	111 (0.8)	0 (0.0)	5 (0.3)	7 (0.3)	16 (0.6)	16 (0.7)	23 (1.2)	27 (1.6)	17 (2.8)
	Endocarditis / Myocarditis Pericarditis	52 (0.4)	0 (0.0)	7 (0.5)	10 (0.5)	8 (0.3)	17 (0.7)	6 (0.3)	3 (0.2)	1 (0.2)
	Myocarditis / Pericarditis	41 (0.3)	2 (0.2)	3 (0.2)	11 (0.5)	13 (0.4)	7 (0.3)	2 (0.1)	3 (0.2)	0 (0.0)
	Cardiomyopathy	16 (0.1)	0 (0.0)	2 (0.1)	3 (0.1)	3 (0.1)	5 (0.2)	1 (0.1)	2 (0.1)	0 (0.0)
	Cardiac arrhythmia	672 (4.8)	15 (1.7)	28 (2.0)	90 (4.2)	138 (4.7)	157 (6.7)	130 (6.8)	86 (5.1)	28 (4.6)
	Cardiac ischaemia	84 (0.6)	0 (0.0)	8 (0.6)	7 (0.3)	13 (0.4)	14 (0.6)	16 (0.8)	21 (1.2)	5 (0.8)
	Cardiac arrest	269 (1.9)	7 (0.8)	6 (0.4)	35 (1.6)	54 (1.9)	50 (2.1)	63 (3.3)	44 (2.6)	10 (1.7)
	Bloodstream Infection	193 (1.4)	3 (0.3)	20 (1.4)	36 (1.7)	38 (1.3)	43 (1.8)	26 (1.4)	20 (1.2)	7 (1.2)
	Coagulation disorder / DIC	383 (2.8)	10 (1.2)	40 (2.8)	71 (3.3)	92 (3.2)	79 (3.4)	55 (2.9)	27 (1.6)	9 (1.5)
	Anaemia	1253 (9.0)	53 (6.1)	114 (8.0)	180 (8.4)	288 (9.9)	262 (11.2)	191 (9.9)	116 (6.8)	49 (8.1)
	Rhabdomyolysis / Myositis	45 (0.3)	2 (0.2)	3 (0.2)	8 (0.4)	9 (0.3)	4 (0.2)	8 (0.4)	6 (0.4)	5 (0.8)
	Acute Kidney Injury	2170 (15.6)	45 (5.2)	122 (8.5)	273 (12.8)	497 (17.1)	497 (21.2)	365 (19.0)	281 (16.5)	90 (14.9)
	Gastrointestinal haemorrhage	87 (0.6)	6 (0.7)	5 (0.3)	7 (0.3)	21 (0.7)	21 (0.9)	8 (0.4)	14 (0.8)	5 (0.8)
	Pancreatitis	43 (0.3)	5 (0.6)	10 (0.7)	4 (0.2)	11 (0.4)	5 (0.2)	5 (0.3)	1 (0.1)	2 (0.3)
	Deep Vein Thrombosis	99 (0.7)	2 (0.2)	8 (0.6)	15 (0.7)	31 (1.1)	25 (1.1)	10 (0.5)	6 (0.4)	2 (0.3)
	Pulmonary Embolism	257 (1.8)	5 (0.6)	19 (1.3)	48 (2.2)	78 (2.7)	54 (2.3)	32 (1.7)	19 (1.1)	2 (0.3)
	Liver Injury	1566 (11.3)	58 (6.7)	150 (10.5)	293 (13.7)	440 (15.1)	302 (12.9)	201 (10.4)	97 (5.7)	25 (4.1)
1	Total N (%)	17635	434 (2.5)	780 (4.4)	1550 (8.8)	2850 (16.2)	3031 (17.2)	3786 (21.5)	3768 (21.4)	1436 (8.1)
	Empyema	23 (0.1)	0 (0.0)	3 (0.4)	2 (0.1)	3 (0.1)	6 (0.2)	3 (0.1)	3 (0.1)	3 (0.2)
	Likely ARDS	2653 (15.0)	49 (11.3)	132 (16.9)	293 (18.9)	627 (22.0)	677 (22.3)	526 (13.9)	275 (7.3)	74 (5.2)

Number of existing comorbidities on admission		Total	Age group (years) 19-29	30-39	40-49	50-59	60-69	70-79	80-89	90+
	Pneumothorax	208 (1.2)	2 (0.5)	8 (1.0)	22 (1.4)	55 (1.9)	59 (1.9)	41 (1.1)	16 (0.4)	5 (0.3)
	Pleural effusion	959 (5.4)	10 (2.3)	34 (4.4)	56 (3.6)	129 (4.5)	187 (6.2)	217 (5.7)	226 (6.0)	100 (7.0)
	Highly likely Bacterial Pneumonia	107 (0.6)	1 (0.2)	5 (0.6)	14 (0.9)	40 (1.4)	23 (0.8)	19 (0.5)	4 (0.1)	1 (0.1)
	Meningitis / Encephalitis	33 (0.2)	1 (0.2)	3 (0.4)	3 (0.2)	6 (0.2)	10 (0.3)	7 (0.2)	3 (0.1)	0 (0.0)
	Seizure	178 (1.0)	7 (1.6)	16 (2.1)	26 (1.7)	34 (1.2)	36 (1.2)	20 (0.5)	29 (0.8)	10 (0.7)
	Stroke / Cerebrovascular accident	293 (1.7)	2 (0.5)	3 (0.4)	16 (1.0)	33 (1.2)	55 (1.8)	81 (2.1)	76 (2.0)	27 (1.9)
	Other neurological complication	262 (1.5)	9 (2.1)	11 (1.4)	17 (1.1)	44 (1.5)	51 (1.7)	53 (1.4)	61 (1.6)	16 (1.1)
	Congestive heart failure	316 (1.8)	0 (0.0)	5 (0.6)	9 (0.6)	24 (0.8)	47 (1.6)	78 (2.1)	90 (2.4)	63 (4.4)
	Endocarditis / Myocarditis Pericarditis	70 (0.4)	0 (0.0)	8 (1.0)	6 (0.4)	14 (0.5)	19 (0.6)	13 (0.3)	8 (0.2)	2 (0.1)
	Myocarditis / Pericarditis	53 (0.3)	1 (0.2)	2 (0.3)	8 (0.5)	8 (0.3)	12 (0.4)	13 (0.3)	7 (0.2)	2 (0.1)
	Cardiomyopathy	34 (0.2)	0 (0.0)	1 (0.1)	5 (0.3)	8 (0.3)	5 (0.2)	8 (0.2)	6 (0.2)	1 (0.1)
	Cardiac arrhythmia	1082 (6.1)	12 (2.8)	28 (3.6)	66 (4.3)	159 (5.6)	229 (7.6)	268 (7.1)	231 (6.1)	89 (6.2)
	Cardiac ischaemia	176 (1.0)	0 (0.0)	5 (0.6)	7 (0.5)	23 (0.8)	37 (1.2)	45 (1.2)	43 (1.1)	16 (1.1)
	Cardiac arrest	461 (2.6)	1 (0.2)	16 (2.1)	27 (1.7)	87 (3.1)	96 (3.2)	98 (2.6)	95 (2.5)	41 (2.9)
	Bloodstream Infection	296 (1.7)	9 (2.1)	19 (2.4)	34 (2.2)	58 (2.0)	61 (2.0)	53 (1.4)	48 (1.3)	14 (1.0)
	Coagulation disorder / DIC	489 (2.8)	12 (2.8)	22 (2.8)	51 (3.3)	106 (3.7)	126 (4.2)	96 (2.5)	56 (1.5)	20 (1.4)
	Anaemia	2012 (11.4)	34 (7.8)	92 (11.8)	173 (11.2)	351 (12.3)	419 (13.8)	407 (10.8)	380 (10.1)	156 (10.9)
	Rhabdomyolysis / Myositis	54 (0.3)	4 (0.9)	1 (0.1)	4 (0.3)	9 (0.3)	7 (0.2)	14 (0.4)	13 (0.3)	2 (0.1)
	Acute Kidney Injury	3747 (21.2)	42 (9.7)	105 (13.5)	261 (16.8)	603 (21.2)	796 (26.3)	867 (22.9)	795 (21.1)	278 (19.4)
	Gastrointestinal haemorrhage	187 (1.1)	2 (0.5)	4 (0.5)	21 (1.4)	29 (1.0)	38 (1.3)	29 (0.8)	41 (1.1)	23 (1.6)
	Pancreatitis	67 (0.4)	3 (0.7)	7 (0.9)	10 (0.6)	17 (0.6)	14 (0.5)	8 (0.2)	7 (0.2)	1 (0.1)
	Deep Vein Thrombosis	102 (0.6)	0 (0.0)	2 (0.3)	14 (0.9)	19 (0.7)	26 (0.9)	26 (0.7)	13 (0.3)	2 (0.1)
	Pulmonary Embolism	261 (1.5)	6 (1.4)	6 (0.8)	31 (2.0)	50 (1.8)	64 (2.1)	51 (1.3)	47 (1.2)	6 (0.4)
	Liver Injury	1859 (10.5)	48 (11.1)	105 (13.5)	220 (14.2)	430 (15.1)	425 (14.0)	351 (9.3)	205 (5.4)	75 (5.2)
2+	Total N (%)	41654	204 (0.5)	543 (1.3)	1306 (3.1)	3344 (8.0)	5767 (13.8)	10852 (26.1)	14432 (34.6)	5206 (12.5)
	Empyema	57 (0.1)	0 (0.0)	0 (0.0)	2 (0.2)	8 (0.2)	10 (0.2)	11 (0.1)	18 (0.1)	8 (0.2)
	Likely ARDS	4871 (11.7)	23 (11.3)	106 (19.5)	314 (24.0)	709 (21.2)	1043 (18.1)	1243 (11.5)	1108 (7.7)	325 (6.2)
	Pneumothorax	313 (0.8)	1 (0.5)	9 (1.7)	21 (1.6)	46 (1.4)	75 (1.3)	77 (0.7)	65 (0.5)	19 (0.4)
	Pleural effusion	3006 (7.2)	8 (3.9)	25 (4.6)	85 (6.5)	214 (6.4)	416 (7.2)	743 (6.8)	1085 (7.5)	430 (8.3)
	Highly likely Bacterial Pneumonia	141 (0.3)	1 (0.5)	3 (0.6)	15 (1.1)	24 (0.7)	37 (0.6)	37 (0.3)	21 (0.1)	3 (0.1)
	Meningitis / Encephalitis	82 (0.2)	1 (0.5)	0 (0.0)	12 (0.9)	15 (0.4)	23 (0.4)	9 (0.1)	20 (0.1)	2 (0.0)
	Seizure	545 (1.3)	6 (2.9)	16 (2.9)	24 (1.8)	77 (2.3)	83 (1.4)	152 (1.4)	150 (1.0)	37 (0.7)

Number of existing comorbidities on admission	Total	Age group (years)							
		19-29	30-39	40-49	50-59	60-69	70-79	80-89	90+
Stroke / Cerebrovascular accident	731 (1.8)	1 (0.5)	7 (1.3)	16 (1.2)	46 (1.4)	117 (2.0)	187 (1.7)	264 (1.8)	93 (1.8)
Other neurological complication	856 (2.1)	4 (2.0)	8 (1.5)	19 (1.5)	63 (1.9)	90 (1.6)	214 (2.0)	331 (2.3)	127 (2.4)
Congestive heart failure	2101 (5.0)	0 (0.0)	6 (1.1)	27 (2.1)	78 (2.3)	214 (3.7)	506 (4.7)	914 (6.3)	356 (6.8)
Endocarditis / Myocarditis Pericarditis	142 (0.3)	2 (1.0)	2 (0.4)	9 (0.7)	18 (0.5)	27 (0.5)	43 (0.4)	39 (0.3)	2 (0.0)
Myocarditis / Pericarditis	97 (0.2)	1 (0.5)	2 (0.4)	6 (0.5)	11 (0.3)	19 (0.3)	26 (0.2)	22 (0.2)	10 (0.2)
Cardiomyopathy	124 (0.3)	0 (0.0)	2 (0.4)	7 (0.5)	12 (0.4)	18 (0.3)	31 (0.3)	42 (0.3)	12 (0.2)
Cardiac arrhythmia	3098 (7.4)	8 (3.9)	22 (4.1)	74 (5.7)	210 (6.3)	438 (7.6)	824 (7.6)	1129 (7.8)	393 (7.5)
Cardiac ischaemia	738 (1.8)	0 (0.0)	1 (0.2)	11 (0.8)	49 (1.5)	97 (1.7)	204 (1.9)	261 (1.8)	115 (2.2)
Cardiac arrest	1173 (2.8)	1 (0.5)	10 (1.8)	31 (2.4)	99 (3.0)	185 (3.2)	327 (3.0)	377 (2.6)	143 (2.7)
Bloodstream Infection	626 (1.5)	3 (1.5)	12 (2.2)	30 (2.3)	51 (1.5)	109 (1.9)	151 (1.4)	203 (1.4)	67 (1.3)
Coagulation disorder / DIC	1199 (2.9)	5 (2.5)	19 (3.5)	55 (4.2)	161 (4.8)	202 (3.5)	288 (2.7)	362 (2.5)	107 (2.1)
Anaemia	6641 (15.9)	33 (16.2)	97 (17.9)	226 (17.3)	586 (17.5)	991 (17.2)	1693 (15.6)	2232 (15.5)	783 (15.0)
Rhabdomyolysis / Myositis	167 (0.4)	2 (1.0)	3 (0.6)	2 (0.2)	15 (0.4)	18 (0.3)	35 (0.3)	59 (0.4)	33 (0.6)
Acute Kidney Injury	11835 (28.4)	39 (19.1)	126 (23.2)	340 (26.0)	978 (29.2)	1762 (30.6)	3086 (28.4)	4085 (28.3)	1419 (27.3)
Gastrointestinal haemorrhage	581 (1.4)	2 (1.0)	8 (1.5)	12 (0.9)	40 (1.2)	80 (1.4)	141 (1.3)	224 (1.6)	74 (1.4)
Pancreatitis	138 (0.3)	3 (1.5)	10 (1.8)	13 (1.0)	25 (0.7)	26 (0.5)	26 (0.2)	29 (0.2)	6 (0.1)
Deep Vein Thrombosis	228 (0.5)	1 (0.5)	7 (1.3)	5 (0.4)	40 (1.2)	37 (0.6)	52 (0.5)	66 (0.5)	20 (0.4)
Pulmonary Embolism	486 (1.2)	3 (1.5)	4 (0.7)	26 (2.0)	76 (2.3)	101 (1.8)	130 (1.2)	120 (0.8)	26 (0.5)
Liver Injury	3683 (8.8)	22 (10.8)	81 (14.9)	194 (14.9)	530 (15.8)	733 (12.7)	922 (8.5)	915 (6.3)	286 (5.5)

Percentage values are row percentages, except for totals which represent column percentages. ARDS – Acute Respiratory Distress Syndrome, DIC – Disseminated Intravascular Coagulation.

Supplementary table 6 - Outcomes by organ specific complications in adults admitted to hospital with severe COVID-19 who survived.

		Total number of patients experiencing complications		Organ-specific level complications§					
		Total patients	Any complication	Systemic	Renal	Gastrointestinal	Cardiovascular	Neurological	Respiratory
Total N (%)		50105	21784 (43.5)	7423 (14.8)	10059 (20.1)	4837 (9.7)	4035 (8.1)	1880 (3.8)	7028 (14.0)
Critical Care Admission	No	43153 (86.1)	16853 (77.4)	5557 (74.9)	7467 (74.2)	3233 (66.8)	3020 (74.8)	1461 (77.7)	3616 (51.5)
	Yes	6242 (12.5)	4926 (22.6)	1866 (25.1)	2589 (25.7)	1602 (33.1)	1015 (25.2)	419 (22.3)	3410 (48.5)
	(Missing)	710 (1.4)	5 (0.0)	0 (0.0)	3 (0.0)	2 (0.0)	0 (0.0)	0 (0.0)	2 (0.0)
Any invasive ventilation	No	45825 (91.5)	18630 (85.5)	6067 (81.7)	8271 (82.2)	3728 (77.1)	3275 (81.2)	1533 (81.5)	4498 (64.0)
	Yes	3472 (6.9)	3130 (14.4)	1352 (18.2)	1773 (17.6)	1104 (22.8)	758 (18.8)	347 (18.5)	2529 (36.0)
	(Missing)	808 (1.6)	24 (0.1)	4 (0.1)	15 (0.1)	5 (0.1)	2 (0.0)	0 (0.0)	1 (0.0)
Any non-invasive ventilation	No	42350 (87.2)	17255 (80.1)	5917 (80.3)	7738 (77.8)	3568 (74.6)	3184 (79.6)	1566 (84.3)	4232 (60.6)
	Yes	6206 (12.8)	4296 (19.9)	1454 (19.7)	2207 (22.2)	1218 (25.4)	817 (20.4)	291 (15.7)	2748 (39.4)
Any oxygen	No	15507 (31.8)	4892 (22.6)	1748 (23.7)	1942 (19.4)	940 (19.5)	888 (22.2)	580 (31.1)	627 (8.9)
	Yes	33310 (68.2)	16760 (77.4)	5642 (76.3)	8061 (80.6)	3873 (80.5)	3120 (77.8)	1285 (68.9)	6379 (91.1)

Percentage values are row percentages, except for totals which represent column percentages. § = Denominator is number of non-missing observations for each organ system.

Supplementary table 7 - Outcomes by organ specific complications in adults admitted to hospital with severe COVID-19 who died.

		Total number of patients experiencing complications		Organ-specific level complications§					
		Total	Any complication	Systemic	Renal	Gastrointestinal	Cardiovascular	Neurological	Respiratory
Total N (%)		23092	14583 (63.2)	4472 (19.4)	7693 (33.3)	3064 (13.3)	4938 (21.4)	1235 (5.3)	6458 (28.0)
Critical Care Admission	No	18972 (82.2)	11239 (77.1)	3247 (72.6)	5525 (71.8)	1906 (62.2)	3620 (73.3)	985 (79.8)	3856 (59.7)
	Yes	3792 (16.4)	3341 (22.9)	1224 (27.4)	2166 (28.2)	1158 (37.8)	1318 (26.7)	249 (20.2)	2602 (40.3)
	(Missing)	328 (1.4)	3 (0.0)	1 (0.0)	2 (0.0)	0 (0.0)	0 (0.0)	1 (0.1)	0 (0.0)
Any invasive ventilation	No	20063 (86.9)	12080 (82.8)	3489 (78.0)	5991 (77.9)	2087 (68.1)	3911 (79.2)	1040 (84.2)	4311 (66.8)
	Yes	2650 (11.5)	2489 (17.1)	978 (21.9)	1698 (22.1)	973 (31.8)	1026 (20.8)	195 (15.8)	2141 (33.2)
	(Missing)	379 (1.6)	14 (0.1)	5 (0.1)	4 (0.1)	4 (0.1)	1 (0.0)	0 (0.0)	6 (0.1)
Any non-invasive ventilation	No	17685 (79.3)	10947 (76.1)	3311 (74.8)	5623 (73.9)	2117 (70.0)	3678 (75.1)	1000 (81.8)	4100 (64.2)
	Yes	4621 (20.7)	3445 (23.9)	1113 (25.2)	1987 (26.1)	906 (30.0)	1217 (24.9)	222 (18.2)	2290 (35.8)
Any oxygen	No	2145 (9.5)	1079 (7.4)	331 (7.4)	528 (6.9)	213 (7.0)	302 (6.1)	157 (12.8)	211 (3.3)
	Yes	20385 (90.5)	13421 (92.6)	4120 (92.6)	7128 (93.1)	2832 (93.0)	4624 (93.9)	1073 (87.2)	6219 (96.7)

Percentage values are row percentages, except for totals which represent column percentages. § = Denominator is number of non-missing observations for each organ system.

Supplementary table 8 - Effect of complications on survival (adjusted Cox proportional hazards models) for figure 2C

Supplementary table 8A - Any complication

Dependent: Survival (mortality)			HR (univariable)	HR (multivariable)
Any complication	No	37839 (100.0)	-	-
	Yes	36662 (100.0)	1.91 (1.86-1.97, p<0.001)	1.74 (1.64-1.84, p<0.001)
Age on admission	19-29	1477 (100.0)	-	-
	30-39	2707 (100.0)	1.82 (1.25-2.64, p=0.002)	1.68 (1.22-2.31, p=0.002)
	40-49	4930 (100.0)	3.22 (2.29-4.53, p<0.001)	2.78 (1.97-3.94, p<0.001)
	50-59	9003 (100.0)	6.24 (4.48-8.68, p<0.001)	5.26 (3.79-7.29, p<0.001)
	60-69	11035 (100.0)	11.72 (8.43-16.27, p<0.001)	9.68 (6.99-13.40, p<0.001)
	70-79	16445 (100.0)	18.38 (13.25-25.51, p<0.001)	15.44 (11.07-21.54, p<0.001)
	80-89	19771 (100.0)	23.49 (16.93-32.59, p<0.001)	20.15 (14.56-27.89, p<0.001)
	90+	7204 (100.0)	27.63 (19.89-38.36, p<0.001)	24.73 (17.77-34.40, p<0.001)
Sex at Birth	Female	31733 (100.0)	-	-
	Male	40648 (100.0)	1.23 (1.20-1.27, p<0.001)	1.30 (1.26-1.33, p<0.001)
Deprivation	1 (least deprived)	10347 (100.0)	-	-
	2	12775 (100.0)	1.00 (0.96-1.05, p=0.965)	1.03 (0.97-1.08, p=0.348)
	3	15712 (100.0)	0.99 (0.95-1.04, p=0.743)	1.06 (0.99-1.12, p=0.074)
	4	15880 (100.0)	0.95 (0.91-1.00, p=0.039)	1.07 (1.01-1.13, p=0.033)
	5 (most deprived)	17845 (100.0)	0.93 (0.89-0.97, p=0.001)	1.09 (1.03-1.15, p=0.002)

Supplementary table 8B - Systemic complications

Dependent: Survival (mortality)			HR (univariable)	HR (multivariable)
Systemic Complication	No	62531 (100.0)	-	-
	Yes	11970 (100.0)	1.27 (1.23-1.31, p<0.001)	1.20 (1.15-1.26, p<0.001)
Age on admission	19-29	1477 (100.0)	-	-
	30-39	2707 (100.0)	1.82 (1.25-2.64, p=0.002)	1.75 (1.27-2.42, p=0.001)
	40-49	4930 (100.0)	3.22 (2.29-4.53, p<0.001)	3.00 (2.12-4.26, p<0.001)
	50-59	9003 (100.0)	6.24 (4.48-8.68, p<0.001)	5.79 (4.17-8.04, p<0.001)
	60-69	11035 (100.0)	11.72 (8.43-16.27, p<0.001)	10.87 (7.84-15.07, p<0.001)
	70-79	16445 (100.0)	18.38 (13.25-25.51, p<0.001)	17.25 (12.36-24.09, p<0.001)
	80-89	19771 (100.0)	23.49 (16.93-32.59, p<0.001)	22.50 (16.24-31.18, p<0.001)
	90+	7204 (100.0)	27.63 (19.89-38.36, p<0.001)	27.61 (19.83-38.43, p<0.001)
Sex at Birth	Female	31733 (100.0)	-	-
	Male	40648 (100.0)	1.23 (1.20-1.27, p<0.001)	1.34 (1.30-1.38, p<0.001)
Deprivation	1 (least deprived)	10347 (100.0)	-	-
	2	12775 (100.0)	1.00 (0.96-1.05, p=0.965)	1.03 (0.98-1.08, p=0.281)
	3	15712 (100.0)	0.99 (0.95-1.04, p=0.743)	1.06 (1.00-1.12, p=0.059)
	4	15880 (100.0)	0.95 (0.91-1.00, p=0.039)	1.07 (1.02-1.13, p=0.012)
	5 (most deprived)	17845 (100.0)	0.93 (0.89-0.97, p=0.001)	1.09 (1.04-1.15, p=0.001)

Supplementary table 8C - Renal complications/ acute kidney injury

Dependent: Survival (mortality)			HR (univariable)	HR (multivariable)
Renal complication	No	56691 (100.0)	-	-
	Yes	17810 (100.0)	1.68 (1.64-1.73, p<0.001)	1.49 (1.42-1.55, p<0.001)
Age on admission	19-29	1477 (100.0)	-	-
	30-39	2707 (100.0)	1.82 (1.25-2.64, p=0.002)	1.74 (1.26-2.39, p=0.001)
	40-49	4930 (100.0)	3.22 (2.29-4.53, p<0.001)	2.92 (2.06-4.14, p<0.001)
	50-59	9003 (100.0)	6.24 (4.48-8.68, p<0.001)	5.53 (3.99-7.68, p<0.001)
	60-69	11035 (100.0)	11.72 (8.43-16.27, p<0.001)	10.22 (7.37-14.17, p<0.001)
	70-79	16445 (100.0)	18.38 (13.25-25.51, p<0.001)	16.24 (11.63-22.67, p<0.001)
	80-89	19771 (100.0)	23.49 (16.93-32.59, p<0.001)	21.16 (15.27-29.32, p<0.001)
	90+	7204 (100.0)	27.63 (19.89-38.36, p<0.001)	26.05 (18.70-36.27, p<0.001)
Sex at Birth	Female	31733 (100.0)	-	-
	Male	40648 (100.0)	1.23 (1.20-1.27, p<0.001)	1.31 (1.27-1.35, p<0.001)
Deprivation	1 (least deprived)	10347 (100.0)	-	-
	2	12775 (100.0)	1.00 (0.96-1.05, p=0.965)	1.03 (0.98-1.08, p=0.317)
	3	15712 (100.0)	0.99 (0.95-1.04, p=0.743)	1.05 (0.99-1.11, p=0.089)
	4	15880 (100.0)	0.95 (0.91-1.00, p=0.039)	1.06 (1.00-1.12, p=0.046)
	5 (most deprived)	17845 (100.0)	0.93 (0.89-0.97, p=0.001)	1.08 (1.02-1.13, p=0.006)

Supplementary table 8D - Gastrointestinal and liver complications

Dependent: Survival (mortality)			HR (univariable)	HR (multivariable)
Gastrointestinal or liver complication	No	66464 (100.0)	-	-
	Yes	8037 (100.0)	1.28 (1.23-1.33, p<0.001)	1.50 (1.44-1.57, p<0.001)
Age on admission	19-29	1477 (100.0)	-	-
	30-39	2707 (100.0)	1.82 (1.25-2.64, p=0.002)	1.74 (1.26-2.39, p=0.001)
	40-49	4930 (100.0)	3.22 (2.29-4.53, p<0.001)	2.97 (2.10-4.20, p<0.001)
	50-59	9003 (100.0)	6.24 (4.48-8.68, p<0.001)	5.71 (4.12-7.92, p<0.001)
	60-69	11035 (100.0)	11.72 (8.43-16.27, p<0.001)	10.85 (7.84-15.02, p<0.001)
	70-79	16445 (100.0)	18.38 (13.25-25.51, p<0.001)	17.54 (12.58-24.45, p<0.001)
	80-89	19771 (100.0)	23.49 (16.93-32.59, p<0.001)	23.15 (16.73-32.03, p<0.001)
	90+	7204 (100.0)	27.63 (19.89-38.36, p<0.001)	28.46 (20.46-39.59, p<0.001)
Sex at Birth	Female	31733 (100.0)	-	-
	Male	40648 (100.0)	1.23 (1.20-1.27, p<0.001)	1.33 (1.29-1.37, p<0.001)
Deprivation	1 (least deprived)	10347 (100.0)	-	-
	2	12775 (100.0)	1.00 (0.96-1.05, p=0.965)	1.03 (0.98-1.08, p=0.292)
	3	15712 (100.0)	0.99 (0.95-1.04, p=0.743)	1.06 (1.00-1.12, p=0.052)
	4	15880 (100.0)	0.95 (0.91-1.00, p=0.039)	1.08 (1.02-1.14, p=0.011)
	5 (most deprived)	17845 (100.0)	0.93 (0.89-0.97, p=0.001)	1.09 (1.04-1.15, p=0.001)

Supplementary table 8E - Cardiovascular complications

Dependent: Survival (mortality)			HR (univariable)	HR (multivariable)
Cardiovascular complication	No	65498 (100.0)	-	-
	Yes	9003 (100.0)	2.32 (2.25-2.39, p<0.001)	1.98 (1.85-2.11, p<0.001)
Age on admission	19-29	1477 (100.0)	-	-
	30-39	2707 (100.0)	1.82 (1.25-2.64, p=0.002)	1.74 (1.26-2.39, p=0.001)
	40-49	4930 (100.0)	3.22 (2.29-4.53, p<0.001)	2.93 (2.07-4.15, p<0.001)
	50-59	9003 (100.0)	6.24 (4.48-8.68, p<0.001)	5.57 (4.02-7.73, p<0.001)
	60-69	11035 (100.0)	11.72 (8.43-16.27, p<0.001)	10.23 (7.38-14.17, p<0.001)
	70-79	16445 (100.0)	18.38 (13.25-25.51, p<0.001)	16.11 (11.55-22.48, p<0.001)
	80-89	19771 (100.0)	23.49 (16.93-32.59, p<0.001)	20.85 (15.05-28.88, p<0.001)
	90+	7204 (100.0)	27.63 (19.89-38.36, p<0.001)	25.50 (18.34-35.45, p<0.001)
Sex at Birth	Female	31733 (100.0)	-	-
	Male	40648 (100.0)	1.23 (1.20-1.27, p<0.001)	1.32 (1.28-1.36, p<0.001)
Deprivation	1 (least deprived)	10347 (100.0)	-	-
	2	12775 (100.0)	1.00 (0.96-1.05, p=0.965)	1.03 (0.98-1.09, p=0.219)
	3	15712 (100.0)	0.99 (0.95-1.04, p=0.743)	1.06 (1.00-1.12, p=0.053)
	4	15880 (100.0)	0.95 (0.91-1.00, p=0.039)	1.07 (1.02-1.14, p=0.012)
	5 (most deprived)	17845 (100.0)	0.93 (0.89-0.97, p=0.001)	1.10 (1.05-1.16, p<0.001)

Supplementary table 8F - Neurological complications

Dependent: Survival (mortality)			HR (univariable)	HR (multivariable)
Neurological complication	No	71340 (100.0)	-	-
	Yes	3161 (100.0)	1.29 (1.22-1.37, p<0.001)	1.21 (1.13-1.29, p<0.001)
Age on admission	19-29	1477 (100.0)	-	-
	30-39	2707 (100.0)	1.82 (1.25-2.64, p=0.002)	1.76 (1.28-2.43, p=0.001)
	40-49	4930 (100.0)	3.22 (2.29-4.53, p<0.001)	3.03 (2.13-4.29, p<0.001)
	50-59	9003 (100.0)	6.24 (4.48-8.68, p<0.001)	5.84 (4.21-8.11, p<0.001)
	60-69	11035 (100.0)	11.72 (8.43-16.27, p<0.001)	10.99 (7.93-15.24, p<0.001)
	70-79	16445 (100.0)	18.38 (13.25-25.51, p<0.001)	17.42 (12.48-24.31, p<0.001)
	80-89	19771 (100.0)	23.49 (16.93-32.59, p<0.001)	22.70 (16.38-31.44, p<0.001)
	90+	7204 (100.0)	27.63 (19.89-38.36, p<0.001)	27.85 (20.00-38.77, p<0.001)
Sex at Birth	Female	31733 (100.0)	-	-
	Male	40648 (100.0)	1.23 (1.20-1.27, p<0.001)	1.34 (1.31-1.38, p<0.001)
Deprivation	1 (least deprived)	10347 (100.0)	-	-
	2	12775 (100.0)	1.00 (0.96-1.05, p=0.965)	1.03 (0.98-1.08, p=0.287)
	3	15712 (100.0)	0.99 (0.95-1.04, p=0.743)	1.06 (1.00-1.12, p=0.060)
	4	15880 (100.0)	0.95 (0.91-1.00, p=0.039)	1.07 (1.01-1.13, p=0.014)
	5 (most deprived)	17845 (100.0)	0.93 (0.89-0.97, p=0.001)	1.09 (1.03-1.14, p=0.001)

Supplementary table 8G - Respiratory complications

Dependent: Survival (mortality)			HR (univariable)	HR (multivariable)
Respiratory complication	No	60982 (100.0)	-	-
	Yes	13519 (100.0)	1.89 (1.83-1.94, p<0.001)	2.15 (2.04-2.27, p<0.001)
Age on admission	19-29	1477 (100.0)	-	-
	30-39	2707 (100.0)	1.82 (1.25-2.64, p=0.002)	1.65 (1.20-2.27, p=0.002)
	40-49	4930 (100.0)	3.22 (2.29-4.53, p<0.001)	2.67 (1.89-3.78, p<0.001)
	50-59	9003 (100.0)	6.24 (4.48-8.68, p<0.001)	5.12 (3.70-7.10, p<0.001)
	60-69	11035 (100.0)	11.72 (8.43-16.27, p<0.001)	9.71 (7.01-13.45, p<0.001)
	70-79	16445 (100.0)	18.38 (13.25-25.51, p<0.001)	16.45 (11.80-22.92, p<0.001)
	80-89	19771 (100.0)	23.49 (16.93-32.59, p<0.001)	22.33 (16.15-30.89, p<0.001)
	90+	7204 (100.0)	27.63 (19.89-38.36, p<0.001)	27.49 (19.78-38.21, p<0.001)
Sex at Birth	Female	31733 (100.0)	-	-
	Male	40648 (100.0)	1.23 (1.20-1.27, p<0.001)	1.31 (1.27-1.35, p<0.001)
Deprivation	1 (least deprived)	10347 (100.0)	-	-
	2	12775 (100.0)	1.00 (0.96-1.05, p=0.965)	1.03 (0.98-1.08, p=0.209)
	3	15712 (100.0)	0.99 (0.95-1.04, p=0.743)	1.05 (0.99-1.11, p=0.135)
	4	15880 (100.0)	0.95 (0.91-1.00, p=0.039)	1.07 (1.01-1.13, p=0.022)
	5 (most deprived)	17845 (100.0)	0.93 (0.89-0.97, p=0.001)	1.10 (1.04-1.16, p<0.001)

Supplementary table 9 - Effect of complications on odds of critical care admission (adjusted logistic regression models) for figure 2D

Supplementary table 9A - Any complication

Dependent: Critical Care Admission		No	Yes	OR (univariable)	OR (multilevel)
Any complication	No	33809 (95.1)	1760 (4.9)	-	-
	Yes	27937 (77.4)	8155 (22.6)	5.61 (5.31-5.92, p<0.001)	7.25 (6.83-7.69, p<0.001)
Age on admission	19-29	1241 (85.8)	205 (14.2)	-	-
	30-39	2101 (79.1)	555 (20.9)	1.60 (1.34-1.91, p<0.001)	1.33 (1.10-1.61, p=0.004)
	40-49	3596 (74.0)	1264 (26.0)	2.13 (1.82-2.51, p<0.001)	1.57 (1.32-1.88, p<0.001)
	50-59	6389 (71.7)	2521 (28.3)	2.39 (2.05-2.80, p<0.001)	1.63 (1.38-1.94, p<0.001)
	60-69	8166 (74.9)	2740 (25.1)	2.03 (1.75-2.38, p<0.001)	1.24 (1.04-1.47, p=0.014)
	70-79	14285 (87.9)	1960 (12.1)	0.83 (0.71-0.97, p=0.019)	0.47 (0.39-0.56, p<0.001)
	80-89	18964 (97.1)	567 (2.9)	0.18 (0.15-0.21, p<0.001)	0.10 (0.08-0.12, p<0.001)
	90+	7004 (98.6)	103 (1.4)	0.09 (0.07-0.11, p<0.001)	0.05 (0.04-0.07, p<0.001)
Sex at Birth	Female	28230 (90.1)	3100 (9.9)	-	-
	Male	33348 (83.1)	6792 (16.9)	1.85 (1.77-1.94, p<0.001)	1.49 (1.42-1.57, p<0.001)
Deprivation	1 (least deprived)	8747 (86.2)	1401 (13.8)	-	-
	2	10923 (86.9)	1653 (13.1)	0.94 (0.88-1.02, p=0.146)	0.86 (0.79-0.94, p=0.001)
	3	13352 (86.3)	2115 (13.7)	0.99 (0.92-1.06, p=0.765)	0.89 (0.82-0.98, p=0.012)
	4	13360 (85.1)	2340 (14.9)	1.09 (1.02-1.17, p=0.014)	0.89 (0.82-0.97, p=0.011)
	5 (most deprived)	15352 (86.5)	2405 (13.5)	0.98 (0.91-1.05, p=0.540)	0.78 (0.72-0.85, p<0.001)

Supplementary table 9B - Systemic complications

Dependent: Critical Care Admission		No	Yes	OR (univariable)	OR (multilevel)
Systemic complication	No	53004 (88.5)	6875 (11.5)	-	-
	Yes	8742 (74.2)	3040 (25.8)	2.68 (2.55-2.81, p<0.001)	3.15 (2.97-3.33, p<0.001)
Age on admission	19-29	1241 (85.8)	205 (14.2)	-	-
	30-39	2101 (79.1)	555 (20.9)	1.60 (1.34-1.91, p<0.001)	1.45 (1.21-1.74, p<0.001)
	40-49	3596 (74.0)	1264 (26.0)	2.13 (1.82-2.51, p<0.001)	1.84 (1.56-2.18, p<0.001)
	50-59	6389 (71.7)	2521 (28.3)	2.39 (2.05-2.80, p<0.001)	2.05 (1.74-2.41, p<0.001)
	60-69	8166 (74.9)	2740 (25.1)	2.03 (1.75-2.38, p<0.001)	1.70 (1.45-2.00, p<0.001)
	70-79	14285 (87.9)	1960 (12.1)	0.83 (0.71-0.97, p=0.019)	0.69 (0.58-0.81, p<0.001)
	80-89	18964 (97.1)	567 (2.9)	0.18 (0.15-0.21, p<0.001)	0.15 (0.13-0.18, p<0.001)
	90+	7004 (98.6)	103 (1.4)	0.09 (0.07-0.11, p<0.001)	0.08 (0.06-0.10, p<0.001)
Sex at Birth	Female	28230 (90.1)	3100 (9.9)	-	-
	Male	33348 (83.1)	6792 (16.9)	1.85 (1.77-1.94, p<0.001)	1.65 (1.58-1.74, p<0.001)
Deprivation	1 (least deprived)	8747 (86.2)	1401 (13.8)	-	-
	2	10923 (86.9)	1653 (13.1)	0.94 (0.88-1.02, p=0.146)	0.88 (0.81-0.96, p=0.005)
	3	13352 (86.3)	2115 (13.7)	0.99 (0.92-1.06, p=0.765)	0.90 (0.83-0.98, p=0.020)
	4	13360 (85.1)	2340 (14.9)	1.09 (1.02-1.17, p=0.014)	0.91 (0.84-0.99, p=0.035)
	5 (most deprived)	15352 (86.5)	2405 (13.5)	0.98 (0.91-1.05, p=0.540)	0.82 (0.75-0.89, p<0.001)

Supplementary table 9C - Renal complications/ acute kidney injury

Dependent: Critical Care Admission		No	Yes	OR (univariable)	OR (multilevel)
Renal complication	No	48812 (90.3)	5238 (9.7)	-	-
	Yes	12934 (73.4)	4677 (26.6)	3.37 (3.22-3.52, p<0.001)	4.36 (4.14-4.58, p<0.001)
Age on admission	19-29	1241 (85.8)	205 (14.2)	-	-
	30-39	2101 (79.1)	555 (20.9)	1.60 (1.34-1.91, p<0.001)	1.45 (1.21-1.74, p<0.001)
	40-49	3596 (74.0)	1264 (26.0)	2.13 (1.82-2.51, p<0.001)	1.74 (1.47-2.06, p<0.001)
	50-59	6389 (71.7)	2521 (28.3)	2.39 (2.05-2.80, p<0.001)	1.82 (1.54-2.14, p<0.001)
	60-69	8166 (74.9)	2740 (25.1)	2.03 (1.75-2.38, p<0.001)	1.40 (1.19-1.65, p<0.001)
	70-79	14285 (87.9)	1960 (12.1)	0.83 (0.71-0.97, p=0.019)	0.54 (0.46-0.64, p<0.001)
	80-89	18964 (97.1)	567 (2.9)	0.18 (0.15-0.21, p<0.001)	0.11 (0.10-0.14, p<0.001)
	90+	7004 (98.6)	103 (1.4)	0.09 (0.07-0.11, p<0.001)	0.06 (0.05-0.07, p<0.001)
Sex at Birth	Female	28230 (90.1)	3100 (9.9)	-	-
	Male	33348 (83.1)	6792 (16.9)	1.85 (1.77-1.94, p<0.001)	1.51 (1.44-1.59, p<0.001)
Deprivation	1 (least deprived)	8747 (86.2)	1401 (13.8)	-	-
	2	10923 (86.9)	1653 (13.1)	0.94 (0.88-1.02, p=0.146)	0.89 (0.81-0.97, p=0.009)
	3	13352 (86.3)	2115 (13.7)	0.99 (0.92-1.06, p=0.765)	0.89 (0.82-0.97, p=0.011)
	4	13360 (85.1)	2340 (14.9)	1.09 (1.02-1.17, p=0.014)	0.89 (0.81-0.97, p=0.006)
	5 (most deprived)	15352 (86.5)	2405 (13.5)	0.98 (0.91-1.05, p=0.540)	0.79 (0.73-0.86, p<0.001)

Supplementary table 9D - Gastrointestinal and liver complications

Dependent: Critical Care Admission		No	Yes	OR (univariable)	OR (multilevel)
Gastrointestinal or liver complication	No	56625 (88.8)	7173 (11.2)	-	-
	Yes	5121 (65.1)	2742 (34.9)	4.23 (4.01-4.45, p<0.001)	3.52 (3.32-3.74, p<0.001)
Age on admission	19-29	1241 (85.8)	205 (14.2)	-	-
	30-39	2101 (79.1)	555 (20.9)	1.60 (1.34-1.91, p<0.001)	1.45 (1.21-1.75, p<0.001)
	40-49	3596 (74.0)	1264 (26.0)	2.13 (1.82-2.51, p<0.001)	1.84 (1.56-2.19, p<0.001)
	50-59	6389 (71.7)	2521 (28.3)	2.39 (2.05-2.80, p<0.001)	2.08 (1.77-2.45, p<0.001)
	60-69	8166 (74.9)	2740 (25.1)	2.03 (1.75-2.38, p<0.001)	1.82 (1.55-2.14, p<0.001)
	70-79	14285 (87.9)	1960 (12.1)	0.83 (0.71-0.97, p=0.019)	0.77 (0.65-0.90, p=0.001)
	80-89	18964 (97.1)	567 (2.9)	0.18 (0.15-0.21, p<0.001)	0.18 (0.15-0.21, p<0.001)
	90+	7004 (98.6)	103 (1.4)	0.09 (0.07-0.11, p<0.001)	0.09 (0.07-0.12, p<0.001)
Sex at Birth	Female	28230 (90.1)	3100 (9.9)	-	-
	Male	33348 (83.1)	6792 (16.9)	1.85 (1.77-1.94, p<0.001)	1.56 (1.48-1.63, p<0.001)
Deprivation	1 (least deprived)	8747 (86.2)	1401 (13.8)	-	-
	2	10923 (86.9)	1653 (13.1)	0.94 (0.88-1.02, p=0.146)	0.89 (0.82-0.97, p=0.009)
	3	13352 (86.3)	2115 (13.7)	0.99 (0.92-1.06, p=0.765)	0.93 (0.86-1.01, p=0.096)
	4	13360 (85.1)	2340 (14.9)	1.09 (1.02-1.17, p=0.014)	0.94 (0.87-1.03, p=0.170)
	5 (most deprived)	15352 (86.5)	2405 (13.5)	0.98 (0.91-1.05, p=0.540)	0.85 (0.78-0.92, p<0.001)

Supplementary table 9E - Cardiovascular complications

Dependent: Critical Care Admission		No	Yes	OR (univariable)	OR (multilevel)
Cardiovascular complication	No	55139 (87.9)	7619 (12.1)	-	-
	Yes	6607 (74.2)	2296 (25.8)	2.51 (2.38-2.65, p<0.001)	3.64 (3.42-3.88, p<0.001)
Age on admission	19-29	1241 (85.8)	205 (14.2)	-	-
	30-39	2101 (79.1)	555 (20.9)	1.60 (1.34-1.91, p<0.001)	1.48 (1.24-1.78, p<0.001)
	40-49	3596 (74.0)	1264 (26.0)	2.13 (1.82-2.51, p<0.001)	1.83 (1.54-2.16, p<0.001)
	50-59	6389 (71.7)	2521 (28.3)	2.39 (2.05-2.80, p<0.001)	2.02 (1.72-2.37, p<0.001)
	60-69	8166 (74.9)	2740 (25.1)	2.03 (1.75-2.38, p<0.001)	1.63 (1.39-1.91, p<0.001)
	70-79	14285 (87.9)	1960 (12.1)	0.83 (0.71-0.97, p=0.019)	0.63 (0.53-0.74, p<0.001)
	80-89	18964 (97.1)	567 (2.9)	0.18 (0.15-0.21, p<0.001)	0.13 (0.11-0.16, p<0.001)
	90+	7004 (98.6)	103 (1.4)	0.09 (0.07-0.11, p<0.001)	0.07 (0.05-0.09, p<0.001)
Sex at Birth	Female	28230 (90.1)	3100 (9.9)	-	-
	Male	33348 (83.1)	6792 (16.9)	1.85 (1.77-1.94, p<0.001)	1.61 (1.53-1.69, p<0.001)
Deprivation	1 (least deprived)	8747 (86.2)	1401 (13.8)	-	-
	2	10923 (86.9)	1653 (13.1)	0.94 (0.88-1.02, p=0.146)	0.89 (0.81-0.97, p=0.006)
	3	13352 (86.3)	2115 (13.7)	0.99 (0.92-1.06, p=0.765)	0.92 (0.84-1.00, p=0.046)
	4	13360 (85.1)	2340 (14.9)	1.09 (1.02-1.17, p=0.014)	0.92 (0.85-1.00, p=0.050)
	5 (most deprived)	15352 (86.5)	2405 (13.5)	0.98 (0.91-1.05, p=0.540)	0.83 (0.76-0.90, p<0.001)

Supplementary table 9F - Neurological complications

Dependent: Critical Care Admission		No	Yes	OR (univariable)	OR (multilevel)
Neurological complication	No	59321 (86.5)	9264 (13.5)	-	-
	Yes	2425 (78.8)	651 (21.2)	1.72 (1.57-1.88, p<0.001)	1.88 (1.70-2.08, p<0.001)
Age on admission	19-29	1241 (85.8)	205 (14.2)	-	-
	30-39	2101 (79.1)	555 (20.9)	1.60 (1.34-1.91, p<0.001)	1.51 (1.26-1.81, p<0.001)
	40-49	3596 (74.0)	1264 (26.0)	2.13 (1.82-2.51, p<0.001)	1.91 (1.62-2.26, p<0.001)
	50-59	6389 (71.7)	2521 (28.3)	2.39 (2.05-2.80, p<0.001)	2.17 (1.85-2.55, p<0.001)
	60-69	8166 (74.9)	2740 (25.1)	2.03 (1.75-2.38, p<0.001)	1.85 (1.58-2.17, p<0.001)
	70-79	14285 (87.9)	1960 (12.1)	0.83 (0.71-0.97, p=0.019)	0.75 (0.64-0.88, p<0.001)
	80-89	18964 (97.1)	567 (2.9)	0.18 (0.15-0.21, p<0.001)	0.17 (0.14-0.20, p<0.001)
	90+	7004 (98.6)	103 (1.4)	0.09 (0.07-0.11, p<0.001)	0.09 (0.07-0.11, p<0.001)
Sex at Birth	Female	28230 (90.1)	3100 (9.9)	-	-
	Male	33348 (83.1)	6792 (16.9)	1.85 (1.77-1.94, p<0.001)	1.65 (1.57-1.73, p<0.001)
Deprivation	1 (least deprived)	8747 (86.2)	1401 (13.8)	-	-
	2	10923 (86.9)	1653 (13.1)	0.94 (0.88-1.02, p=0.146)	0.90 (0.82-0.97, p=0.010)
	3	13352 (86.3)	2115 (13.7)	0.99 (0.92-1.06, p=0.765)	0.92 (0.85-1.00, p=0.065)
	4	13360 (85.1)	2340 (14.9)	1.09 (1.02-1.17, p=0.014)	0.94 (0.86-1.02, p=0.114)
	5 (most deprived)	15352 (86.5)	2405 (13.5)	0.98 (0.91-1.05, p=0.540)	0.83 (0.77-0.91, p<0.001)

Supplementary table 9G - Respiratory complications

Dependent: Critical Care Admission		No	Yes	OR (univariable)	OR (multilevel)
Respiratory complication	No	54321 (93.2)	3986 (6.8)	-	-
	Yes	7425 (55.6)	5929 (44.4)	10.88 (10.38-11.41, p<0.001)	12.48 (11.81-13.18, p<0.001)
Age on admission	19-29	1241 (85.8)	205 (14.2)	-	-
	30-39	2101 (79.1)	555 (20.9)	1.60 (1.34-1.91, p<0.001)	1.28 (1.05-1.56, p=0.015)
	40-49	3596 (74.0)	1264 (26.0)	2.13 (1.82-2.51, p<0.001)	1.40 (1.17-1.68, p<0.001)
	50-59	6389 (71.7)	2521 (28.3)	2.39 (2.05-2.80, p<0.001)	1.56 (1.31-1.86, p<0.001)
	60-69	8166 (74.9)	2740 (25.1)	2.03 (1.75-2.38, p<0.001)	1.27 (1.06-1.51, p=0.008)
	70-79	14285 (87.9)	1960 (12.1)	0.83 (0.71-0.97, p=0.019)	0.52 (0.44-0.62, p<0.001)
	80-89	18964 (97.1)	567 (2.9)	0.18 (0.15-0.21, p<0.001)	0.11 (0.09-0.14, p<0.001)
	90+	7004 (98.6)	103 (1.4)	0.09 (0.07-0.11, p<0.001)	0.06 (0.04-0.08, p<0.001)
Sex at Birth	Female	28230 (90.1)	3100 (9.9)	-	-
	Male	33348 (83.1)	6792 (16.9)	1.85 (1.77-1.94, p<0.001)	1.53 (1.44-1.61, p<0.001)
Deprivation	1 (least deprived)	8747 (86.2)	1401 (13.8)	-	-
	2	10923 (86.9)	1653 (13.1)	0.94 (0.88-1.02, p=0.146)	0.86 (0.79-0.95, p=0.003)
	3	13352 (86.3)	2115 (13.7)	0.99 (0.92-1.06, p=0.765)	0.90 (0.82-0.99, p=0.029)
	4	13360 (85.1)	2340 (14.9)	1.09 (1.02-1.17, p=0.014)	0.88 (0.81-0.97, p=0.009)
	5 (most deprived)	15352 (86.5)	2405 (13.5)	0.98 (0.91-1.05, p=0.540)	0.80 (0.73-0.88, p<0.001)

Supplementary table 10 - Effect of complications on odds of worse ability to self-care at discharge (adjusted logistic regression models) for figure 4C

Supplementary table 10A - Any complication

Dependent: Self-care ability		Equivalent to before illness	Worse	OR (univariable)	OR (multilevel)
Any complication	No	19692 (77.4)	5759 (22.6)	-	-
	Yes	12174 (61.7)	7550 (38.3)	2.12 (2.04-2.21, p<0.001)	2.41 (2.30-2.52, p<0.001)
Age (years)	19-29	1121 (86.7)	172 (13.3)	-	-
	30-39	1962 (83.7)	383 (16.3)	1.27 (1.05-1.55, p=0.015)	1.54 (1.24-1.92, p<0.001)
	40-49	3331 (79.9)	838 (20.1)	1.64 (1.38-1.96, p<0.001)	2.03 (1.66-2.48, p<0.001)
	50-59	5508 (78.8)	1486 (21.2)	1.76 (1.49-2.09, p<0.001)	2.22 (1.82-2.69, p<0.001)
	60-69	5451 (73.3)	1990 (26.7)	2.38 (2.02-2.82, p<0.001)	3.02 (2.49-3.66, p<0.001)
	70-79	6237 (66.5)	3139 (33.5)	3.28 (2.79-3.88, p<0.001)	4.18 (3.46-5.06, p<0.001)
	80-89	6069 (61.4)	3810 (38.6)	4.09 (3.48-4.84, p<0.001)	5.38 (4.45-6.50, p<0.001)
	90+	1940 (58.5)	1377 (41.5)	4.63 (3.89-5.52, p<0.001)	6.26 (5.13-7.65, p<0.001)
Sex at Birth	Female	14550 (70.9)	5968 (29.1)	-	-
	Male	17230 (70.2)	7308 (29.8)	1.03 (0.99-1.08, p=0.107)	1.05 (1.01-1.10, p=0.021)
Deprivation	1 (least deprived)	4468 (69.7)	1946 (30.3)	-	-
	2	5473 (70.0)	2345 (30.0)	0.98 (0.92-1.06, p=0.655)	0.97 (0.90-1.05, p=0.442)
	3	6751 (69.4)	2983 (30.6)	1.01 (0.95-1.09, p=0.680)	1.03 (0.95-1.12, p=0.438)
	4	7008 (70.2)	2982 (29.8)	0.98 (0.91-1.05, p=0.504)	1.07 (0.99-1.16, p=0.095)
	5 (most deprived)	8156 (72.8)	3052 (27.2)	0.86 (0.80-0.92, p<0.001)	1.03 (0.95-1.11, p=0.545)

Supplementary table 10B - Systemic complications

Dependent: Self-care ability		Equivalent to before illness	Worse	OR (univariable)	OR (multilevel)
Systemic complication	No	26339 (73.7)	9378 (26.3)	-	-
	Yes	3812 (56.1)	2987 (43.9)	2.20 (2.09-2.32, p<0.001)	2.39 (2.25-2.53, p<0.001)
Age (years)	19-29	1121 (86.7)	172 (13.3)	-	-
	30-39	1962 (83.7)	383 (16.3)	1.27 (1.05-1.55, p=0.015)	1.66 (1.33-2.09, p<0.001)
	40-49	3331 (79.9)	838 (20.1)	1.64 (1.38-1.96, p<0.001)	2.28 (1.85-2.82, p<0.001)
	50-59	5508 (78.8)	1486 (21.2)	1.76 (1.49-2.09, p<0.001)	2.46 (2.01-3.02, p<0.001)
	60-69	5451 (73.3)	1990 (26.7)	2.38 (2.02-2.82, p<0.001)	3.36 (2.74-4.11, p<0.001)
	70-79	6237 (66.5)	3139 (33.5)	3.28 (2.79-3.88, p<0.001)	4.66 (3.82-5.69, p<0.001)
	80-89	6069 (61.4)	3810 (38.6)	4.09 (3.48-4.84, p<0.001)	6.03 (4.94-7.36, p<0.001)
	90+	1940 (58.5)	1377 (41.5)	4.63 (3.89-5.52, p<0.001)	6.99 (5.68-8.62, p<0.001)
Sex at Birth	Female	14550 (70.9)	5968 (29.1)	-	-
	Male	17230 (70.2)	7308 (29.8)	1.03 (0.99-1.08, p=0.107)	1.10 (1.05-1.15, p<0.001)
Deprivation	1 (least deprived)	4468 (69.7)	1946 (30.3)	-	-
	2	5473 (70.0)	2345 (30.0)	0.98 (0.92-1.06, p=0.655)	0.98 (0.91-1.07, p=0.687)
	3	6751 (69.4)	2983 (30.6)	1.01 (0.95-1.09, p=0.680)	1.03 (0.95-1.12, p=0.412)
	4	7008 (70.2)	2982 (29.8)	0.98 (0.91-1.05, p=0.504)	1.09 (1.01-1.19, p=0.031)
	5 (most deprived)	8156 (72.8)	3052 (27.2)	0.86 (0.80-0.92, p<0.001)	1.03 (0.95-1.12, p=0.429)

Supplementary table 10C - Renal complications/ acute kidney injury

Dependent: Self-care ability		Equivalent to before illness	Worse	OR (univariable)	OR (multilevel)
Renal complication	No	25847 (74.1)	9039 (25.9)	-	-
	Yes	5287 (58.2)	3791 (41.8)	2.05 (1.95-2.15, p<0.001)	2.12 (2.01-2.23, p<0.001)
Age (years)	19-29	1121 (86.7)	172 (13.3)	-	-
	30-39	1962 (83.7)	383 (16.3)	1.27 (1.05-1.55, p=0.015)	1.67 (1.34-2.10, p<0.001)
	40-49	3331 (79.9)	838 (20.1)	1.64 (1.38-1.96, p<0.001)	2.25 (1.83-2.77, p<0.001)
	50-59	5508 (78.8)	1486 (21.2)	1.76 (1.49-2.09, p<0.001)	2.38 (1.95-2.91, p<0.001)
	60-69	5451 (73.3)	1990 (26.7)	2.38 (2.02-2.82, p<0.001)	3.27 (2.68-3.99, p<0.001)
	70-79	6237 (66.5)	3139 (33.5)	3.28 (2.79-3.88, p<0.001)	4.45 (3.66-5.43, p<0.001)
	80-89	6069 (61.4)	3810 (38.6)	4.09 (3.48-4.84, p<0.001)	5.72 (4.70-6.97, p<0.001)
	90+	1940 (58.5)	1377 (41.5)	4.63 (3.89-5.52, p<0.001)	6.61 (5.38-8.13, p<0.001)
Sex at Birth	Female	14550 (70.9)	5968 (29.1)	-	-
	Male	17230 (70.2)	7308 (29.8)	1.03 (0.99-1.08, p=0.107)	1.06 (1.01-1.11, p=0.017)
Deprivation	1 (least deprived)	4468 (69.7)	1946 (30.3)	-	-
	2	5473 (70.0)	2345 (30.0)	0.98 (0.92-1.06, p=0.655)	0.97 (0.89-1.05, p=0.443)
	3	6751 (69.4)	2983 (30.6)	1.01 (0.95-1.09, p=0.680)	1.02 (0.94-1.11, p=0.590)
	4	7008 (70.2)	2982 (29.8)	0.98 (0.91-1.05, p=0.504)	1.06 (0.98-1.15, p=0.139)
	5 (most deprived)	8156 (72.8)	3052 (27.2)	0.86 (0.80-0.92, p<0.001)	1.01 (0.94-1.10, p=0.724)

Supplementary table 10D - Gastrointestinal and liver complications

Dependent: Self-care ability		Equivalent to before illness	Worse	OR (univariable)	OR (multilevel)
Gastrointestinal complication	No	29144 (71.4)	11650 (28.6)	-	-
	Yes	2722 (62.1)	1659 (37.9)	1.52 (1.43-1.63, p<0.001)	1.95 (1.82-2.10, p<0.001)
Age (years)	19-29	1121 (86.7)	172 (13.3)	-	-
	30-39	1962 (83.7)	383 (16.3)	1.27 (1.05-1.55, p=0.015)	1.57 (1.27-1.95, p<0.001)
	40-49	3331 (79.9)	838 (20.1)	1.64 (1.38-1.96, p<0.001)	2.14 (1.76-2.61, p<0.001)
	50-59	5508 (78.8)	1486 (21.2)	1.76 (1.49-2.09, p<0.001)	2.39 (1.97-2.89, p<0.001)
	60-69	5451 (73.3)	1990 (26.7)	2.38 (2.02-2.82, p<0.001)	3.37 (2.79-4.08, p<0.001)
	70-79	6237 (66.5)	3139 (33.5)	3.28 (2.79-3.88, p<0.001)	4.75 (3.94-5.73, p<0.001)
	80-89	6069 (61.4)	3810 (38.6)	4.09 (3.48-4.84, p<0.001)	6.14 (5.09-7.41, p<0.001)
	90+	1940 (58.5)	1377 (41.5)	4.63 (3.89-5.52, p<0.001)	7.12 (5.85-8.67, p<0.001)
Sex at Birth	Female	14550 (70.9)	5968 (29.1)	-	-
	Male	17230 (70.2)	7308 (29.8)	1.03 (0.99-1.08, p=0.107)	1.09 (1.04-1.14, p<0.001)
Deprivation	1 (least deprived)	4468 (69.7)	1946 (30.3)	-	-
	2	5473 (70.0)	2345 (30.0)	0.98 (0.92-1.06, p=0.655)	0.97 (0.90-1.05, p=0.470)
	3	6751 (69.4)	2983 (30.6)	1.01 (0.95-1.09, p=0.680)	1.05 (0.97-1.13, p=0.262)
	4	7008 (70.2)	2982 (29.8)	0.98 (0.91-1.05, p=0.504)	1.09 (1.01-1.18, p=0.023)
	5 (most deprived)	8156 (72.8)	3052 (27.2)	0.86 (0.80-0.92, p<0.001)	1.05 (0.97-1.13, p=0.272)

Supplementary table 10E - Cardiovascular complications

Dependent: Self-care ability		Equivalent to before illness	Worse	OR (univariable)	OR (multilevel)
Cardiovascular complication	No	28222 (72.7)	10600 (27.3)	-	-
	Yes	1957 (53.3)	1716 (46.7)	2.33 (2.18-2.50, p<0.001)	2.18 (2.02-2.35, p<0.001)
Age (years)	19-29	1121 (86.7)	172 (13.3)	-	-
	30-39	1962 (83.7)	383 (16.3)	1.27 (1.05-1.55, p=0.015)	1.69 (1.34-2.12, p<0.001)
	40-49	3331 (79.9)	838 (20.1)	1.64 (1.38-1.96, p<0.001)	2.26 (1.83-2.79, p<0.001)
	50-59	5508 (78.8)	1486 (21.2)	1.76 (1.49-2.09, p<0.001)	2.46 (2.00-3.01, p<0.001)
	60-69	5451 (73.3)	1990 (26.7)	2.38 (2.02-2.82, p<0.001)	3.34 (2.73-4.08, p<0.001)
	70-79	6237 (66.5)	3139 (33.5)	3.28 (2.79-3.88, p<0.001)	4.60 (3.77-5.62, p<0.001)
	80-89	6069 (61.4)	3810 (38.6)	4.09 (3.48-4.84, p<0.001)	5.91 (4.84-7.21, p<0.001)
	90+	1940 (58.5)	1377 (41.5)	4.63 (3.89-5.52, p<0.001)	6.74 (5.47-8.30, p<0.001)
Sex at Birth	Female	14550 (70.9)	5968 (29.1)	-	-
	Male	17230 (70.2)	7308 (29.8)	1.03 (0.99-1.08, p=0.107)	1.10 (1.05-1.15, p<0.001)
Deprivation	1 (least deprived)	4468 (69.7)	1946 (30.3)	-	-
	2	5473 (70.0)	2345 (30.0)	0.98 (0.92-1.06, p=0.655)	0.98 (0.90-1.06, p=0.554)
	3	6751 (69.4)	2983 (30.6)	1.01 (0.95-1.09, p=0.680)	1.03 (0.95-1.12, p=0.447)
	4	7008 (70.2)	2982 (29.8)	0.98 (0.91-1.05, p=0.504)	1.08 (1.00-1.18, p=0.051)
	5 (most deprived)	8156 (72.8)	3052 (27.2)	0.86 (0.80-0.92, p<0.001)	1.03 (0.95-1.12, p=0.507)

Supplementary table 10F - Neurological complications

Dependent: Self-care ability		Equivalent to before illness	Worse	OR (univariable)	OR (multilevel)
Neurological complication	No	29467 (72.4)	11216 (27.6)	-	-
	Yes	645 (37.5)	1074 (62.5)	4.37 (3.96-4.84, p<0.001)	4.35 (3.90-4.84, p<0.001)
Age (years)	19-29	1121 (86.7)	172 (13.3)	-	-
	30-39	1962 (83.7)	383 (16.3)	1.27 (1.05-1.55, p=0.015)	1.68 (1.33-2.10, p<0.001)
	40-49	3331 (79.9)	838 (20.1)	1.64 (1.38-1.96, p<0.001)	2.27 (1.84-2.81, p<0.001)
	50-59	5508 (78.8)	1486 (21.2)	1.76 (1.49-2.09, p<0.001)	2.47 (2.02-3.03, p<0.001)
	60-69	5451 (73.3)	1990 (26.7)	2.38 (2.02-2.82, p<0.001)	3.41 (2.78-4.17, p<0.001)
	70-79	6237 (66.5)	3139 (33.5)	3.28 (2.79-3.88, p<0.001)	4.73 (3.87-5.78, p<0.001)
	80-89	6069 (61.4)	3810 (38.6)	4.09 (3.48-4.84, p<0.001)	6.08 (4.98-7.43, p<0.001)
	90+	1940 (58.5)	1377 (41.5)	4.63 (3.89-5.52, p<0.001)	6.97 (5.66-8.60, p<0.001)
Sex at Birth	Female	14550 (70.9)	5968 (29.1)	-	-
	Male	17230 (70.2)	7308 (29.8)	1.03 (0.99-1.08, p=0.107)	1.10 (1.05-1.15, p<0.001)
Deprivation	1 (least deprived)	4468 (69.7)	1946 (30.3)	-	-
	2	5473 (70.0)	2345 (30.0)	0.98 (0.92-1.06, p=0.655)	0.98 (0.91-1.07, p=0.681)
	3	6751 (69.4)	2983 (30.6)	1.01 (0.95-1.09, p=0.680)	1.04 (0.96-1.13, p=0.327)
	4	7008 (70.2)	2982 (29.8)	0.98 (0.91-1.05, p=0.504)	1.11 (1.02-1.20, p=0.015)
	5 (most deprived)	8156 (72.8)	3052 (27.2)	0.86 (0.80-0.92, p<0.001)	1.03 (0.95-1.12, p=0.465)

Supplementary table 10G - Respiratory complications

Dependent: Self-care ability		Equivalent to before illness	Worse	OR (univariable)	OR (multilevel)
Respiratory complication	No	26878 (74.2)	9360 (25.8)	-	-
	Yes	3380 (52.4)	3069 (47.6)	2.61 (2.47-2.75, p<0.001)	3.61 (3.39-3.84, p<0.001)
Age (years)	19-29	1121 (86.7)	172 (13.3)	-	-
	30-39	1962 (83.7)	383 (16.3)	1.27 (1.05-1.55, p=0.015)	1.58 (1.25-1.99, p<0.001)
	40-49	3331 (79.9)	838 (20.1)	1.64 (1.38-1.96, p<0.001)	2.02 (1.63-2.50, p<0.001)
	50-59	5508 (78.8)	1486 (21.2)	1.76 (1.49-2.09, p<0.001)	2.22 (1.80-2.72, p<0.001)
	60-69	5451 (73.3)	1990 (26.7)	2.38 (2.02-2.82, p<0.001)	3.24 (2.64-3.98, p<0.001)
	70-79	6237 (66.5)	3139 (33.5)	3.28 (2.79-3.88, p<0.001)	4.90 (4.00-6.00, p<0.001)
	80-89	6069 (61.4)	3810 (38.6)	4.09 (3.48-4.84, p<0.001)	6.63 (5.42-8.12, p<0.001)
	90+	1940 (58.5)	1377 (41.5)	4.63 (3.89-5.52, p<0.001)	7.59 (6.14-9.38, p<0.001)
Sex at Birth	Female	14550 (70.9)	5968 (29.1)	-	-
	Male	17230 (70.2)	7308 (29.8)	1.03 (0.99-1.08, p=0.107)	1.06 (1.02-1.11, p=0.009)
Deprivation	1 (least deprived)	4468 (69.7)	1946 (30.3)	-	-
	2	5473 (70.0)	2345 (30.0)	0.98 (0.92-1.06, p=0.655)	0.98 (0.90-1.06, p=0.614)
	3	6751 (69.4)	2983 (30.6)	1.01 (0.95-1.09, p=0.680)	1.03 (0.95-1.12, p=0.419)
	4	7008 (70.2)	2982 (29.8)	0.98 (0.91-1.05, p=0.504)	1.08 (1.00-1.17, p=0.062)
	5 (most deprived)	8156 (72.8)	3052 (27.2)	0.86 (0.80-0.92, p<0.001)	1.04 (0.96-1.13, p=0.366)

Supplementary Figures

Supplementary figure 1 – Heatmap of co-occurence of complications. Measured using the Jaccard similarity index, where 1 is perfect co-occurrence and 0 is no co-occurrence.

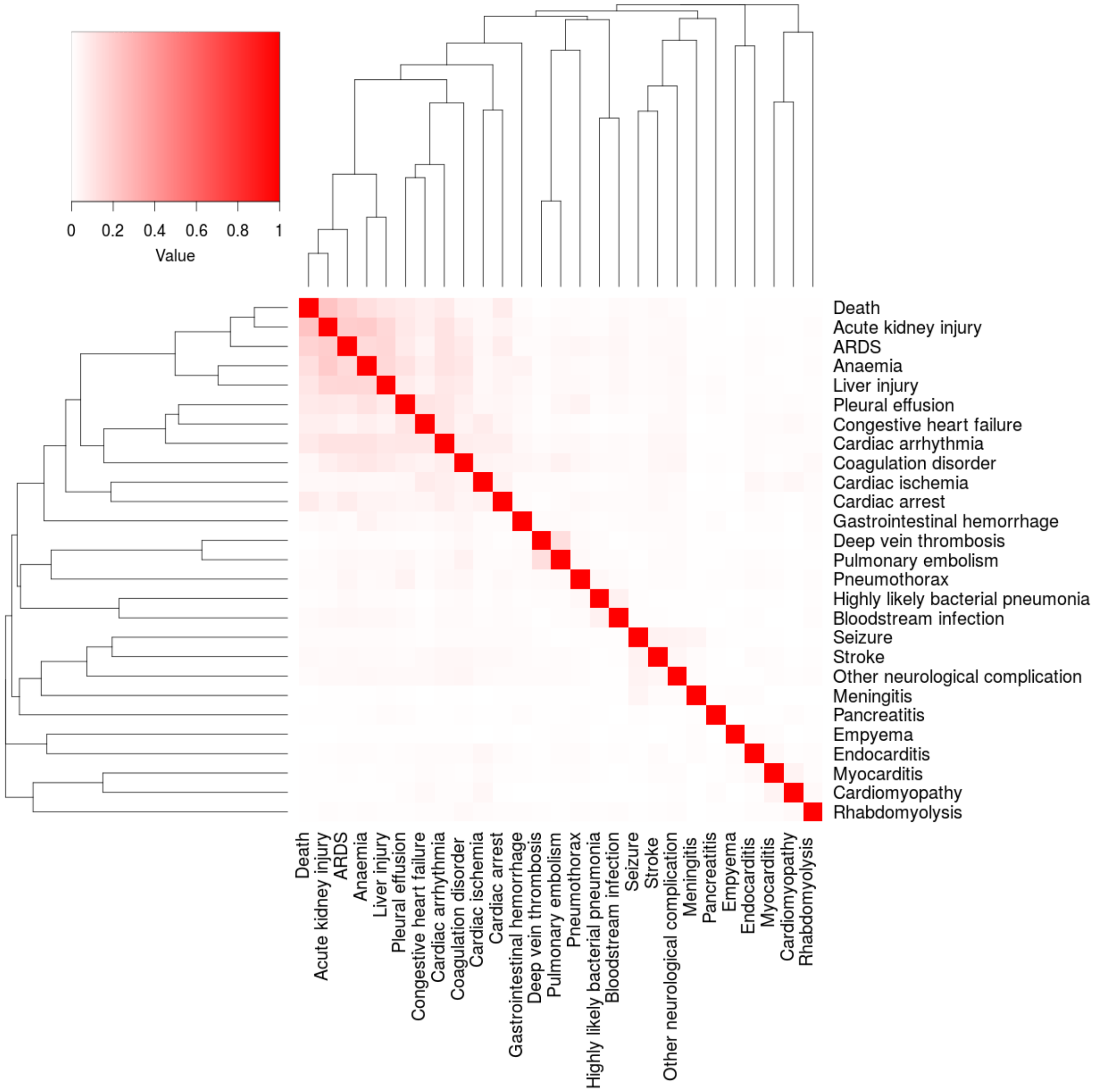

Supplementary figure 2 - Adjusted effect of age and sex on organ specific complications in adults with severe COVID-19 (also adjusted for centre as a random effect).

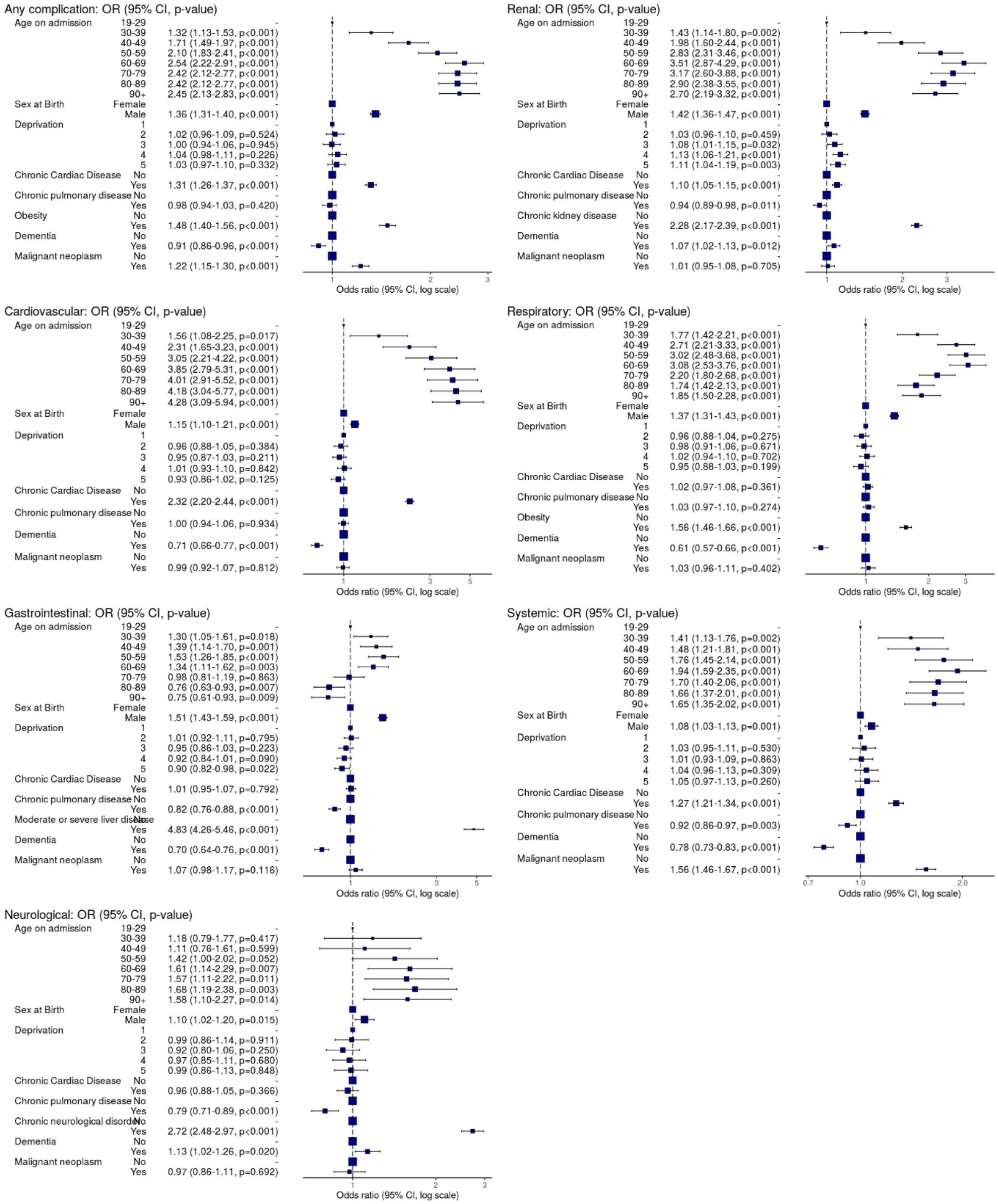

Supplementary figure 3 - Effect of age, sex and deprivation on specific complications in adults with COVID-19 (also adjusted for centre as a random effect).

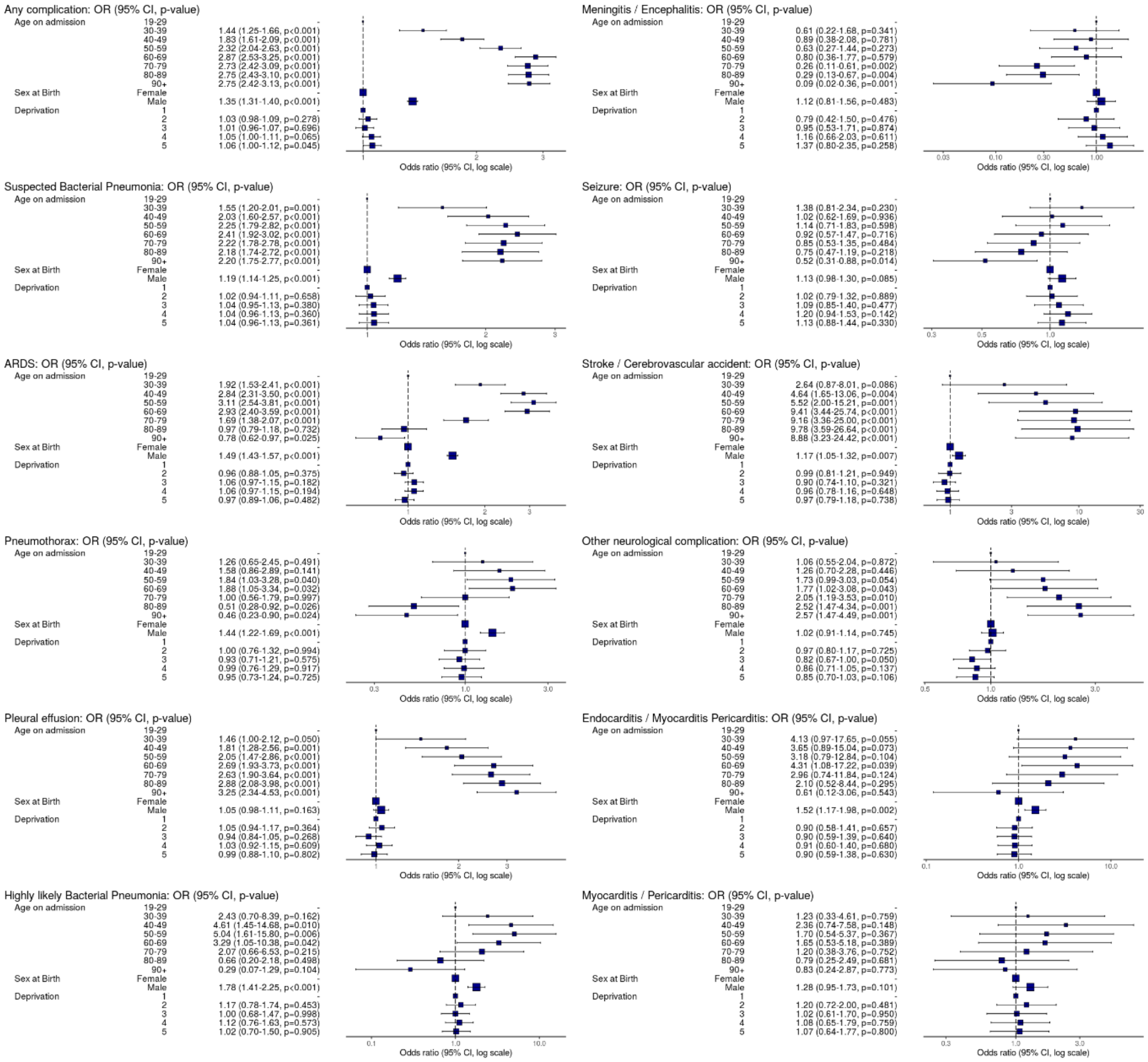

Supplementary figure 3 (continued) - Effect of age, sex and deprivation on specific complications in adults with COVID-19 (also adjusted for centre as a random effect).

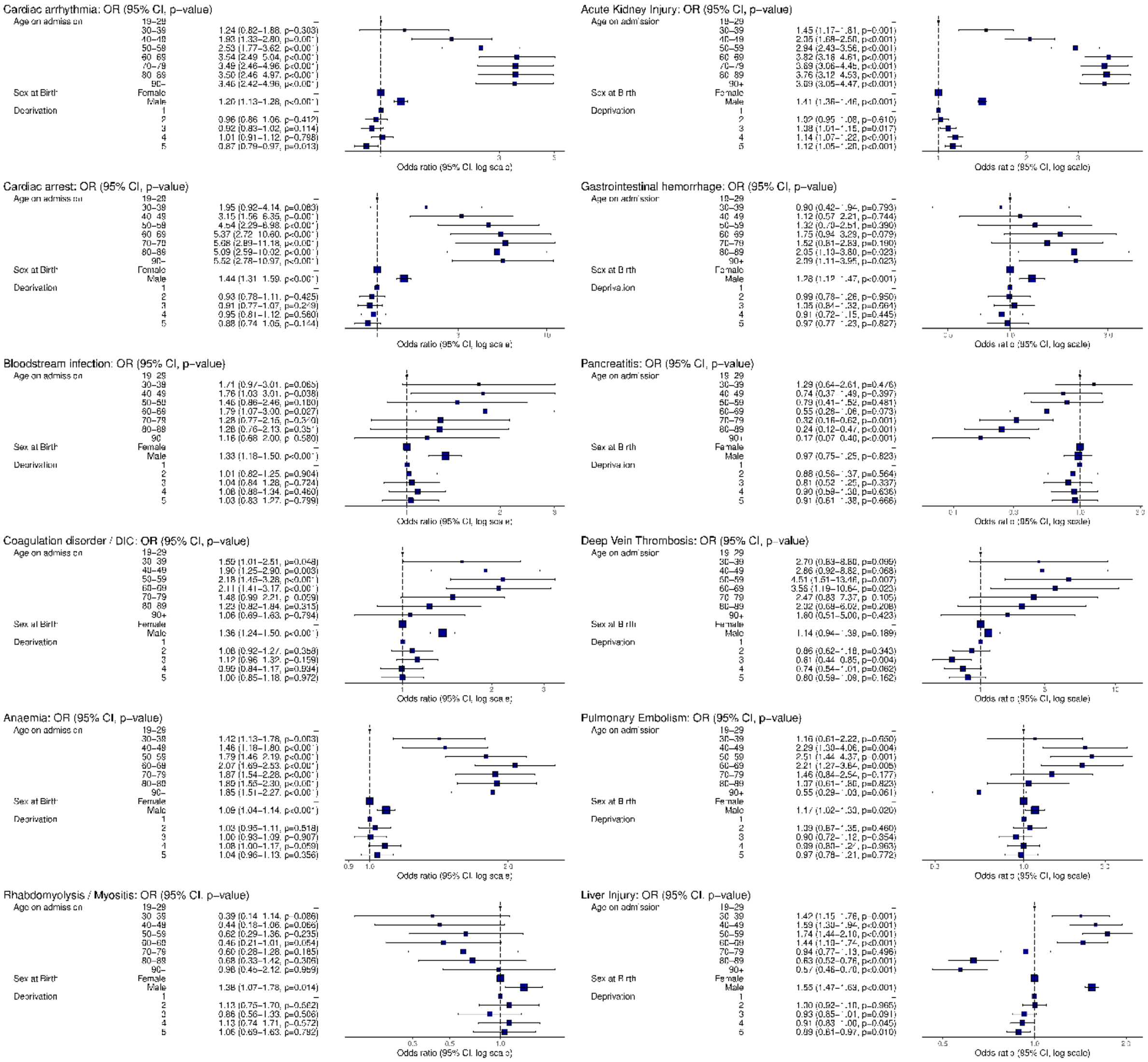

DIC- Disseminated intravascular coagulation.

Supplementary figure 4 - Relationship between age, sex, comorbidities and adjusted outcomes using generalised additive models. (A) Shows relationships for the outcome of adjusted risk of systemic complications. (B) Shows relationships for the outcome of adjusted mortality risk, stratified by presence of systemic complications. Each line represents one bootstrap replicate (i.e. one simulated patient).

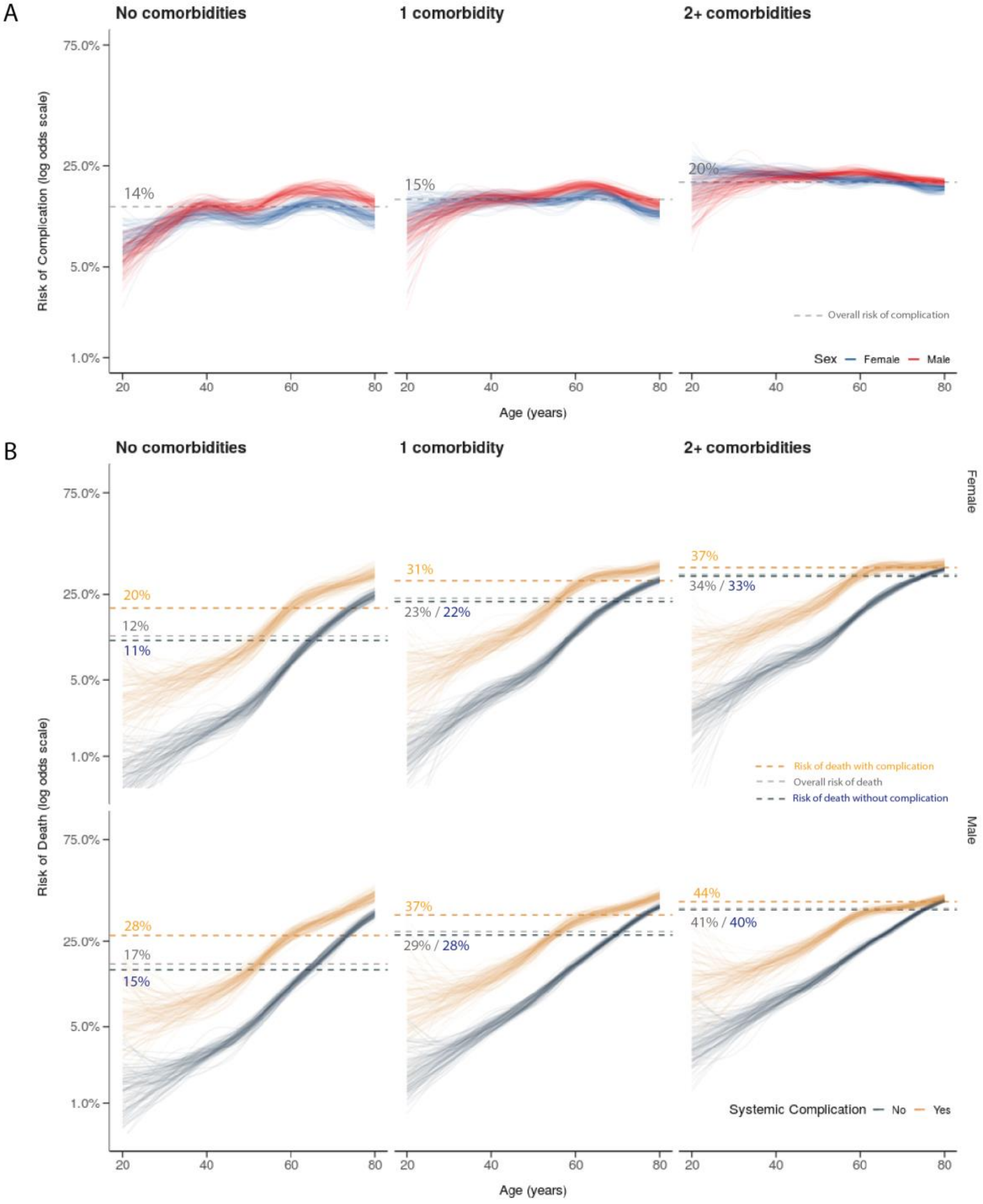

Supplementary figure 5 - Relationship between age, sex, comorbidities and adjusted outcomes using generalised additive models. (A) Shows relationships for the outcome of adjusted risk of renal complications. (B) Shows relationships for the outcome of adjusted mortality risk, stratified by presence of renal complications. Each line represents one bootstrap replicate (i.e. one simulated patient).

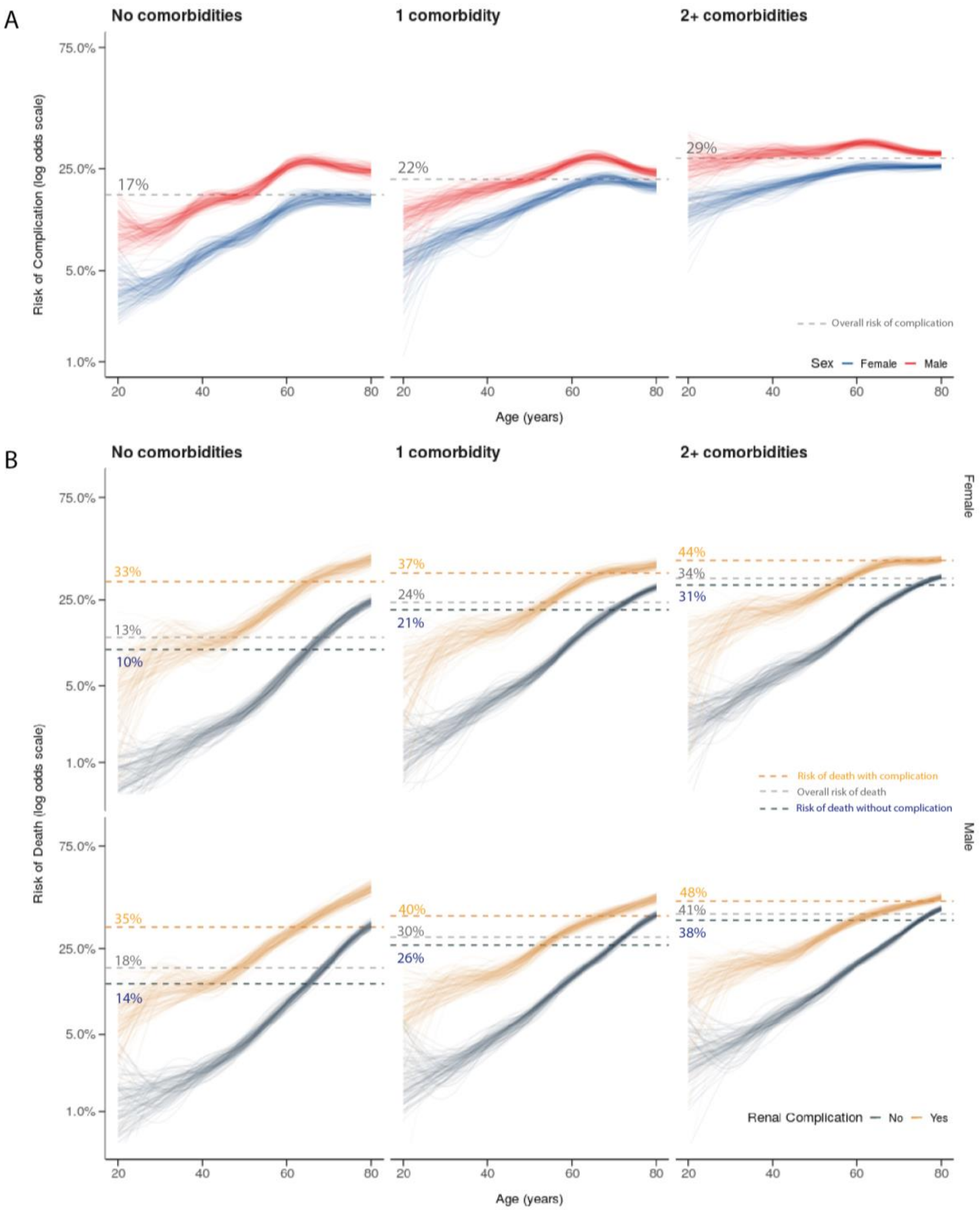

Supplementary figure 6 - Relationship between age, sex, comorbidities and adjusted outcomes using generalised additive models. (A) Shows relationships for the outcome of adjusted risk of gastrointestinal or liver complications. (B) Shows relationships for the outcome of adjusted mortality risk, stratified by presence of gastrointestinal or liver complications. Each line represents one bootstrap replicate (i.e. one simulated patient).

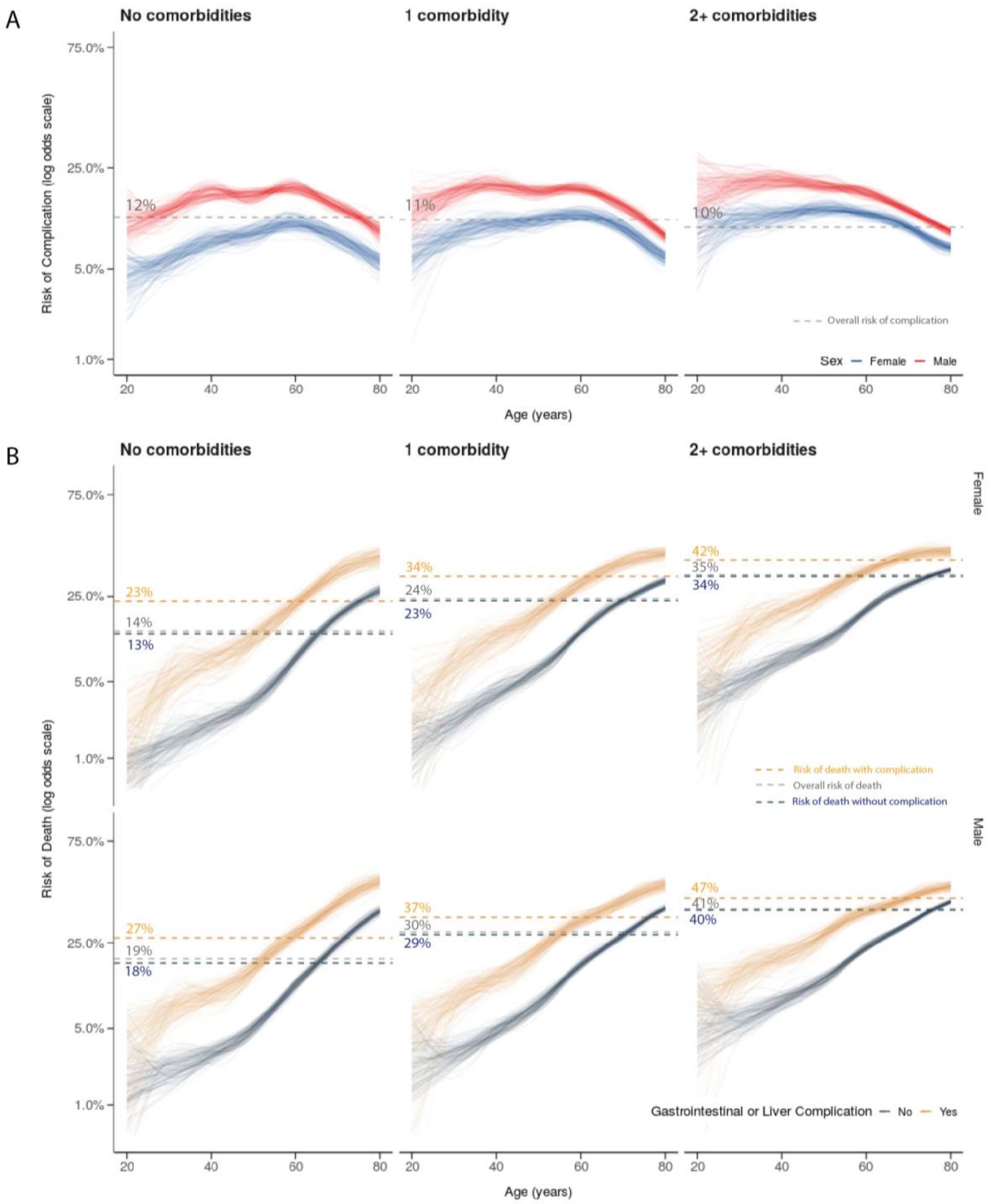

Supplementary figure 7 - Relationship between age, sex, comorbidities and adjusted outcomes using generalised additive models. (A) Shows relationships for the outcome of adjusted risk of cardiovascular complications. (B) Shows relationships for the outcome of adjusted mortality risk, stratified by presence of cardiovascular complications. Each line represents one bootstrap replicate (i.e. one simulated patient).

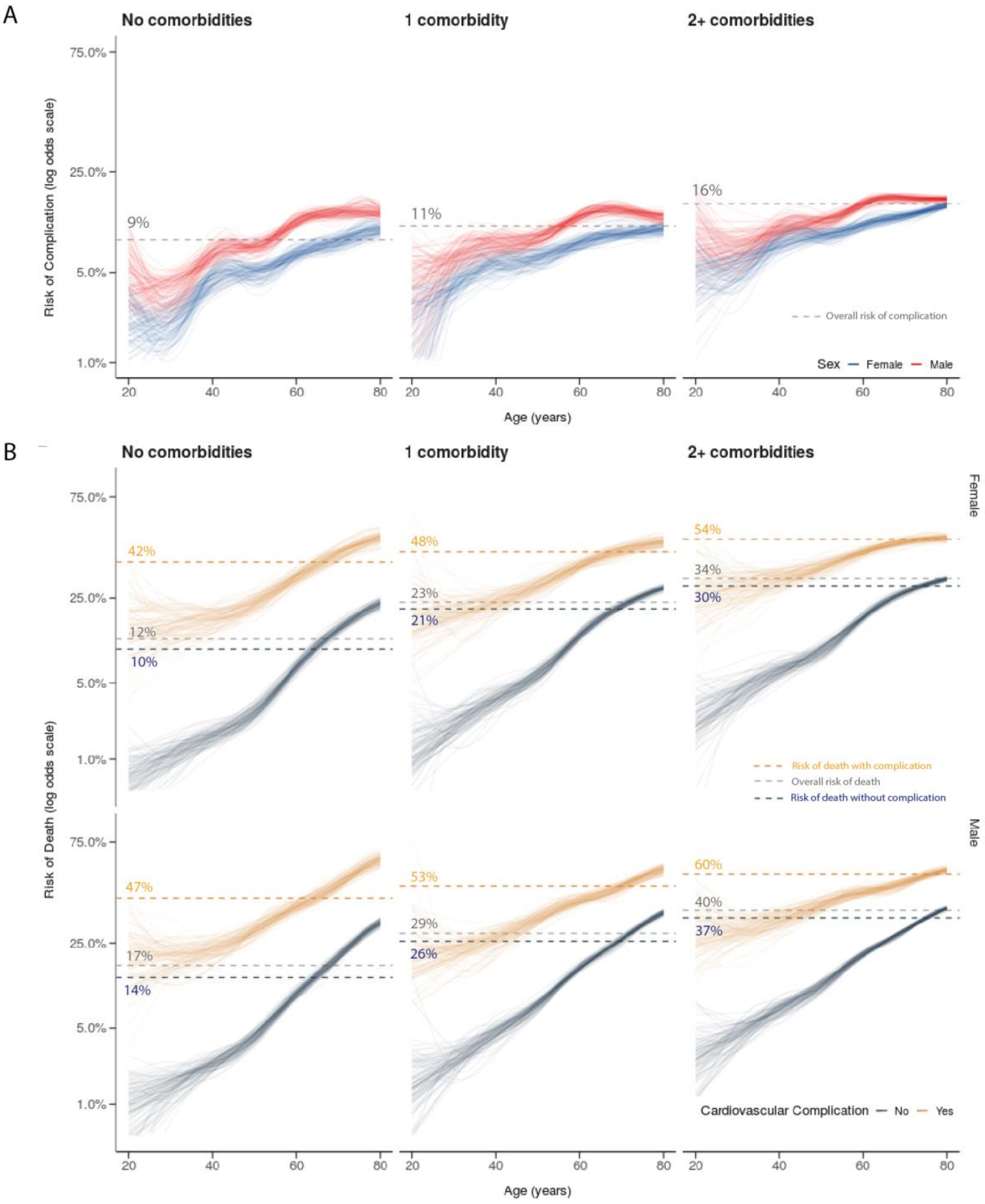

Supplementary figure 8 - Relationship between age, sex, comorbidities and adjusted outcomes using generalised additive models. (A) Shows relationships for the outcome of adjusted risk of neurological complications. (B) Shows relationships for the outcome of adjusted mortality risk, stratified by presence of neurological complications. Each line represents one bootstrap replicate (i.e. one simulated patient).

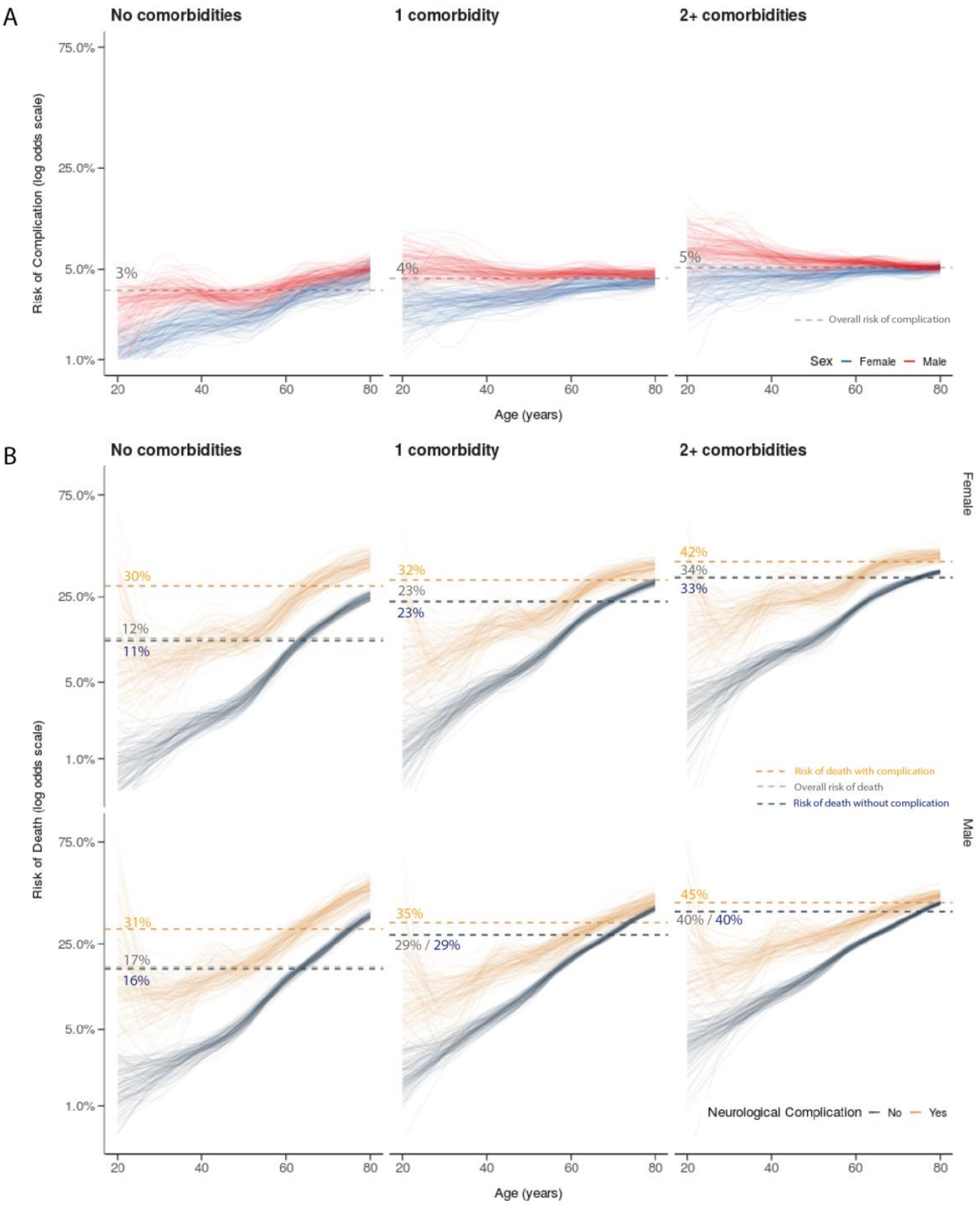

Supplementary figure 9 - Relationship between age, sex, comorbidities and adjusted outcomes using generalised additive models. (A) Shows relationships for the outcome of adjusted risk of respiratory complications. (B) Shows relationships for the outcome of adjusted mortality risk, stratified by presence of respiratory complications. Each line represents one bootstrap replicate (i.e. one simulated patient).

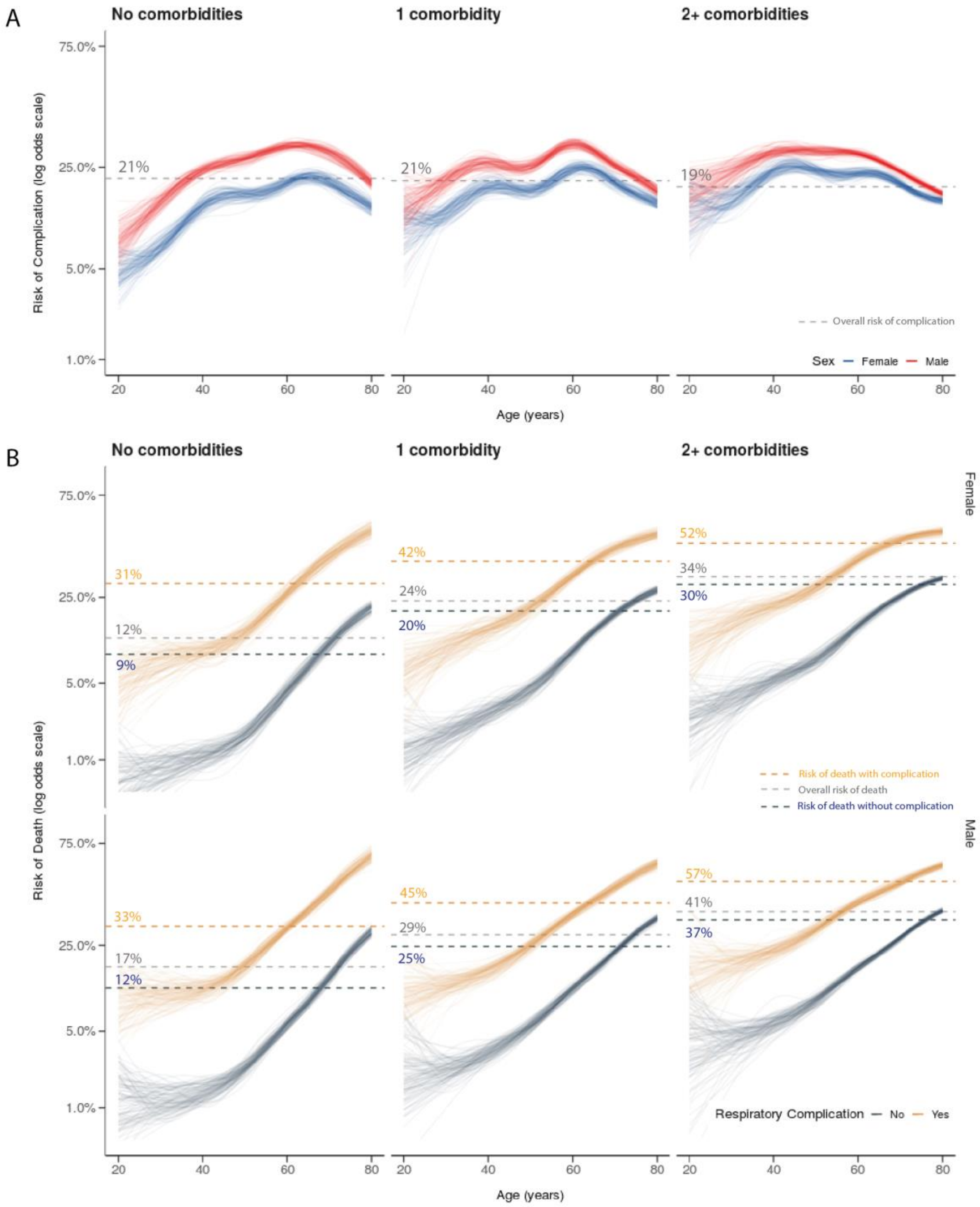

Supplementary figure 11 - Associations between specific comorbidities and organ specific complications in adults with severe COVID-19.

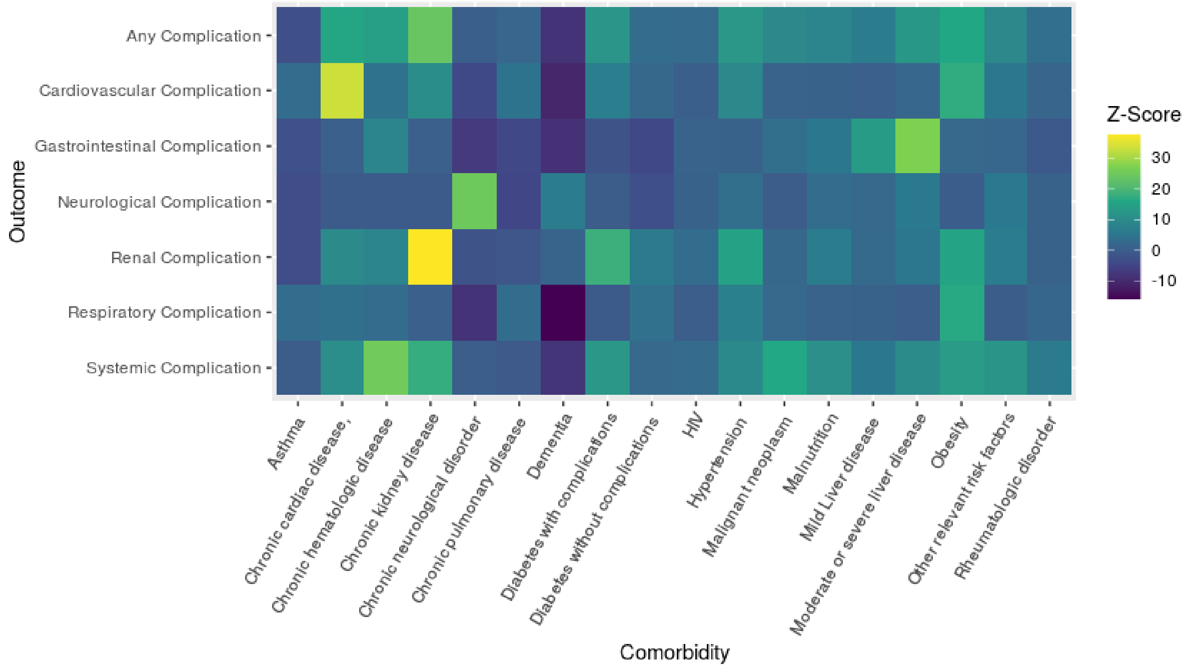

A positive Z-score represents stronger associations between variables in positive direction, whereas negative z-score represents effect in opposite direction.

Supplementary figure 12 - Effect of specific comorbidities on specific complications in adults with COVID-19.

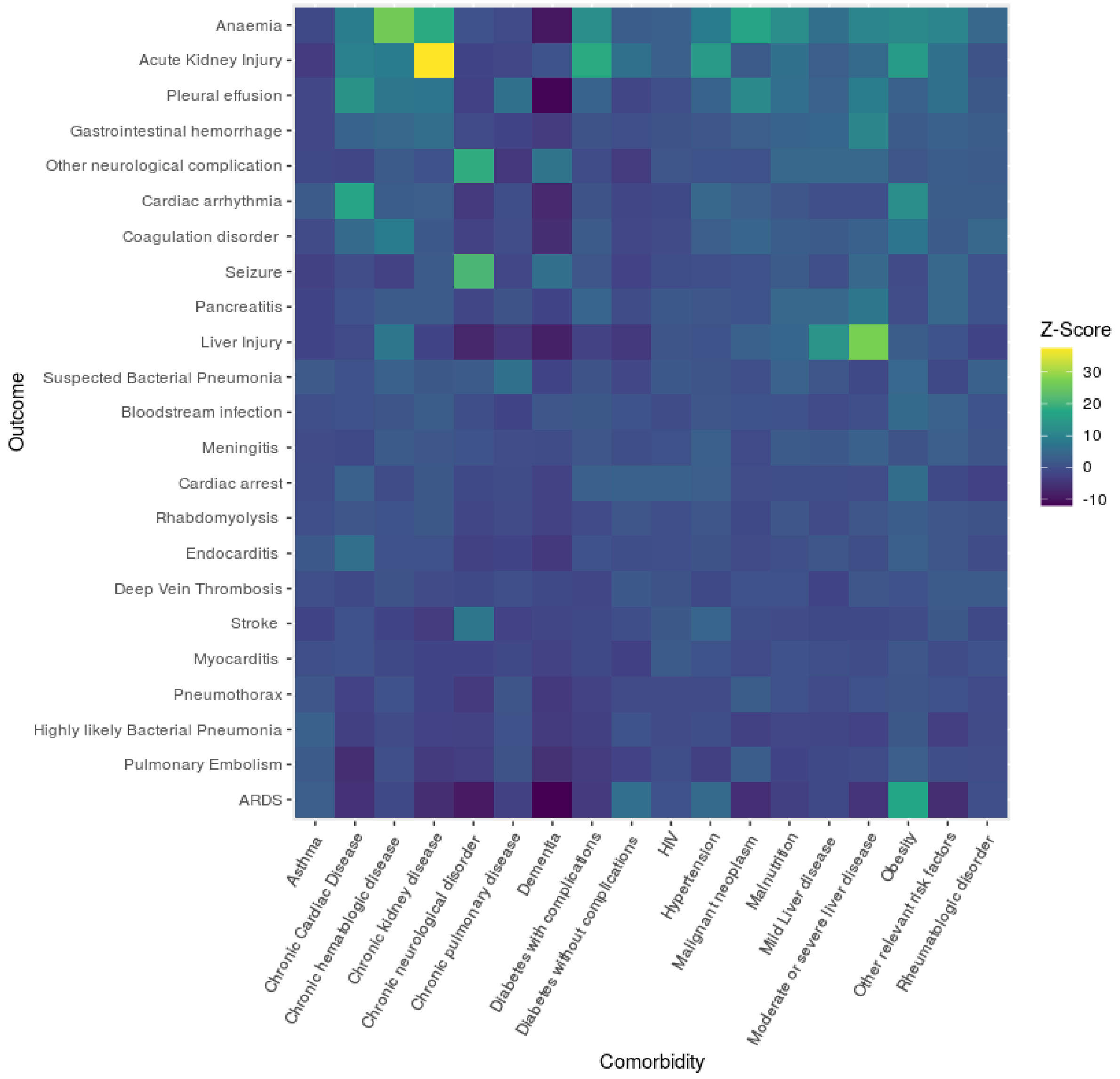

A positive Z-score represents stronger associations between variables in positive direction, whereas negative z-score represents effect in opposite direction.

Supplementary figure 13 – Probability of complication by admission severity score and symptoms in those who survived. (A) ISARIC 4C mortality score against proportion of patients experiencing any complication. (B) National Early Warning Score 2 against proportion of patients experiencing any complication. (C) Quick Sequential Organ Failure Assessment score against proportion of patients experiencing any complication. (D) Total number of symptoms on admission against proportion of patients experiencing any complication. For continuous outcomes (4C score, NEWS2 and number of symptoms), red line shows smoothed conditional mean line using generalised additive model. Higher scores indicate critical illness.

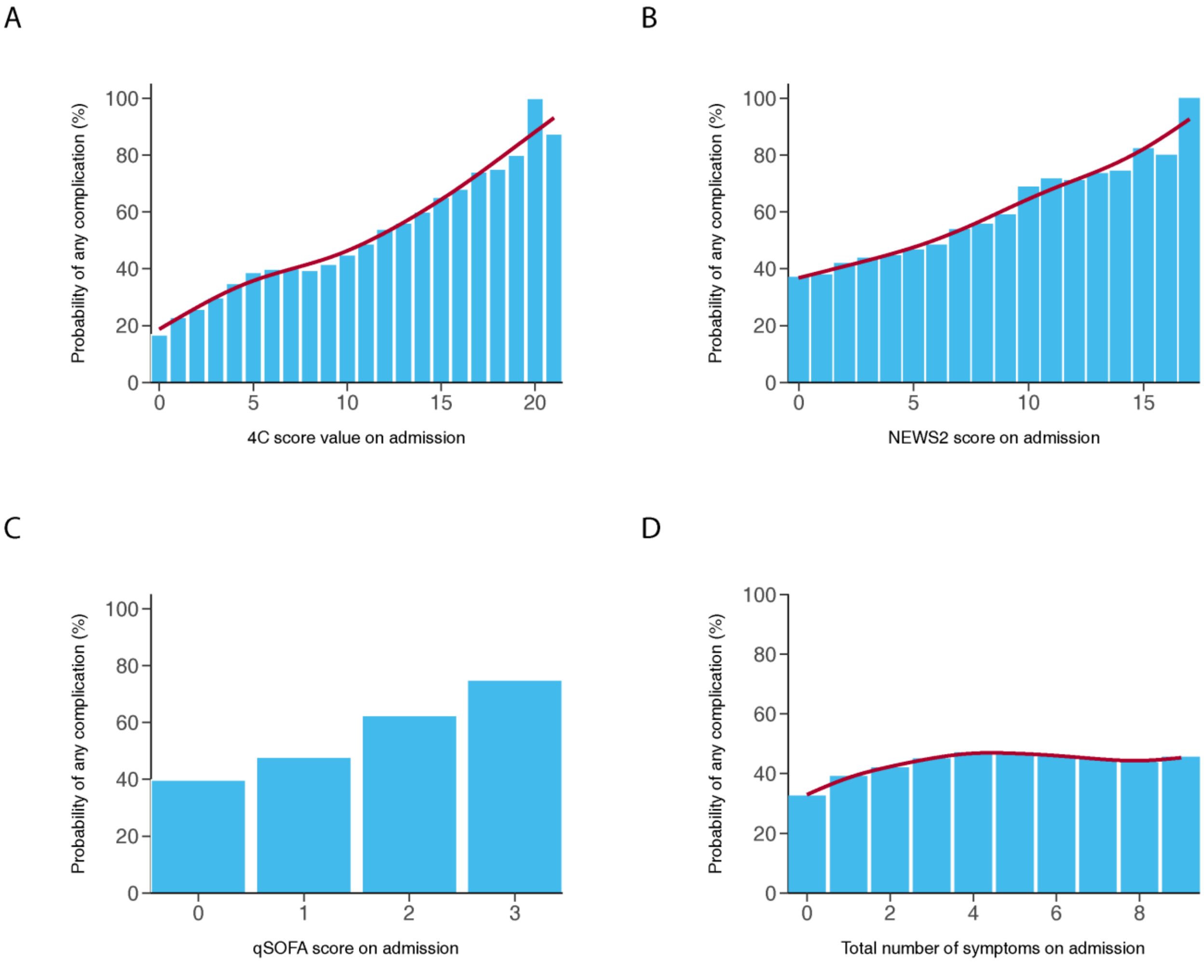

Supplement: Supplementary appendix [file mmc1.pdf]
